# Frame‐Shifted Synthesis of Oligoheterocycles as a Platform for Molecular Design

**DOI:** 10.1002/anie.202520677

**Published:** 2025-11-17

**Authors:** Kane A. C. Bastick, Caleb E. Griesbach, Ben Zhen Huang, Morgan J. Cordell, Yang (Daniel) Ou, Alán Aspuru‐Guzik, Andrei K. Yudin

**Affiliations:** ^1^ Department of Chemistry University of Toronto Toronto ON M5S 3H6 Canada; ^2^ Department of Computer Science University of Toronto Toronto ON M5S 2E4 Canada; ^3^ Acceleration Consortium Toronto ON M7A 2S4 Canada; ^4^ Vector Institute for Artificial Intelligence Toronto ON M5G 1M1 Canada; ^5^ Department of Chemical Engineering and Applied Chemistry University of Toronto Toronto ON M5S 3E5 Canada; ^6^ Department of Materials Science and Engineering University of Toronto Toronto ON M5S 3E4 Canada

**Keywords:** Cross‐condensation, Frame‐shifted synthesis, Heterocycles, Synthesis design, Synthetic methods

## Abstract

Chemists continue to develop strategies that leverage abundant feedstocks to accelerate the production of medicines, agrochemicals, and materials. Oligoheterocycles are a broad class of molecules that span these disciplines. Cross‐coupling strategies are commonly used to assemble oligoheterocycles; however, these methods can be challenging and often rely on precious metal catalysts and building blocks whose production entails significant environmental and economic costs. We present a strategy for the iterative construction of oligoheterocycles that leverages sustainable building blocks designed around high‐oxidation‐state carbon. By utilizing feedstocks from the base of the chemical supply chain, we generate bifunctional building blocks that chemoselectively engage with a variety of partners through simple condensation manifolds. This approach has enabled the creation of novel scaffolds previously inaccessible by conventional means, exemplifying sustainable design strategies.

## Graphical abstract

Modern chemistry seeks to turn the simple raw materials into complex molecules that matter in health, agriculture, and technology. Among these, oligoheterocycles stand out for their versatility, yet they are most often built by cross‐coupling reactions that often rely on expensive metal catalysts. We outline a complementary path: an iterative construction platform that draws on high‐oxidation‐state carbon modules derived from commodity feedstocks. The bifunctional units we describe participate in condensation sequences and counterintuitively open routes to frameworks beyond the reach of traditional methods.

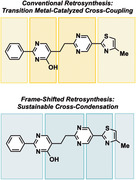

## Introduction

**Figure 1:**
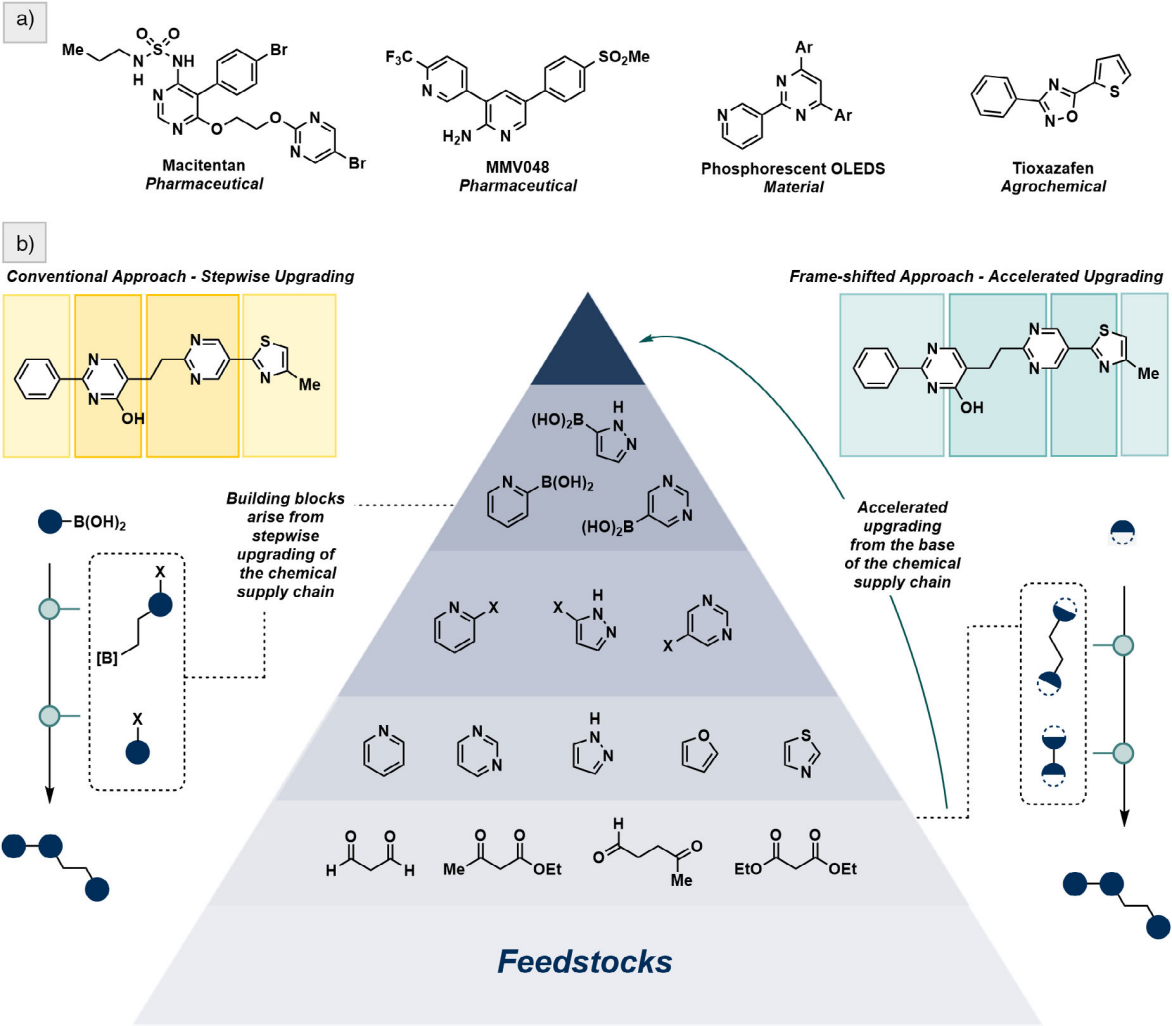
Strategies for oligoheterocycle synthesis and their relation to chemical supply chains. a) Representative oligoheterocycles among therapeutic agents, materials, and agrochemicals. b) Traditional stepwise synthesis via cross‐coupling of building blocks. c) This work: accelerated upgrading from the base of the chemical supply chain using a cross‐condensation strategy.

Chemical synthesis aims to upgrade basic chemical feedstocks into molecules of higher complexity. This happens at different scales, ranging from laboratory to industrial production, and relies on the global supply chain of molecular building blocks.^[^ ^1^ ^]^ Various factors, including climate change, resource scarcity, economic sanctions, armed conflicts, and geopolitical tensions, can hinder access to the building blocks required to produce value‐added compounds.^[^ ^2^, ^3^, ^4^, ^5^, ^6^ ^]^ In particular, the vulnerability of modern supply chains is a significant challenge for chemists attempting to synthesize oligoheterocycles amid ongoing disruptions.^[^ ^7^ ^]^ It is incumbent upon the chemistry community to develop robust strategies and methods that utilize abundant feedstocks to accelerate the delivery of essential medicines, agrochemicals, and materials.^[^ ^6^ ^]^ Here we demonstrate a synthetic strategy to generate oligoheterocycles, a broad class of molecules with applications that range from drug discovery to agrochemistry and materials science (Figure 1a). Our workflow exemplifies an accelerated upgrading of small molecular weight building blocks that are designed using carbon atoms of high oxidation state. Nature's preference for oxidized carbon—evident in the centrality of carbonyls and carboxylic acids in metabolism and biosynthesis—is not merely biochemical contingency; it reflects a deeper reactivity logic that emphasizes redox‐neutral condensation reactions. Accordingly, the synthetic components in our building blocks are chosen for their reliable reactivity patterns. Their convergence in frame‐shifted synthesis gives rise to an enabling strategy that repurposes classical reactions for modular molecular construction based on the principles of supply chain resilience and sustainable design.^[^ ^8^ ^]^

Transition metal‐catalyzed cross‐coupling reactions between heteroaromatic building blocks represent a common strategy to access oligoheterocycles.^[^ ^9^, ^10^, ^11^, ^12^ ^]^ A representative disconnection approach in this chemistry can be illustrated using colored frames (Figure 1b), each corresponding to a particular building block. This visual device reflects the underlying strategic thinking, in which synthesis is planned around connecting heterocyclic modules that correspond to metal‐catalyzed cross‐coupling operations. A typical sequence in this scenario comprises the use of chemical commodities in heterocycle formation,^[^ ^13^, ^14^, ^15^ ^]^ halogenation, and installation of a metalloid (borylation, silylation, germylation, etc.), followed by the cross‐coupling of the resulting fragments.^[^ ^16^, ^17^, ^18^ ^]^ This strategy is broadly applicable, leading to the increased demand for heterocyclic building blocks with bespoke functional handles.^[^ ^11^, ^19^ ^]^ We previously attempted a hybrid building block approach that was designed to merge condensation reactions with strategic installation of a carbon–boron bond for cross‐coupling. Amphoteric molecules that featured carbon–boron bonds led to borylated heterocycles at positions that are difficult to obtain through other means.^[^ ^20^, ^21^ ^]^ However, the final cross‐coupling step proved challenging due to competing protodemetalation and other side processes.^[^ ^22^, ^23^, ^24^, ^25^, ^26^ ^]^ As a departure from this approach, which proved conceptually interesting but ultimately limiting, we have now arrived at a retrosynthetic “frame shift” (Figure 1c) that employs cross‐condensation to address chemoselectivity challenges and reduce dependence on costly precious metals, ligands, and bespoke building blocks required for catalysis. This strategy relies on high‐oxidation‐state building blocks derived from feedstocks located at the base of the chemical supply chain. To prove that the idea of sustainable discovery has promise, we show how cross‐condensation can lead to oligoheterocyclic scaffolds that have so far eluded classical disconnection approaches.

## Results and Discussion

**Figure 2:**
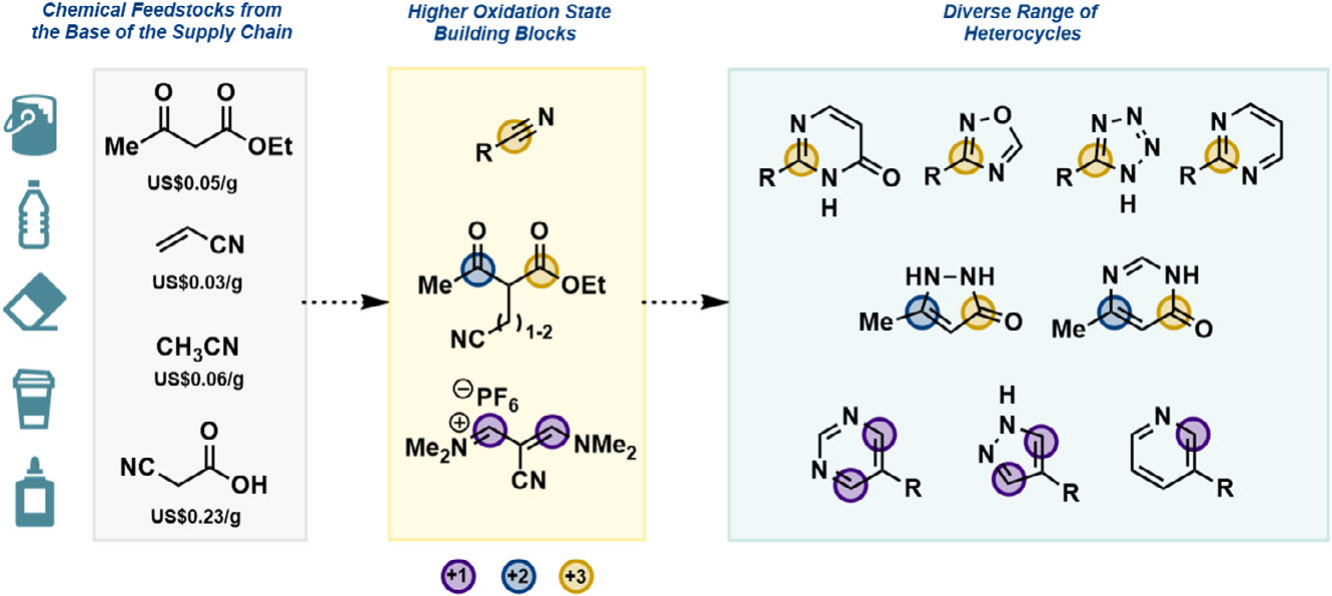
Sustainable feedstocks that yield bifunctional building blocks. Each building block consists of two half‐heterocycles containing high‐oxidation‐state carbons that are key precursors for heterocyclic target structures.

We envisioned that cross‐condensations could be used to forge oligoheterocycles using a half‐heterocycle strategy, where heterocycles would arise from the electrophilic and nucleophilic ends of a bifunctional building block (Figure 2). We aimed to avoid protecting groups that are typically employed in bifunctional building blocks (e.g., Fmoc‐protected amino acids). This meant searching for the fragments that could participate in chemoselective reactions and connecting them using linkers of varying sizes. During our survey of the literature, we noted that the oxidation state of carbon atoms in a half‐heterocycle fragment is typically preserved; therefore, simply varying the cross‐condensation partner would form a different heterocycle. We arrived at 1,3‐dialdehyde surrogate in the form of a vinamidinium salt^[^ ^27^, ^28^, ^29^ ^]^ and β‐ketoester as appropriate electrophilic half‐heterocycles. 1,3‐Dialdehydes generally polymerize, while vinamidinium salts offer a stable alternative to prepare pyrazoles,^[^ ^30^ ^]^ pyrimidines,^[^ ^31^, ^32^ ^]^ pyridines,^[^ ^33^, ^34^, ^35^ ^]^ and pyrroles.^[^ ^36^ ^]^ Although other synthetic equivalents of 1,3‐dialdehydes are also available, we recognized that vinamidinium salts, sourced from acetic acid (produced from both petrochemical and biorefinery supply chains)^[^ ^37^, ^38^ ^]^ could be substituted with a linchpin to forge a second heterocycle. β‐Ketoesters are used extensively to prepare coumarins,^[^ ^39^ ^]^ pyrazolones,^[^ ^40^ ^]^ pyrroles,^[^ ^41^ ^]^ pyrimidines,^[^ ^42^ ^]^ pyridines,^[^ ^43^ ^]^ and quinolines,^[^ ^44^, ^45^ ^]^ at low cost. Ethyl acetoacetate, the simplest β‐ketoester, costs ∼US$ 0.05 per g and is a bulk fine chemical used for pharmaceutical synthesis, flavoring agents, lacquers, and paints. A critical part of our approach is for half‐heterocycle components to act orthogonally to each other. We conceived an “arming strategy” that relies on operationally simple chemistry to reveal the second half‐heterocycle. Nitrile was chosen as the ideal ambidivergent linchpin—with nucleophilic and electrophilic capabilities exposed during arming—to incorporate into the β‐ketoester and vinamidinium salt building blocks. The nitrile functionality was meant to deliver a +3 oxidation state that could be unmasked to yield a wide variety of different half‐heterocycles. Unlike carboxylic acids used in SPPS, this direct nitrile arming strategy was devised to omit the requirement for protecting group installation/removal steps. Hydroxylamine, lithium bis(trimethylsilyl)amide (LiHMDS), and sodium hydrogen sulfide were selected as operationally simple reagents to produce oxadiazoles via aminooximes, pyrimidines or pyrimidones via amidines, and thiazoles via thioamides, respectively.

**Figure 3:**
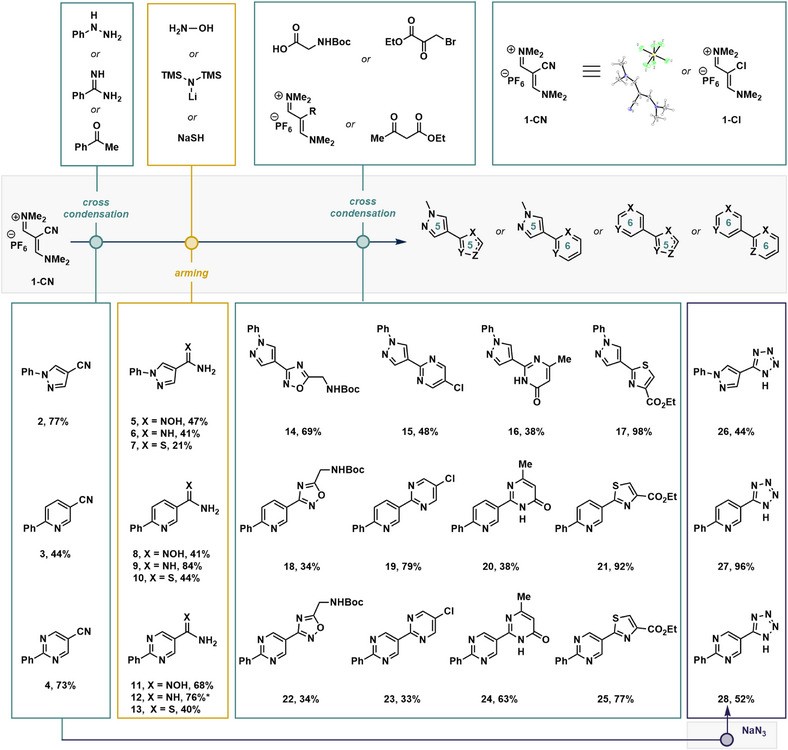
Synthesis of bis‐heterocycles using a nitrile arming/condensation strategy from vinamidinium salts. Isolated yields are reported. For detailed experimental details, see Supporting Information. *Isolated as formic acid salt.

We began by demonstrating some of the capabilities of our cross‐condensation strategy using vinamidinium/nitrile conjugates (Figure 3). A nitrile‐based vinamidinium salt **1‐CN** was prepared directly from cyanoacetic acid (US$ 0.23 per g), a bulk chemical involved in the super glue and synthetic caffeine supply chains.^[^ ^46^ ^]^ Cross‐condensation (left panel), nitrile arming (left‐center panel), and then a second cross‐condensation (Figure 3, right‐center panel) yielded the bis‐heterocycles in three operations. The pyrazole nitrile **2** and pyrimidine nitrile **4** could be prepared by condensations between vinamidinium salt **1‐CN** and commercially available phenyl hydrazine or benzamidine, respectively. These compounds could be isolated in 73%–77% yield by simple precipitation without the requirement for chromatography. Preparing pyridine nitrile **3** from **1‐CN,** acetophenone, and ammonium acetate based off similar construction strategies using vinamidinium salts was more challenging and required a solvent swap to prevent significant byproduct formation (for further details, see Supporting Information). Attempts to produce an equivalent pyridone nitrile using a similar unified strategy were met with poor outcomes. With heterocyclic nitriles **2**–**4** in hand, we moved on to the arming phase to unmask the nitrile into a nucleophilic half‐heterocycle (Figure 3, left‐center panel). Aminooximes (**5, 8**, and **11**) were prepared by addition of hydroxylamine in ethanol to the nitrile and resulted in moderate to good yields (41%–68%). Amidines (**6, 9**, and **12**) were prepared in moderate to excellent yields (41%–84%) by the addition of LiHMDS as a solution in THF (in air and at room temperature), followed by quenching of the bis(trimethylsilylated) amidine with methanolic hydrogen chloride. Thioamides (**7**, **10**, and **13**) were prepared by treating the nitriles with sodium hydrosulfide and magnesium chloride in acceptable yields for later operations (21%–44%). With the three nucleophilic half‐heterocycles for each of the three classes in hand (i.e., **5**–**13**), attention was turned to the final bis‐heterocycle forming steps (Figure 3, right‐center panel). We opted to prioritize the simplicity of purification, which typically involved trituration of the final compound with water with no workup or column. At demonstration scale, yields occasionally varied for the same operation across each bis‐heterocycle family. Heterocycle‐oxadiazoles **14**, **18**, and **22** could be prepared in 34%–69% yield from the parent aminooximes (i.e., **5, 8**, and **11**) using Boc‐Gly‐OH in an amide coupling followed by dehydration, with an aminomethylene group available for further derivatization. Heterocyclic pyrimidines **15**, **19**, and **23** were synthesized from the corresponding parent amidines (**6**, **9**, and **12**) and chlorovinamidinium salt **1‐Cl** with generally high conversions. However, purification of compounds **15** and **23** proved difficult due to solubility issues, complicating both precipitation and chromatographic methods and leading to variable yields for this series (33%–79%). A similar effect was observed for the heterocycle‐pyrimidones (**16**, **20**, and **24**), produced from ethyl acetoacetate (38%–63%). Heterocycle‐thiazoles (**17**, **21**, and **25**) were produced in generally excellent yields (77%–98%) from the parent thioamides (i.e., **7**, **10**, and **13**) and ethyl bromopyruvate but required purification via column chromatography. Finally, tetrazoles **26**–**28** could be readily formed from the left‐panel nitriles (i.e., **2**–**4**) and sodium azide with ammonium chloride. The novelty of these bis(heterocycle) combinations is discussed in later sections.

**Figure 4:**
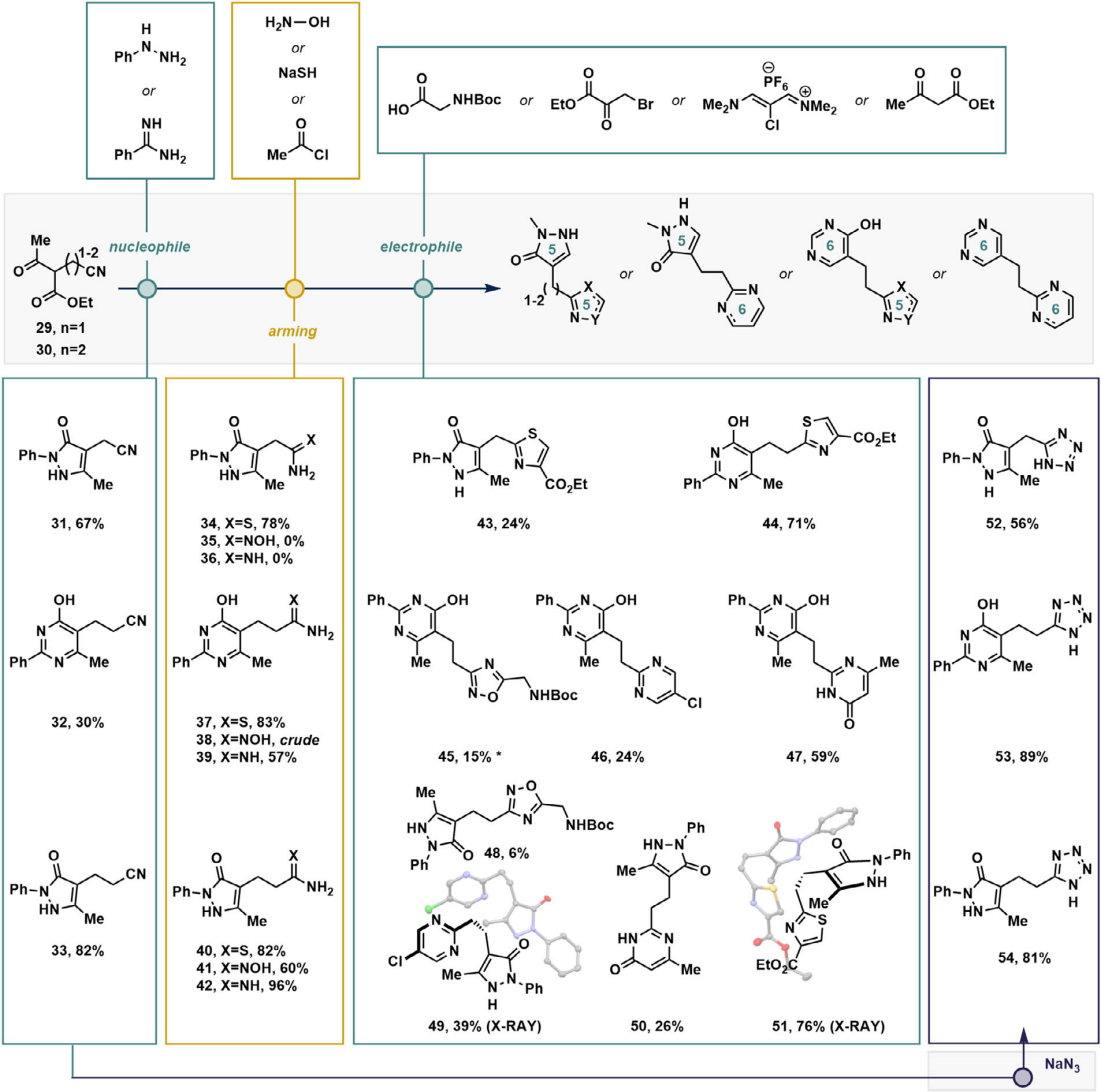
Synthesis of C1‐ and C2‐linked bis‐heterocycles using a nitrile arming/condensation strategy from β‐ketoesters. Isolated yields are reported. For detailed experimental details, see Supporting Information. *Yield over two steps.

We next turned our attention to exploring some of the capabilities of our strategy using β‐ketoesters to prepare a variety of pyrazolone‐ and pyrimidone‐based bis‐heterocycles (Figure 4). The α‐cyanation of ethyl acetoacetate to produce the desired pyrazolones was ruled out because we anticipated chemoselectivity challenges when reacting the product with hydrazines. This provided us with an opportunity to explore our strategy for the preparation of bis‐heterocycles tethered with C1 and C2 alkyl linkers using bifunctional building blocks **29** (C1) and **30** (C2), which were produced via alkylations with bromoacetonitrile (C1) or acrylonitrile (C2), respectively. Gratifyingly, the reagents also aligned with our strategy to begin the syntheses using bulk fine chemicals,^[^ ^47^, ^48^, ^49^ ^]^ (e.g., acrylonitrile costs US$ 0.05 per g). C1 and C2 pyrazolones **31** and **32** were produced from phenyl hydrazine in good yields; however, only the C2 pyrimidone **33** could be accessed from benzamidine (Figure 4, left panel). In general, nitrile arming of the C1 and C2 linkers was significantly more challenging and generated more byproducts (Figure 4, left‐center panel). Thioamides (**34**, **37**, and **40**) were produced in modest yields for all three linker compounds; however, reaction times were generally longer than those of their aryl counterparts (for details, see Supporting Information). The formation of pyrazolone‐linker‐aminooximes (**35**, **38**, and **41**) was generally challenging due to slow single addition but fast double addition of hydroxylamine. While aminooxime **35** was completely intractable, **38** could be telescoped to afford a C2‐linked pyrimidone‐oxadiazole **45**, while pyrazolone‐oxadiazole **41** could be isolated in a poor yield. LiHMDS was an incompatible reagent with the linker series, so Pinner reactions using acetyl chloride in ethanol and ammonia were attempted to produce amidines (for further details, see Supporting Information). While effective for the C2 linkers **39** and **42**, the C1 pyrazolone **36** was recalcitrant, and various conditions formed an undesired ester byproduct.^[^ ^50^ ^]^ The formation of linked thiazoles (**43**, **44**, and **51**) proceeded smoothly by conversion to the vinamidinium‐based set in a similar manner (i.e., Figure 3), although the β‐ketoester C2 series provided significantly better isolated yields compared to C1 (**44** and **51**, versus **43**, 71%–75% versus 24%, Figure 4 right‐center panel). All linked tetrazoles **52**–**54** were produced from the parent nitriles (i.e., **31**–**33**) in good yields. For the C2 linkers, pyrimidine formation to yield **46** and **49** was sluggish. Bis‐pyrimidone formation performed significantly better than the pyrazolone series (**47** versus **50**, 59% versus 14%). In DMSO‐*d*6, the C2‐linked bis‐pyrimidone **47** exclusively adopts the mixed Ph(keto)‐Me(enol) tautomer as shown.

### Tris‐Heterocycles

**Figure 5:**
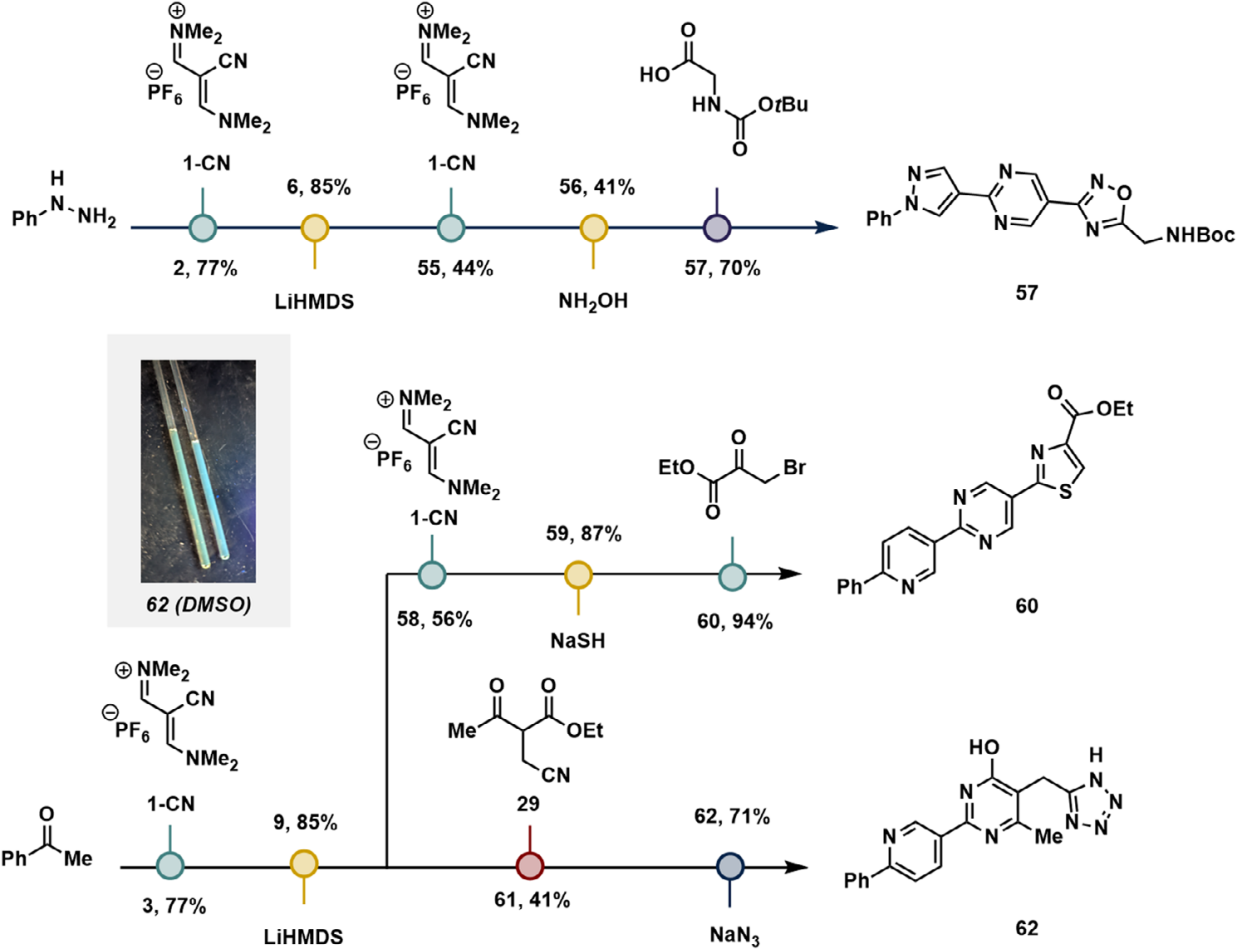
Iterative synthesis of tris‐heterocycles. Water was used to precipitate and purify the products of synthesis. NMR samples of **62** in DMSO‐d6 that fluoresce under long‐wave UV.

Pharmaceuticals, agrochemicals, and materials often contain extended (hetero)aromatic motifs to elicit specific properties such as target binding and light emission. To expand the capabilities of our strategy and venture toward a general oligoheterocycle synthesis platform, we sought to prepare extended tris‐heterocyclic compounds (Figure 5). Sequences could be varied accordingly to prepare three different combinations of tris‐heterocycles from seven different inputs.

Using the above platform, pyrazole **2** and pyridine **3** were prepared from phenyl hydrazine and acetophenone, respectively, then armed using LiHMDS to yield amidines **6** and **9**. From amidine **6**, cross‐condensation with vinamidinium salt **1‐CN** followed by the oxadiazole arming/condensation sequence afforded the pyrazole‐pyrimidine‐oxadiazole **57**. The amidine **9** could either be subjected to a cross‐condensation with vinamidinium salt **1‐CN** to afford the pyrazole‐pyridine nitrile **58**, followed by the thiazole arming/condensation sequence to afford the pyridine‐pyrimidine‐thiazole **60**, or cross‐condensation with β‐ketoester **29** followed by treatment with sodium azide to afford the pyridine‐pyridone‐C1‐tetrazole **62**, for subsequent arming steps to access the desired tris‐heterocycles. We have found that compound **62** fluoresced under long‐wave UV irradiation in DMSO. With five different starting materials offering access to two different heterocycles containing a nitrile linchpin for iterations, then capping with a possible five heterocycles, approximately 50 different heterocycle combinations are theoretically possible using the building blocks shown. We surmise that this initial demonstration sets a precedent for an iterative platform to produce highly varied oligoheterocycles.

### Peptide Hybrids

**Figure 6:**
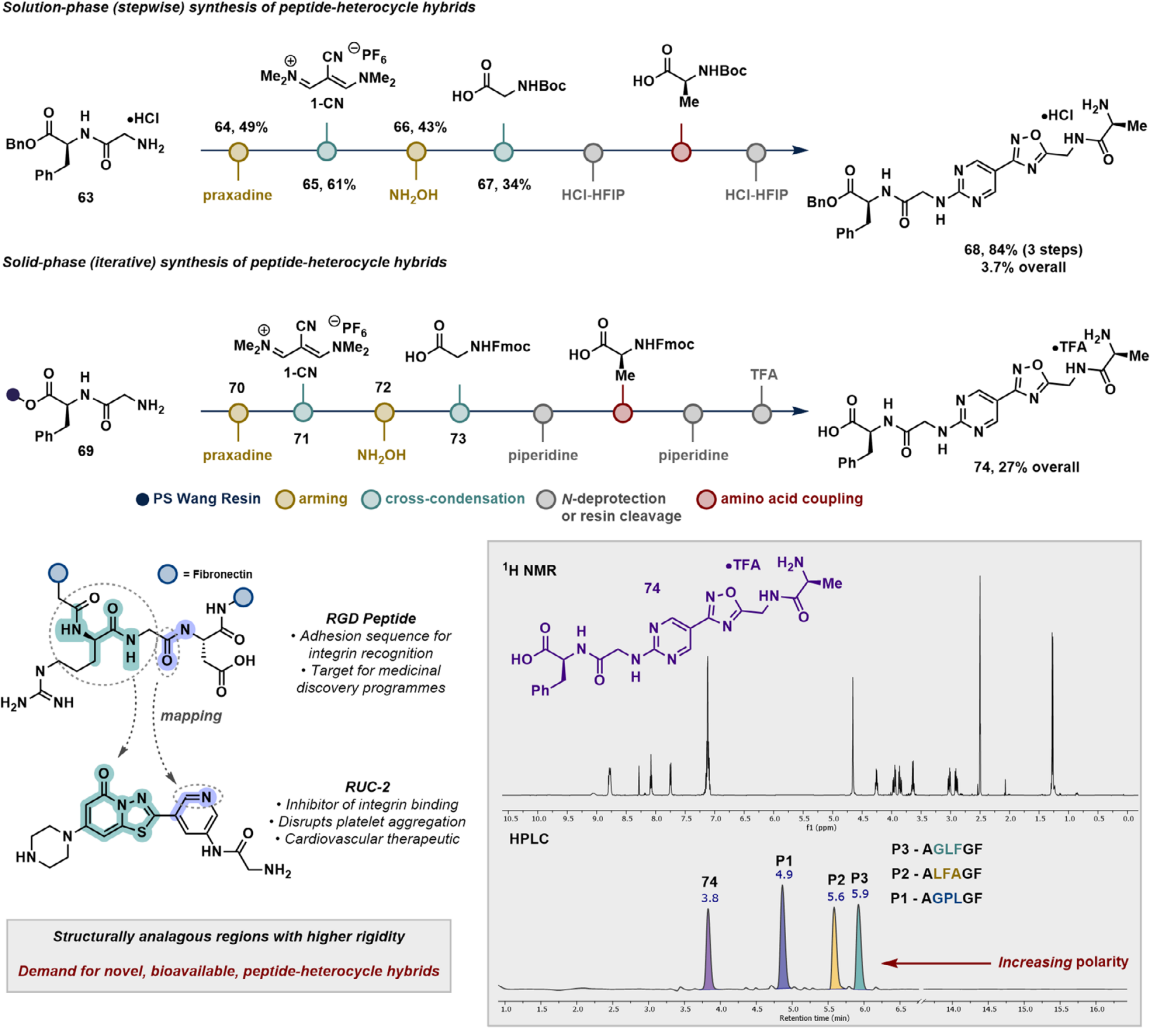
Integration of the frame‐shifted approach into peptide synthesis workflow. (Top) combination of cross‐condensation with liquid‐ and solid‐phase peptide synthesis. (Bottom left) RUC‐2 mapping onto the RGD fragment of fibronectin. (Bottom right) ^1^H NMR data of 74 and HPLC UV chromatogram (214 nm) showing the increased polarity of 74 compared to hexameric peptides (AGLFGF, ALFAGF, AGPLGF).

Typical iterative technologies use bifunctional building blocks to assemble complex molecules. For instance, amino acids can be combined to elongate polypeptide chains, while various platforms using borylated building blocks have been used to elongate chains of polyenes, polyarenes, and alkanes.^[^ ^51^, ^52^, ^53^, ^54^, ^55^ ^]^ A longstanding goal is to create iterative platforms with high degrees of molecular diversity; e.g., combining the cross‐coupling of heterocycles with polyenes within a single platform. We realized that our frame‐shifted strategy could be compatible with solid‐phase peptide synthesis to generate hybrid heterocycle‐containing peptides on a single platform that exclusively uses condensation‐driven processes. Initial investigations began in the solution phase from protected Gly‐Phe **63** (Figure 6). As a complementary mild method compared to our armings of nitriles to amidines, the Gly *N*‐terminus could be armed for cross‐condensation using praxadine as an operationally simple guanidinylating reagent.^[^ ^56^ ^]^ Cross‐condensation with **1‐CN** followed by oxadiazole formation proceeded in a similar fashion compared to our initial investigations. The practical utility of the installed aminomethylene fragment onto oxadiazoles was demonstrated via standard Boc deprotection and coupling of Ala, affording the benzyl ester‐protected peptide hybrid **68**.

We then sought to demonstrate some of the capabilities of cross‐condensation on a solid‐phase platform. Resin selection was critical to our strategy because several arming and heterocycle‐forming reactions required elevated temperatures. A 2‐chlorotrityl resin was ineffective at elevated temperatures and resulted in unwanted cleavage (see Supporting Information for further details); however, Wang resin could tolerate heating, and installation of Gly‐Phe was performed using conventional Fmoc SPPS (**69**). We then migrated to our cross‐condensation strategy and monitored each step by microcleavage/LCMS analysis. Arming with praxadine and cross‐condensation with **1‐CN** proceeded smoothly. The pyrimidine nitrile of **71** was then armed using hydroxylamine hydrochloride using standard conditions, but this step was ineffective due to poor swelling of the resin in ethanol. A solvent switch to DMF and elevating the temperature to 70 °C for 3 h provided **72** without unwanted cleavage. Oxadiazole synthesis to form **73** on resin was difficult at the required elevated temperature. Instead, the aminooxime was acylated with Fmoc‐Gly at room temperature prior to annulation with TBAF in THF at room temperature over 2 h. The Fmoc protecting group was only partially removed by this method; full deprotection was accomplished with piperidine. Final coupling of Ala, Fmoc deprotection, and cleavage from Wang resin afforded the deprotected hybrid adduct **74** as the TFA salt in 27% overall yield. We noted that, in comparison to three different hexameric sequences containing the same amino acid terminus (AGPLGF, ALFAGF, and AGLFGF), our hybrid molecule **74** exhibited a significantly shortened retention time (*t_R_ = *3.8 min) on reverse‐phase HPLC. This has provided an early indication that replacing residues with heterocycles can disrupt peptide polarity, likely by affecting the dipole moments, producing promising candidates for targeting extracellular PPIs.^[^ ^57^, ^58^ ^]^

Despite not being explicitly designed as peptidomimetics, many bioactive small molecules exhibit emergent structural features that recapitulate key elements of peptide recognition (e.g., RUC‐2 resembling RGD,^[^ ^59^ ^]^ Figure 6)—such as aligned dipoles, directional hydrogen bonds, and discrete stereoelectronic arrangements.^[^ ^60^, ^61^, ^62^, ^63^, ^64^ ^]^ This phenomenon, often revealed during high‐throughput screening or phenotypic discovery, underscores the capacity of rigid heterocyclic scaffolds to engage protein surfaces in ways that mimic natural peptides. In this light, oligoheterocycles—particularly those incorporating thiazoles, oxazoles, and related motifs—offer a compelling platform for functional mimicry.^[^ ^65^, ^66^ ^]^ Their conformational rigidity, capacity for predictable noncovalent interactions, and modular synthesis render aromatic heterocycles ideal candidates for emulating the topological and electrostatic patterns found in peptide–protein interfaces.^[^ ^57^, ^58^, ^67^, ^68^ ^]^ The iterative construction of such motifs thus represents not only a synthetic opportunity but a conceptual bridge between small‐molecule chemistry and the logic of protein recognition.

### Scaffold Novelty

**Figure 7:**
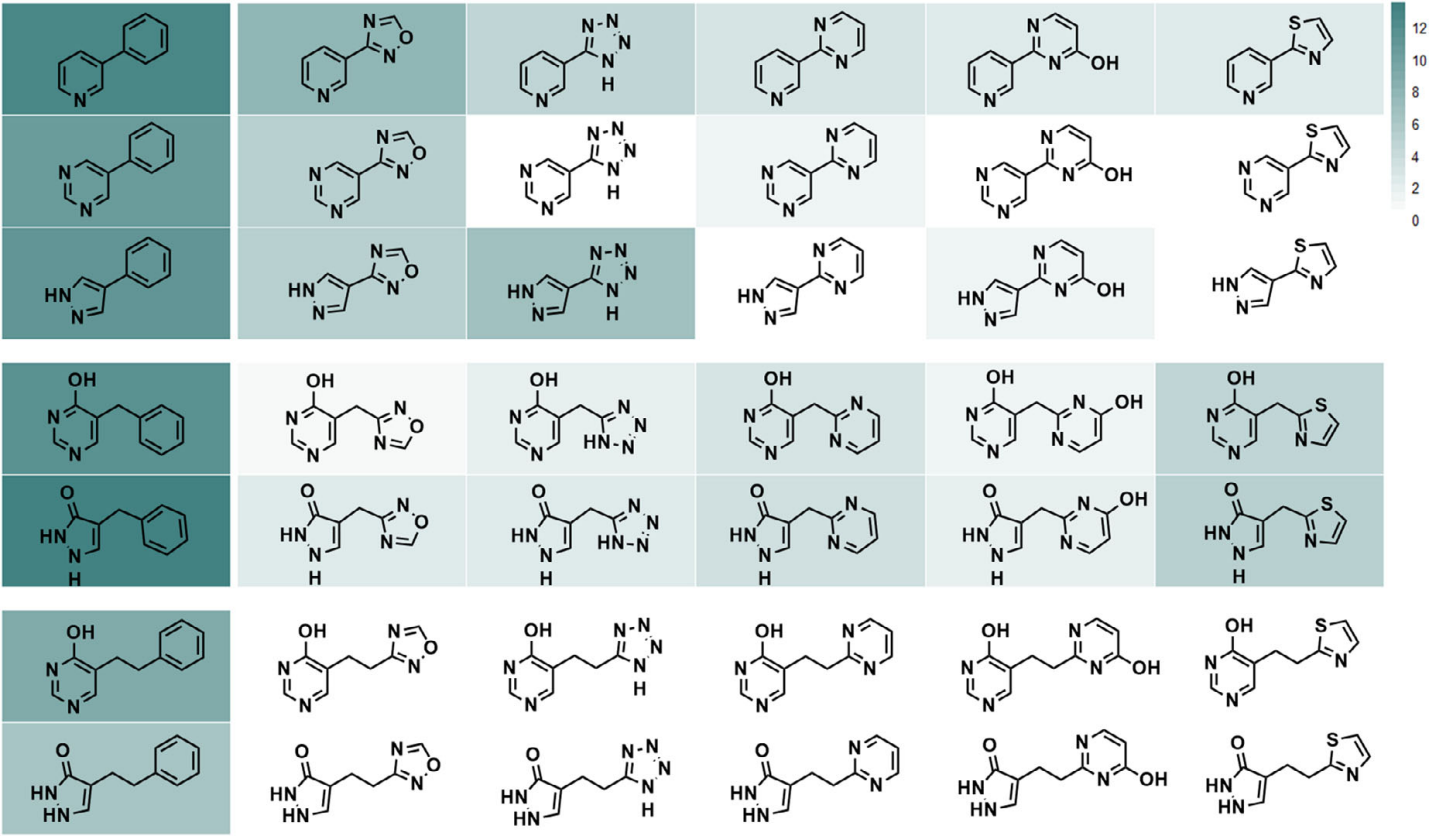
Heat map (log scale) of SciFinder search (performed December 2024) results for different scaffolds. Darkest (most hits, millions) to white (zero).

In total, 27 different bis‐heterocyclic scaffolds have been accessed using our frame‐shifted strategy. We performed a literature search to examine how these scaffolds might be synthesized. To our surprise, many combinations of bis‐heterocycles were completely unreported (Figure 7), in stark contrast to benchmark searches of phenylated heterocycles (literature publications): pyridine (>258 000), pyrimidine (>38 000), and pyrazole (>5000). Pyridine‐oxadiazole and pyrazole‐tetrazole were the two most common scaffolds of bis‐heterocycles, with 2667 and 774 occurrences, respectively. The remainder of the bis(heteroaryl) series had less than 160 results, with some having zero hits for the regiochemistry shown, including pyrimidine‐tetrazole, pyrimidine‐pyrimidine, pyrimidine‐thiazole, pyrazole‐pyrimidine, and pyrazole‐thiazole. Searches for scaffolds with a methylene spacer displayed similar rarity with < 140 occurrences, yet all in this series were reported. Moreover, bis‐heterocycles with an ethylene spacer were completely unreported. We account for this contrast by the ongoing bias for metal‐mediated cross‐coupling strategies and inherent challenges that arise from competing side reactions such as β‐hydride elimination.^[^ ^9^, ^10^, ^69^ ^]^

## Conclusion

This work represents a strategic “frame shift” in the retrosynthetic analysis of oligoheterocycles, centered on disconnections that exploit carbon atoms of high‐oxidation states. The result is a modular strategy for synthesizing oligoheterocycles using bifunctional building blocks capable of chemoselective cross‐condensation and nitrile arming. This approach bypasses traditional cross‐coupling methods, granting access to both established and novel scaffolds that are often difficult or expensive to procure using conventional retrosynthetic logic.

The ability to access diverse heterocycle‐heterocycle and heterocycle‐linker‐heterocycle motifs positions this chemistry as a tool for applications across medicinal, agrochemical, and materials science. More broadly, our results underscore the power of sustainable design principles in heterocycle discovery: by repurposing established chemical reactions and abundant feedstocks, we can accelerate the synthesis of novel heterocyclic scaffolds with potential for downstream functionalization and discovery. In our approach, skeletal diversity is generated not by sourcing different heterocycle partners, but by employing linker‐centered building blocks focused on higher oxidation states of carbon. Given the facility with which nitrile functionality can be armed to deliver varied heterocycle combinations, we anticipate that many common functional groups could be similarly armed with the frame‐shift objective in mind, opening new space for heterocycle design. Our method should be adaptable to solid‐phase peptide synthesis, and the iterative nature of our strategy should be appealing to automated synthesis platforms. The successful implementation of cross‐condensation on solid support highlights the potential of this method for rapid library construction. Furthermore, the programmable nature of our strategy allows the integration of heterocycles, linkers, and peptides into hybrid bioactive architectures.

While the present study establishes the feasibility of frame‐shifted synthesis, several important challenges need to be addressed in the future. First, new arming strategies are needed to fully exploit the concept. The nitrile group has proven effective, enabling heterocycle‐forming condensations and aryl nitrile arming steps under neutral or basic conditions, but its scope is uneven across different heterocycle/linker combinations. Alkyl nitriles, thioamides, and amidoximes can be obtained under mild neutral or basic conditions, whereas amidines typically require the strongly acidic Pinner reaction. The development of arming groups compatible with a single, unified set of conditions will be essential to realizing the full potential of this technology. Second, unlike conventional iterative synthesis based on cross‐coupling, where a limited set of reactions can use diverse building blocks, the frame‐shifted approach demands distinct modules for each new heterocycle. Although this introduces additional synthetic demand, it also creates an opportunity for *chemodivergence*: from a single module, multiple frameworks can be accessed through selective condensation pathways. This outcome is unattainable in traditional cross‐coupling‐based strategies.^[^ ^70^ ^]^

## Supporting Information

The authors have cited additional references within the Supporting Information.^[^ ^71^, ^72^, ^73^, ^74^, ^75^ ^]^

## Author Contributions

A.K.Y. conceived the concept of frame‐shifted synthesis; experiments were designed by C.E.G., M.J.C., and K.A.C.B.; synthesis of bis‐heterocycles and tris‐heterocycles was carried out by C.E.G., K.A.C.B., M.J.C., and B.Z.H.; hybrid peptide synthesis was performed by C.E.G. and Y.D.O. The project was supervised by C.E.G., K.A.C.B., and A.K.Y.; C.E.G., K.A.C.B., and A.K.Y. wrote the paper.

## Conflict of Interests

The authors declare no conflict of interest.

## Supporting information



Supporting Information

## Data Availability

The data that support the findings of this study are available from the corresponding author upon reasonable request.
