# Supplementary material for: Frame‐Shifted Synthesis of Oligoheterocycles as a Platform for Molecular Design
Source: Angew Chem Int Ed Engl. 2025 Nov 17;65(2):e20677. doi: 10.1002/anie.202520677 (PMC12790365; doi:10.1002/anie.202520677)
Supplement: Supplementary file 1 — Supporting Information [file ANIE-65-e20677-s001.docx]

**Frame-shifted synthesis of oligoheterocycles as a platform for molecular design**

Kane A.C. Bastick^‡^, Caleb E. Griesbach^‡^, Ben Zhen Huang, Morgan J. Cordell, Yang (Daniel) Ou, Alán Aspuru-Guzik, Andrei K. Yudin*

Supporting Information

**Table of Contents**

[**1. General** 3](#_Toc205297549)

[**2. Experimental** 4](#_Toc205297550)

[**2.1 General synthetic procedures** 4](#_Toc205297551)

[**2.2 Characterization of compounds** 7](#_Toc205297552)

[**2.3 Controls for the stability of 2-Cl-Trt resin** 38](#_Toc205297553)

[**2.4 Solid-phase peptide synthesis** 38](#_Toc205297554)

[**3. Copies of spectra for all novel compounds** 42](#_Toc205297555)

[**4. X-ray crystallography** 124](#_Toc205297556)

[**5. Stacked LC report of 74 with hexameric peptides** 137](#_Toc205297557)

[**6. References** 146](#_Toc205297558)

# **1. General**

All reagents were used as received from commercial sources (Sigma Aldrich, Alfa Aesar, Combiblock, or Oakwood) unless otherwise noted. Bottle EtOH (absolute), *n*-butanol, and DMF were dried over 4 A molecular sieves and stored accordingly. LiHMDS (Sigma Aldrich) was purchased as a ready-made solution under a sure-seal and used as received. All other solvents and reagents were used as received.

**Chromatography**: Thin-layer chromatography (TLC) was performed using Merck silica gel 60 F 254 plates, with UV (254 nm) detection followed by KMnO_4_ stain as required. Santai Technologies pre-packed silica gel columns (Sepa high-efficiency silica gel 20- 45 µm, 4 g, 12 g, 30 g, 60 g or 120 g cartridge as required) were used for compounds purified by flash normal-phase automated flash-column chromatography eluting with various two-combinations of hexanes/DCM/EtOAc/*i*PrOH. Santai Technologies pre-packed C18 columns (spherical C18, 20-45 µm, 12 g, 30 g, or 120 g cartridge as required) were used for compounds that were purified by flash reversed-phase chromatography, eluting with MeCN/water buffered with 0.1% formic acid.

**NMR Spectrometry:** All NMR spectra were recorded on either a Bruker DPX300, a Bruker AV 300, a Bruker AV 400 at 300 K, an Agilent 500 MHz DD2 NMR Spectrometer at 298 K or a Varian 600 Unity spectrometer at 298 K, unless otherwise stated. ^1^H NMR spectra chemical shifts (δ) were reported in parts per million (ppm) referenced to residual protonated solvent peak (CDCl_3_ δ = 7.26 ppm; CD_3_CN δ = 2.13 ppm; CD_3_OD δ = 4.87 ppm; DMF-*d*7 δ = 2.75 ppm; DMSO-*d*6 δ = 2.50 ppm; THF-*d*8 δ = 1.73 ppm). Depending on the commercial availability of deuterated solvents, some contained TMS while other batches did not; regardless, all chemical shifts were referenced to the previous solvent peaks and all NMR machines were internally calibrated. Spectral data is reported as follows: chemical shift, multiplicity (s = singlet, d = doublet, t = triplet, q = quartet, quin. = quintet, sext. = sextet, hept. = heptet, multiplets thereof, or m = multiplet, or br = broad singlet), coupling constant (J) in Hertz (Hz), then integration. All couplings reported refer to ^3^J_HH_, unless otherwise stated. ^13^C NMR spectra chemical shifts (δ) were reported in parts per million (ppm) and were referenced to carbon resonances in the NMR solvent (CDCl_3_ δ = 77.2 ppm; CD_3_CN δ = 1.3 ppm; CD_3_OD δ = 49.0 ppm; DMF-*d*7 δ = 29.8 ppm; DMSO-*d*6 δ = 39.5 ppm; THF-*d*8 δ = 25.4 ppm).

**Mass Spectrometry:** High-resolution mass spectrometry (HRMS) ESI (m/z) spectra were recorded on a Bruker MicroTof or an Orbitrap LTQ XL (Nanospray) of Thermo Scientific. At the University of Toronto high resolution mass spectra were obtained on a VG 70-250S (double focusing) mass spectrometer at 70 eV or on an ABI/Sciex Qstar mass spectrometer with ESI source, MS/MS and accurate mass capabilities or on JEOL AccuTOF-DART instrument. RP-HPLC/MS: Low-resolution mass spectra (ESI) were collected on an Agilent Technologies 1200 series HPLC paired to a 6130 Mass Spectrometer. Compounds were resolved on Phenomenex’s Kinetex 2.6m C18 50x4.6mm column at room temperature with a flow of 1 mL/min. The gradient consisted of eluents A (0.1% formic acid in double distilled water) and B (0.1% formic acid in HPLC-grade acetonitrile). LCMS method: A linear gradient starting from 5% of B to 95% over 15 min or 6 min at a flow rate of 1.0 mL/min.

# **2. Experimental**

## **2.1 General synthetic procedures**

**General Procedure A – Functionalised heterocycle formation from vanimindinium salts**

A sample vial fitted with a stir bar was charged with the vanimidinium salt (1.0 eq.) and DMF (1.35 mL). While stirring at rt, the coupling partner (*e.g.*, amidine, hydrazine, 1.0 eq.) was added in one portion, followed by *N*-methylmorpholine (2.5 eq.). The reaction mixture was heated as specified for 3 h, unless stated otherwise. Unless stated otherwise, the desired product was obtained by precipitation from water and drying the solid under vacuum.

**General Procedure B– arming of nitriles to amino oximes**

A sample vial fitted with a stir bar was charged with the nitrile (1 eq.). and hydroxylamine hydrochloride (1.5 eq.), which were dissolved in ethanol (0.4 M). Triethylamine (1.5 eq.) was added at rt, and the solution was allowed to stir for 1 h at rt, unless otherwise stated. The desired product was purified as stated.

**General Procedure C – arming of heteroaromatic nitriles to amidines**

To an oven-dried vial fitted with a stir bar, the nitrile (1 eq.) was added. Then, LiHMDS (1 M in THF, 2 eq.) was added to the vial while rapidly stirring at rt* and the dark suspension was allowed to stir for 1 h, unless otherwise stated. The vial was opened and the reaction was quenched by the dropwise addition of HCl (1 M in MeOH, 2 eq*., gas evolution*) and the contents were concentrated. The crude material was purified by reverse phase chromatography to yield the amidine, unless otherwise stated.

** Practical note: the vial did not require an atmosphere of N_2_ but the bottle of LiHMDS used was sealed under N_2_.*

**General Procedure D – arming of nitriles to thioamides**

To a sample vial fitted with a stir bar, the nitrile (1 eq.) sodium hydrosulfide monohydrate (1.75 eq.), and anhydrous magnesium chloride (1 eq.) were added. The solids were dissolved in DMF (0.33 M, *immediately turns blue-green and generates the smell of rotten eggs*). The suspension was then heated to 60 °C while rapidly stirring for 1 h, unless otherwise stated. The reaction mixture was concentrated at reduced pressure, then purified as stated.

**General Procedure E – oxadiazole formation from amino oximes**

To a sample vial fitted with a stir bar, Boc-Gly-OH (1 eq.) and HCTU (1.1 eq.) were added. The solids were suspended in DMF (0.2 M), then DIPEA (2 eq.) was added in one portion while stirring at rt. After 5 min., the amino oxime (1 eq.) was added in one portion and the reaction mixture was stirred for 1 h, unless otherwise stated.* The reaction mixture was heated to 110 °C and stirred for 1 h, unless otherwise stated, then cooled to rt and water was added. The solids were filtered off (keep solids) and washed further with water (3x). Further purification was performed as required.

**Practical note: The reaction mixture was monitored by LCMS to check for initial acid-oxime coupling. The reaction was only warmed to 110 °C once all the starting material had been consumed to reduce byproduct formation.*

**General Procedure F – pyrimidone formation from amidines and β-ketoesters**

To a sample vial fitted with a stir bar, the amidine (1.0 eq.) was added, followed by MeCN (0.20 M). While stirring at rt, the β-ketoester (*i.e.*, ethyl 3-oxobutanoate, 1.0 eq) was added in one portion, followed by K_2_CO_3_ (1.5 eq.). The resulting solution was allowed to stir overnight (14–18 h) at 70 °C, unless otherwise stated. The reaction mixture was cooled to rt and the pyrimidone product purified as stated.

**General Procedure G – thiazole formation from thioamides and α-bromoethylpyruvate**

To a sample vial fitted with a stir bar, the thioamide (1.0 eq.) was added, followed by EtOH (0.24 M). While stirring at rt, ethyl bromopyruvate (1.2 eq) was added in one portion and resulting solution was allowed to stir for 1 h at 70 °C, unless otherwise stated. The reaction mixture was cooled to rt and the thiazole product purified as stated.

**General Procedure H – tetrazole formation from nitriles**

A sample vial fitted with a stir bar was charged with the solids; the nitrile (1.0 eq.), sodium azide (1.1 eq.), and ammonium chloride (2.0 eq.). The solids were suspended in DMF (1.0 M) then heated to 110 °C and stirred for 1 h, unless otherwise stated.* The tetrazole product was purified by reverse phase C18 flash column chromatography 0–100% MeCN in H_2_O.**

** Practical note: at the scales performed in this study, a bomb screen was not necessary. The procedure should be adjusted when significantly raising the scale.*

*** Safety note: the first fraction should be handled with extra caution due to the small excess of NaN_3_ being exposed to mobile phase containing 0.1% formic acid.*

## **2.2 Characterization of compounds**

**(*Z*)-*N*-(2-cyano-3-(dimethylamino)allylidene)-*N*-methylmethanaminium hexafluorophosphate(V), 1-CN.**

To a 250 mL round-bottom flask fitted with a stir bar, DMF (15.0 mL) was added and POCl_3_ (15.0 mL, 160 mmol, 17.4 eq.) was added dropwise at 0 °C. The solution was warmed to rt over 30 min. and DCM (23.0 mL) was added before being cooled to 0 °C. Then *trans*-3-dimethylaminoacrylonitrile* (881 mg, 9.17 mmol, 1.00 eq.) as a solution in DCM (120 mL) was added in portions. The resulting solution was again allowed to warm to room temperature and the DCM was removed under reduced pressure. A solution of NaPF_6_ (1.70 g, 10.1 mmol, 1.10 eq.) in water (30.0 mL) was added to the crude mixture, resulting in a precipitate that was filtered and recrystallized from EtOH to provide a yellow solid (703 mg, 23%)

**^1^H NMR (500 MHz, MeCN-*d3*)** δ 7.57 – 7.46 (m, 2H), 3.49 (d, *J* = 0.8 Hz, 6H), 3.34 (d, *J* = 0.7 Hz, 6H).

**^13^C NMR (126 MHz, MeCN-*d3*)** δ 165.5, 116.2, 74.2, 50.1, 40.4.

**HRMS (ESI+)** Exact mass calcd. for C_8_H_14_N_3_ [M+]^+^ = 152.1182; found 152.1184.

^^* *trans*-3-dimethylaminoacrylonitrile was synthesized from cyanoacetic acid according to patent US 20130210844 A1 (see step 2: preparation of ethyl 5-cyano-2-(difluoromethyl)nicotinate ##STR00157##) in comparable scales and yields.

**(*Z*)-*N*-(2-chloro-3-(dimethylamino)allylidene)-*N*-methylmethanaminium hexafluorophosphate(V), 1-Cl.**

Batches of compound **1-Cl** was prepared according to Reider and coworkers (*see compound* ***3a*** *in the manuscript*) in comparable scales and yields.^[1]^

**1-phenyl-1H-pyrazole-4-carbonitrile, 2.**

Prepared according to General Procedure **A** on a 1.0 mmol scale using compound **1-CN** (290 mg, 1.0 mmol, 1.0 eq.), DMF (1.35 mL), phenyl hydrazine (0.10 mL, 1.0 mmol, 1.0 eq.), and *N*-methylmorpholine (0.28 mL, 2.5 mmol, 2.5 eq.) at 110 °C for 3 h, cooled to rt, then 5 mL water was added. The solids were filtered off (keep solids), and washed with water (3x). After precipitation from water, the solid was dissolved in 10 mL EtOAc and any remaining insoluble salts filtered off. The liquor was concentrated at reduced pressure to afford the desired product as an off-white solid (130 mg, 77%).

The above procedure was also performed at 0.1 mmol scale (4.4 mg, 26%).

**^1^H NMR (500 MHz, CDCl_3_)** δ 8.31 (d, *J* = 0.7 Hz, 1H), 8.00 (d, *J* = 0.6 Hz, 1H), 7.73 – 7.64 (m, 2H), 7.57 – 7.47 (m, 2H), 7.46 – 7.37 (m, 1H).

**^13^C NMR (126 MHz, CDCl_3_)** δ 143.4, 138.9, 131.9, 123.0, 128.5, 120.1, 113.2, 94.5.

The spectral data were consistent with the literature.^[2]^

**6-phenylnicotinonitrile, 3.**

To an oven-dried microwave vial fitted with a stir bar and cooled under N_2_, THF (2.5 mL) and acetophenone (0.12 mL, 1.0 mmol, 1.0 eq.) were added in one portion. The solution was cooled to −78 °C (dry ice / acetone) then the septum was removed and potassium *tert*-butoxide (340 mg, 3.0 mmol, 3.0 eq.) was added portionwise, then the septum was returned. The bright red solution was stirred at −78 °C for 10 min., then compound **1-CN** (343 mg, 1.2 mmol, 1.2 eq.) was added in one portion. After 1 h at −78 °C, the cooling bath was removed and the reaction mixture was warmed to rt. The septum was removed and ammonium acetate (385 mg, 5.0 mmol, 5.0 eq.) and *n*BuOH (0.40 mL) were added sequentially in one portion. The temperature was initially raised to 70 °C with the septum removed (*evaporates THF in under 3 h*), then the septum returned, and the reaction mixture stirred overnight at 110 °C. The neat reaction mixture was subject to flash column chromatography on silica gel (0–30% EtOAc in hexanes) to afford the desired product as an off-white solid (80 mg, 44%).

**^1^H NMR (500 MHz, CDCl_3_)** δ 8.95 (dd, *J* = 2.2, 0.9 Hz, 1H), 8.11 – 8.03 (m, 2H), 8.01 (dd, *J* = 8.3, 2.2 Hz, 1H), 7.85 (dd, *J* = 8.3, 0.9 Hz, 1H), 7.58 – 7.45 (m, 3H).

**^13^C NMR (126 MHz, CDCl_3_)** δ 160.7, 152.6, 140.0, 137.5, 130.8, 129.2, 127.5, 120.1, 117.1, 108.0.

The spectral data were consistent with the literature.^[3]^

**2-phenylpyrimidine-5-carbonitrile, 4.**

Prepared according to General Procedure **A** on a 2.0 mmol scale using **1-CN** (654 mg, 2.20 mmol, 1.10 eq.), DMF (2.70 mL), benzamidine hydrochloride (313 mg, 2.00 mmol, 1.00 eq.), and *N*-methylmorpholine (0.55 mL, 5.0 mmol, 2.5 eq.) at 70 °C for 3 h. The reaction mixture was cooled to rt, then 5.0 mL water was added. The solids were filtered off (keep solids) and washed with water (3x). Further drying in a desiccator afforded the desired product as a white solid (265.8 mg, 73%). No further purification was required.

**^1^H NMR (400 MHz, DMSO-*d*_6_)** δ 9.38 (s, 2H), 8.48 – 8.41 (m, 2H), 7.68 – 7.55 (m, 3H).

**^13^C NMR (100 MHz, DMSO-*d*_6_)** δ 164.6, 160.9, 135.6, 132.5, 129.1, 128.6, 115.6, 106.8.

**HRMS (DART+)** Exact mass calcd. for C_11_H_8_N_3_ [M+H]^+^ = 182.07127; found 182.07221.

***N*'-hydroxy-1-phenyl-1H-pyrazole-4-carboximidamide, 5.**

Prepared according to General Procedure B on a 1.0 mmol scale using compound **2** (170 mg, 1.0 mmol, 1.0 eq.), hydroxylamine hydrochloride (104 mg, 1.5 mmol, 1.0 eq.) and Et_3_N (0.21 mL, 1.5 mmol, 1.5 eq.) in EtOH (2.5 mL, 0.4 M). After 1 h the desired product precipitated from the reaction mixture. ~2 mL solvent was removed by pipette, then the reaction mixture concentrated at reduced pressure. The desired product was obtained following purification by C18 flash column chromatography (0–100% MeCN in H_2_O) as a beige solid (95.1 mg, 47%).

*Characterization reflects the major isomer. The compound was stabilized in solution by the addition of 1 eq. EtOH.*

**^1^H NMR (500 MHz DMSO-*d*_6_)** δ 8.73 (s, 1H), 7.92 (s, 1H), 7.82 – 7.73 (m, 2H), 7.54 – 7.49 (m, 2H), 7.35 – 7.29 (m, 1H), 5.77 (s, 2H).

**^13^C NMR (126 MHz, DMSO-*d*_6_)** δ 163.1, 145.6, 139.4, 138.5, 129.6, 126.4, 125.3, 118.3.

**HRMS (ESI+)** Exact mass calcd. for C_10_H_11_N_4_O [M+H]^+^ = 203.09274; found 203.09214.

**1-phenyl-1H-pyrazole-4-carboximidamide, 6.**

Prepared according to General Procedure **C** on a 1.0 mmol scale using compound **2 (**170 mg, 1.0 mmol, 1.0 eq.) and 1 M LiHMDS in THF (2.0 mL, 2.0 eq.), then 1N HCl in MeOH (2.0 mL, 2.0 eq.). After quenching with HCl, the reaction mixture was concentrated at reduced pressure, then the desired product was precipitated from H_2_O. The product was filtered off, washed with H_2_O (3x), and dried to afford the desired product as an off-white solid (75 mg, 41%).

**^1^H NMR (500 MHz, CDCl_3_)** δ 8.52 – 8.31 (m, 1H), 7.99 – 7.86 (m, 1H), 7.75 – 7.66 (m, 2H), 7.53 – 7.46 (m, 2H), 7.39 – 7.33 (m, 1H).

**^13^C NMR (126 MHz, CDCl_3_)** δ 163.8, 139.6, 129.6, 129.0, 127.6, 119.6, 119.4, 119.2.

**HRMS (ESI+)** Exact mass calcd. for C_10_H_11_N_4_ [M+H]^+^ = 187.09782; found 187.09685.

**1-phenyl-1H-pyrazole-4-carbothioamide, 7.**

Prepared according to General Procedure **D** on a 1.0 mmol scale using compound 2 (170 mg, 1.0 mmol, 1.0 eq.), sodium hydrogen sulfide monohydrate (150 mg, 1.75 mmol, 1.75 eq.) and magnesium chloride (190 mg, 1.0 mmol, 1.0 eq.) in DMF (3 mL). The reaction was complete after 1 h at 60 °C, and the desired product was obtained following precipitation with H_2_O as a pale yellow solid (42 mg, 21%).

**^1^H NMR (500 MHz, CDCl_3_)** δ 8.56 (d, *J* = 0.7 Hz, 1H), 7.96 (d, *J* = 0.7 Hz, 1H), 7.76 – 7.66 (m, 2H), 7.52 – 7.44 (m, 2H), 7.37 (dt, *J* = 7.9, 1.4 Hz, 1H).

**^13^C NMR (126 MHz, CDCl_3_)** δ 192.6, 139.3, 137.5, 130.7, 129.8, 127.9, 125.7, 119.8.

**HRMS (ESI+)** Exact mass calcd. for C_10_H_10_N_3_S^32^ [M+H]^+^ = 204.05881; found 204.05871.

***N*'-hydroxy-6-phenylnicotinimidamide, 8.**

Prepared according to General Procedure **B** on a 1.0 mmol scale using compound **3** (180 mg, 1.0 mmol, 1.0 eq.), hydroxylamine hydrochloride (103 mg, 1.5 mmol, 1.5 eq.), and Et_3_N (0.21 mL, 1.5 mmol, 1.5 eq.) in EtOH (2.5 mL). After 1 h at rt, the reaction was complete by LCMS and the desired product was precipitated from H_2_O, filtered off, and dried to afford the desired product as a pale yellow solid (201 mg, 41%).*

**Practical notes: repeated efforts of azeotroping/vacuum could not remove 6.0 equivalents of EtOH from the product, the yield calculation reflects this. Attempts to remove EtOH by heating under vacuum resulted in partial decomposition. The compound was carried through to the next step as a •6EtOH complex without further complications.*

**^1^H NMR (500 MHz, DMSO-*d*_6_)** δ 10.29 (brs, 1NH), 9.89 (brs, 1NH), 8.95 (dd, *J* = 2.3, 0.9 Hz, 1H), 8.11 (dt, *J* = 8.5, 2.2 Hz, 3H), 7.99 (dd, *J* = 8.4, 0.9 Hz, 1H), 7.56 – 7.40 (m, 3H), 6.04 (brs, 1OH), 3.05 (q, *J* = 7.3 Hz, EtOH), 1.20 (t, *J* = 7.3 Hz, EtOH).

**^13^C NMR (126 MHz, DMSO-*d*_6_)** δ 156.0, 148.8, 146.5, 138.1, 133.9, 129.3, 128.8, 127.6, 126.5, 119.5, 45.2 (EtOH), 8.4 (EtOH).

**HRMS (ESI+)** Exact mass calcd. for C_12_H_12_N_3_O [M+H]^+^ = 214.09749; found 214.09693.

**6-phenylnicotinimidamide, 9.**

Prepared according to General Procedure **C** on a 1.0 mmol scale using compound **3** (180.3 mg, 1.0 mmol, 1.0 eq.) and 1 M LiHMDS in THF (2.0 mL, 2.0 eq.), then 1N HCl in MeOH (2.0 mL, 2.0 eq.). After quenching with HCl, ~ 10 mL H2O was added. The product was filtered off and dried in a dessicator to afford the desired product as a yellow powder (165 mg, 84%).

*Synthesis note: The above batch could be used for onward manipulations despite containing unknown impurities detected on LCMS. The below procedure was applied to afford higher purity of compound* ***9****, isolated as the formic acid salt.*

Prepared according to General Procedure **C** on a 1.0 mmol scale using compound **3** (180.3 mg, 1.0 mmol, 1.0 eq.) and 1 M LiHMDS in THF (2.0 mL, 2.0 eq.), then 1N HCl in MeOH (2.0 mL, 2.0 eq.). After quenching with HCl, the reaction mixture was concentrated at reduced pressure, then the desired product purified via reverse-phase flash chromatography (10-90% MeCN in H_2_O) to afford the desired product as a white solid (176 mg, 72%).

**^1^H NMR (400 MHz, DMSO-*d6*)** δ 10.41 (s, 4NH), 9.06 (dd, *J* = 2.5, 0.9 Hz, 1H), 8.47 (s, 1H), 8.28 (dd, *J* = 8.4, 2.4 Hz, 1H), 8.24 – 8.17 (m, 3H), 7.60 – 7.50 (m, 3H).

**^13^C NMR (100 MHz, DMSO-*d6*)** δ 167.6, 164.2, 159.6, 148.5, 137.3, 136.8, 130.3, 129.0, 127.0, 124.2, 119.7.

**HRMS (ESI+)** Exact mass calcd. for C_12_H_12_N_3_ [M+H]^+^ = 198.10257; found 198.10200.

**6-phenylpyridine-3-carbothioamide, 10.**

Prepared according to **General Procedure D** on a 1.0 mmol scale using compound **3 (**180 mg, 1.0 mmol, 1.0 eq.), sodium hydrogen sulfide monohydrate (150 mg, 1.75 mmol, 1.75 eq.) and magnesium chloride (190 mg, 1.0 mmol, 1.0 eq.) in DMF (3 mL). The reaction was complete after 1 h at 60 °C, cooled to rt, then diluted in ~10 mL EtOAc. Insoluble salts were filtered off and washed with EtOAc (3x) and the kept liquor was concentrated at reduced pressure. The desired product was obtained following precipitation with H_2_O as a bright yellow solid (180 mg, 84%).

**^1^H NMR (500 MHz, CDCl_3_)** δ 9.10 (dd, *J* = 2.5, 0.8 Hz, 1H), 8.34 (dd, *J* = 8.4, 2.5 Hz, 1H), 8.08 – 7.99 (m, 2H), 7.80 (dd, *J* = 8.3, 0.8 Hz, 1H), 7.56 – 7.43 (m, 3H).

**^13^C NMR (126 MHz, DMSO-*d*_6_)** δ 197.3, 157.8, 147.9, 137.7, 136.1, 133.3, 129.8, 128.9, 126.9, 119.2.

**HRMS (ESI+)** Exact mass calcd. for C_12_H_11_N_2_S^32^ [M+H]^+^ = 215.06375; found 215.06404.

***N*'-hydroxy-2-phenylpyrimidine-5-carboximidamide, 11.**

 Prepared according to General Procedure **B** on a 0.2 mmol scale using compound **4** (36.4 mg, 0.20 mmol, 1.0 eq.), hydroxylamine hydrochloride (20.9 mg, 0.30 mmol, 1.5 eq.), and Et_3_N (42 µL, 0.30 mmol, 1.5 eq.) in EtOH (0.50 mL). After 1 h at rt, the reaction was completed by LCMS and the desired product was filtered off, washed with EtOH (2x), and dried to afford the desired product as a white solid (29.2 mg, 68%)

**^1^H NMR (500 MHz, DMSO-*d*_6_)** δ 10.07 (s, 1OH), 9.12 (s, 1H), 8.44 – 8.39 (m, 2H), 7.57 – 7.52 (m, 3H), 6.16 (s, 2NH).

**^13^C NMR (126 MHz, DMSO-*d*_6_)** δ 163.1, 154.2, 146.9, 136.7, 131.0, 128.8, 127.7, 124.9.

**HRMS (DART+)** Exact mass calcd. for C_11_H_10_N_4_O [M+H]^+^ = 215.09274; found 215.09398.

**2-phenylpyrimidine-5-carboximidamide formic acid salt, 12.***

Prepared according to General Procedure **C** on a 1.0 mmol scale using compound **4** (182 mg, 1.00 mmol, 1.0 eq.) and 1 M LiHMDS in THF (2.0 mL, 2.0 eq.), then 1N HCl in MeOH (2.00 mL, 2.0 eq.). After quenching with HCl, the reaction mixture was concentrated at reduced pressure, then the desired product purified via reverse-phase flash chromatography (10-90% MeCN in H_2_O) to afford the desired product as a white solid (186 mg, 76%).

**Synthesis note: The formate counterion originates from the use of 0.1% formic acid during reverse-phase chromatography. For details, see General.*

**^1^H NMR (500 MHz, DMSO-*d*_6_)** δ 9.26 (s, 2H), 8.48 – 8.45 (app. m, 4H), 7.65 – 7.55 (m, 3H).

**^13^C NMR (126 MHz, DMSO-*d*_6_)** δ 166.9, 165.6, 162.1, 156.9, 136.0, 132.0, 129.0, 128.3, 122.6.

**HRMS (DART+)** Exact mass calcd. for C_11_H_11_N_4_ [M+H]^+^ = 199.09782; found 199.09747.

**2-phenylpyrimidine-5-carbothioamide, 13.**

 Prepared according to General Procedure **D** on a 0.2 mmol scale using compound **4** (36.4 mg, 0.20 mmol, 1.0 eq.), sodium hydrogen sulfide monohydrate (26.5 mg, 0.358 mmol, 1.75 eq.) and magnesium chloride (19.5 mg, 0.20 mmol, 1.0 eq.) in DMF (0.60 mL). The reaction was complete after 1 h at 60 °C, cooled to rt, then diluted in 2 mL of water. The precipitate was filtered, washed with water (2x), and redissolved in DCM before purifying by flash chromatography (0-20% *i*PrOH in DCM) to afford the desired product as a tan solid (17.2 mg, 40%).

**^1^H NMR (500 MHz, DMSO-*d*_6_)** δ 10.23 (s, 1H), 9.89 (s, 1H), 9.26 (s, 2H), 8.47 – 8.41 (m, 2H), 7.62 – 7.53 (m, 3H).

**^13^C NMR (126 MHz, DMSO-*d*_6_)** δ 194.6, 164.3, 155.8, 136.3, 131.6, 130.5, 128.9, 128.1.

**HRMS (DART+)** Exact mass calcd. for C_11_H_10_N_3_S [M+H]^+^ = 216.05899; found 216.05892.

***tert-*butyl ((3-(1-phenyl-1H-pyrazol-4-yl)-1,2,4-oxadiazol-5-yl)methyl)carbamate, 14.**

 Prepared according to General Procedure **E** on a 0.16 mmol scale using compound **5** (33.1 mg, 0.16 mmol, 1.0 eq.), HCTU (74.8 mg, 0.18 mmol, 1.1 eq.), Boc-Gly-OH (29.2 mg, 0.16 mmol, 1.0 eq.) and DIPEA (60 μL, 0.34 mmol, 2.1 eq.) in DMF (0.81 mL). After 1 h at 110 °C, the reaction was complete by LCMS and 1.5 mL water was added. The solids were filtered off (keep solids), washed with water (3x), and dried to isolate the desired product as a tan solid (38.7 mg, 69%).

**^1^H NMR (500 MHz, DMSO-*d*_6_)** δ 9.18 (s, 1H), 8.25 (s, 1H), 8.00 – 7.94 (m, 2H), 7.72 (t, *J* = 5.9 Hz, 1H), 7.58 – 7.50 (m, 2H), 7.42 – 7.35 (m, 1H), 4.49 (d, *J* = 5.9 Hz, 2H), 1.41 (s, 9H).

**^13^C NMR (126 MHz, DMSO-*d*_6_)** δ 177.8, 162.4, 155.6, 139.8, 139.0, 129.6, 128.1, 127.2, 118.9, 110.7, 78.8, 36.6, 28.1.

**HRMS (DART+)** Exact mass calcd. for C_17_H_20_N_5_O_3_ [M+H]^+^ = 342.15607; found 342.15663.

**5-chloro-2-(1-phenyl-1H-pyrazol-4-yl)pyrimidine, 15.**

Prepared according to General Procedure **A** on a 0.2 mmol scale using compound **1-Cl** (61 mg, 0.2 mmol, 1.0 eq.), DMF (0.40 mL), amidine **6** (37 mg, 0.2 mmol, 1.0 eq.), and *N*-methylmorpholine (55 μL, 2.5 mmol, 2.5 eq.) at 70 °C. After 3 h, the reaction was complete by LCMS and 5 mL water was added. The solids were filtered off (keep solids), washed with water (3x), and dried to isolate the desired product as a pale red solid (25 mg, 48%). No further purification was required.

**^1^H NMR (500 MHz, DMSO-*d*_6_)** δ 9.16 (d, *J* = 0.6 Hz, 1H), 8.93 (s, 2H), 8.34 (d, *J* = 0.6 Hz, 1H), 7.99 – 7.96 (m, 2H), 7.55 – 7.50 (m, 2H), 7.39 – 7.33 (m, 1H).

**^13^C NMR (126 MHz, DMSO-*d*_6_)** δ 158.3, 156.0, 140.8, 139.2, 129.6, 128.6, 127.5, 127.0, 123.4, 118.8.

**HRMS (ESI+)** Exact mass calcd. for C_13_H_11_N_4_Cl^35^ [M+H]^+^ = 257.05885; found 257.05863.

**6-methyl-2-(1-phenyl-1H-pyrazol-4-yl)pyrimidin-4-ol, 16.**

Prepared according to General Procedure **F** on a 0.2 mmol scale using amidine **6** (37 mg, 0.2 mmol, 1.0 eq.), ethyl 3-oxobutanoate (26 μL, 0.20 mmol, 1.0 eq.) and K_2_CO_3_ (42 mg, 0.30 mmol, 1.5 eq.) at 70 °C. After 24 h, the reaction was concentrated at reduced pressure.* The crude reaction mixture was subject to flash column chromatography on C18 (0-80% MeCN in H_2_O) to afford the desired product as a white solid (19 mg, 38%).

**Practical note: The reaction was not fully complete by LCMS. Conversion was not improved when running the reaction for 48 h.*

**^1^H NMR (500 MHz, DMSO-*d*_6_)** δ 9.18 (d, *J* = 0.7 Hz, 1H), 8.41 (d, *J* = 0.6 Hz, 1H), 7.89 – 7.83 (m, 2H), 7.59 – 7.53 (m, 2H), 7.41 – 7.37 (m, 1H), 6.09 (s, 1H), 3.33 (s, 1OH), 2.24 (d, *J* = 0.9 Hz, 3H).

**^13^C NMR (126 MHz, DMSO-*d*_6_)** δ 164.8, 162.6, 140.5, 139.0, 129.8, 129.7, 128.8, 127.3, 118.8, 109.7, 23.6. *One pyrimidone carbon was not observed.*

**HRMS (ESI+)** Exact mass calcd. for C_14_H_13_N_4_O [M+H]^+^ = 253.10839; found 253.10851.

**ethyl 2-(1-phenyl-1H-pyrazol-4-yl)thiazole-4-carboxylate, 17.**

Prepared according to General Procedure **G** on a 0.2 mmol scale using compound **7 (**41 mg, 0.20 mmol, 1.0 eq.) and ethyl bromopyruvate (30 μL, 0.24 mmol, 1.2 eq.) in EtOH (1.0 mL). After 1 h at 70 °C, the reaction was complete by LCMS. The reaction mixture was concentrated at reduced pressure and subject to flash silica gel chromatography (20–100% EtOAc in hexanes) to afford the desired product as a pale yellow solid (39.7 mg, 98%).

**^1^H NMR (500 MHz, CDCl_3_)** δ 8.55 (d, *J* = 0.7 Hz, 1H), 8.15 (d, *J* = 0.7 Hz, 1H), 8.09 (s, 1H), 7.77 – 7.72 (m, 2H), 7.53 – 7.42 (m, 2H), 7.39 – 7.31 (m, 1H), 4.45 (q, *J* = 7.1 Hz, 2H), 1.43 (t, *J* = 7.1 Hz, 3H).

**^13^C NMR (126 MHz, CDCl_3_)** δ 161.5, 160.8, 147.9, 139.7, 139.6, 129.8, 127.5, 125.9, 125.9, 119.5, 118.8, 61.7, 14.5.

**HRMS (ESI+)** Exact mass calcd. for C_15_H_14_N_3_O_2_S^32^ [M+H]^+^ = 300.07994; found 300.07951.

***tert*-butyl ((3-(6-phenylpyridin-3-yl)-1,2,4-oxadiazol-5-yl)methyl)carbamate, 18.**

Prepared according to General Procedure **E** on a 0.2 mmol scale using compound **8** (98 mg, 0.20 mmol, 1.0 eq.), HCTU (91 mg, 0.22 mmol, 1.1 eq.), Boc-Gly-OH (70 mg, 0.2 mmol, 1.0 eq.) and DIPEA (70 μL, 0.40 mmol, 2.0 eq.) in DMF (1.0 mL). After 1 h at 110 °C, the reaction was complete by LCMS. The crude reaction mixture was subject to flash column chromatography on silica gel (0–60% EtOAc in hexanes) to afford the desired product as a white solid (24 mg, 34%).

**^1^H NMR (500 MHz, CDCl_3_)** δ 9.42 – 9.28 (m, 1H), 8.39 (dd, *J* = 8.3, 2.2 Hz, 1H), 8.09 – 8.01 (m, 2H), 7.88 – 7.81 (m, 1H), 7.55 – 7.41 (m, 3H), 5.34 (s, 1H), 4.66 (d, *J* = 6.0 Hz, 2H), 1.49 (s, 9H).

**^13^C NMR (126 MHz, CDCl_3_)** δ 177.1, 166.7, 159.9, 155.6, 148.8, 138.6, 135.6, 129.9, 129.0, 127.3, 121.2, 120.4, 81.0, 37.3, 28.4.

**HRMS (ESI+)** Exact mass calcd. for C_19_H_21_N_3_O_3_ [M+H]^+^ = 353.16082; found 353.16078.

**5-chloro-2-(6-phenylpyridin-3-yl)pyrimidine, 19.**

 Prepared according to General Procedure **A** on a 0.2 mmol scale using compound **1-Cl** (61 mg, 0.2 mmol, 1.0 eq.), DMF (0.40 mL), amidine **9** (39 mg, 0.2 mmol, 1.0 eq.), and *N*-methylmorpholine (55 μL, 2.5 mmol, 2.5 eq.) at 70 °C. After 3 h, the reaction was complete by LCMS and 5 mL water was added. The solids were filtered off (keep solids), washed with water (3x), and dried to isolate the desired product as a beige solid (42 mg, 79%). No further purification was required.

**^1^H NMR (500 MHz, CDCl_3_)** δ 9.68 (dd, *J* = 2.3, 0.9 Hz, 1H), 8.78 (s, 2H), 8.71 (dd, *J* = 8.3, 2.2 Hz, 1H), 8.13 – 8.07 (m, 2H), 7.86 (dd, *J* = 8.4, 0.9 Hz, 1H), 7.53 – 7.49 (m, 2H), 7.47 – 7.44 (m, 1H).

**^13^C NMR (126 MHz, CDCl_3_**) δ 161.0, 159.3, 156.0, 150.0, 138.9, 136.4, 130.6, 130.0, 129.7, 129.0, 127.3, 120.3.

**HRMS (ESI+)** Exact mass calcd. for C_15_H_11_N_3_Cl^35^ [M+H]^+^ = 268.06415; found 268.06411.

**6-methyl-2-(6-phenylpyridin-3-yl)pyrimidin-4(3H)-one, 20.**

Prepared according to General Procedure **F** on a 0.2 mmol scale using amidine **9** (39 mg, 0.2 mmol, 1.0 eq.), ethyl 3-oxobutanoate (26 μL, 0.20 mmol, 1.0 eq.) and K_2_CO_3_ (42 mg, 0.30 mmol, 1.5 eq.) at 70 °C. After 16 h, the reaction was concentrated at reduced pressure.* The crude reaction mixture was subject to flash column chromatography on silica gel (0-40% *i*PrOH in DCM) to afford the desired product as a white solid (20 mg, 38%).

**Practical note: The reaction was not fully complete by LCMS. Conversion was not improved when running the reaction for 24 h or 48 h.*

**^13^C NMR (126 MHz, DMSO-*d*_6_)** δ 166.2, 1578.0, 148.8, 137.7, 136.4, 129.8, 129.7, 128.9, 128.9, 128.1, 126.9, 119.8, 119.6, 23.5.

**HRMS (ESI+)** Exact mass calcd. for C_16_H_14_N_3_O [M+H]^+^ = 264.11314; found 264.11389.

**ethyl 2-(6-phenylpyridin-3-yl)thiazole-4-carboxylate, 21.**

Prepared according to General Procedure **G** on a 0.2 mmol scale using compound **10** (43 mg, 0.20 mmol, 1.0 eq.) and ethyl bromopyruvate (30 mL, 0.24 mmol, 1.2 eq.) in EtOH (1.0 mL). After 1 h at 70 °C, the reaction was complete by LCMS. ~ 5 mL H_2_O was added and the insoluble solids filtered off (keep solids) and washed with H_2_O (3x) to afford the desired product as a pale-yellow solid (57 mg, 92%). No further purification was required.

**^1^H NMR (500 MHz, CDCl_3_)** δ 9.38 (d, *J* = 2.2 Hz, 1H), 8.92 (d, *J* = 8.5 Hz, 1H), 8.33 (s, 1H), 8.23 (dd, *J* = 6.6, 2.8 Hz, 2H), 8.10 (d, *J* = 8.4 Hz, 1H), 7.67 – 7.60 (m, 3H), 4.51 – 4.45 (m, 2H), 1.45 (t, *J* = 7.1 Hz, 3H).

**^13^C NMR (126 MHz, CDCl_3_)** δ 184.8, 161.9, 160.9, 155.1, 149.1, 142.5, 140.3, 132.5, 129.9, 129.3, 129.1, 128.3, 123.7, 62.0, 14.4.

**HRMS (ESI+)** Exact mass calcd. for C_17_H_15_N_2_O_2_S^32^ [M+H]^+^ = 311.08487; found 311.08484.

***tert*-butyl ((3-(2-phenylpyrimidin-5-yl)-1,2,4-oxadiazol-5-yl)methyl)carbamate, 22.**

 Prepared according to General Procedure **E** on a 0.2 mmol scale using compound **11** (29 mg, 0.20 mmol, 1.0 eq.), HCTU (91 mg, 0.22 mmol, 1.1 eq.), Boc-Gly-OH (70 mg, 0.2 mmol, 1.0 eq.) and DIPEA (70 mL, 0.40 mmol, 2.0 eq.) in DMF (1.0 mL). After 1 h at 110 °C, the reaction was complete by LCMS. The crude reaction mixture was subject to flash column chromatography on silica gel (0–60% EtOAc in hexanes) to afford the desired product as a white solid (24 mg, 34%)

**^1^H NMR (500 MHz, DMSO-*d*_6_)** δ 9.42 (s, 2H), 8.52 – 8.44 (m, 2H), 7.80 (t, J = 5.9 Hz, 1H), 7.66 – 7.55 (m, 3H), 4.56 (d, J = 5.9 Hz, 2H), 1.41 (s, 9H).

**^13^C NMR (126 MHz, DMSO-*d*_6_)** δ 178.9, 165.1, 164.1, 155.8, 155.7, 136.2, 131.8, 128.9, 128.2, 118.8, 78.9, 60.5, 28.1.

**HRMS (DART+)** Exact mass calcd. for C_18_H_20_N_5_O_3_ [M+H]^+^ = 354.15607; found 354.15658.

**5-chloro-2'-phenyl-2,5'-bipyrimidine, 23.**

 Prepared according to General Procedure **A** on a 0.19 mmol scale with a slight modification using **1-Cl** (63.3 mg, 0.206 mmol, 1.1 eq.), compound **12** (37 mg, 0.19 mmol, 1.0 eq.), and *N*-methylmorpholine (51 μL, 0.48 mmol, 2.5 eq.) in DMF (0.25 mL), at 70 °C. After 3 h, the reaction was complete by LCMS and 2 mL water was added. The solids were filtered off (keep solids), washed with water (3x), and dried to isolate the desired product as a beige solid (16.8 mg, 33%). No further purification was required.

**^1^H NMR (700 MHz, PhMe-*d8*)** δ 9.70 (d, *J* = 1.8 Hz, 2H), 8.88 (dd, *J* = 6.9, 1.2 Hz, 2H), 8.00 (t, *J* = 2.1 Hz, 2H), 7.30 (td, *J* = 6.9, 3.8 Hz, 2H), 7.26 – 7.18 (m, 1H).

**^13^C NMR (175 MHz, PhMe-*d8*)** δ 165.8^‡^, 165.7^‡^, 159.1^‡^, 156.8^†^, 155.3^†^, 131.1^†^, 129.6^‡^, 128.9^†^, 128.5^†^, 127.3^‡^.*

**HRMS (DART+)** Exact mass calcd. for C_14_H_10_N_4_Cl [M+H]^+^ = 269.05885; found 269.0519.

**Analytical notes: due to poor solubility, several ^13^C NMR chemical shifts were collected using HSQC^†^ and HMBC^‡^ cross-peak experiments.*

**6-methyl-2'-phenyl-[2,5'-bipyrimidin]-4(3H)-one, 24.**

Prepared according to General Procedure **F** on a 0.2 mmol scale using compound **12** (18.0 mg, 0.074 mmol, 1.0 eq.), ethyl 3-oxobutanoate (14 mL, 0.11 mmol, 1.5 eq.) and Et_2_N (15 mL, 0.11 mmol, 1.5 eq.) in DMF (0.37 mL), at 70 °C. After 18 h, water (~1 mL) was added. The solids were filtered off (keep solids), washed with water (3x), and dried to isolate the desired product as a beige solid (162 mg, 63%).

**1H NMR (500 MHz, DMSO-*d*_6_)** δ 9.49 (s, 2H), 8.50 – 8.44 (m, 2H), 7.63 – 7.53 (m, 3H), 6.37 (s, 1H), 2.34 (s, 3H).

**^13^C NMR (126 MHz, CDCl_3_)** δ 165.5^‡^, 164.5, 156.7, 136.4, 131.6, 128.9, 128.2, 125.6^‡^, 23.4.*

**HRMS (DART+)** Exact mass calcd. for C_15_H_13_N_4_O [M+H]^+^ = 265.10839; found 265.10927.

**Analytical notes: due to poor solubility, several ^13^C NMR chemical shifts were collected using HMBC^‡^ cross-peak experiments. Three ^13^C NMR signals were never observed.*

**ethyl 2-(2-phenylpyrimidin-5-yl)thiazole-4-carboxylate, 25.**

Prepared according to General Procedure **G** on a 0.2 mmol scale using compound **13** (17.2 mg, 0.0799 mmol, 1.0 eq.) and a 0.23 M solution of ethyl bromopyruvate (0.42 mL, 0.096 mmol, 1.2 eq.) in EtOH. After 1 h at 70 °C, the reaction was complete by LCMS. ~ 1 mL H_2_O was added and the insoluble solids filtered off (keep solids) and washed with H_2_O (3x) to afford the desired product as a tan solid (19.2 mg, 77%). No further purification was required.

**1H NMR (500 MHz, CDCl_3_)** δ 9.36 (s, 2H), 8.56 – 8.49 (m, 2H), 8.27 (s, 1H), 7.59 – 7.48 (m, 4H), 4.48 (q, J = 7.1 Hz, 2H), 1.45 (t, J = 7.1 Hz, 3H).

**^13^C NMR (126 MHz, CDCl_3_)** δ 165.9, 162.8, 161.2, 155.3, 149.1, 136.9, 131.6,128.9, 128.8, 127.9, 124.8, 61.9, 14.5.

**HRMS (DART+)** Exact mass calcd. for C_16_H_14_N_3_O_2_S [M+H]^+^ = 312.08012; found 312.08101.

**5-(1-phenyl-1H-pyrazol-4-yl)-1H-tetrazole, 26.**

Prepared according to General Procedure **H** on a 0.2 mmol scale using compound **2** (34 mg, 0.20 mmol, 1.0 eq.), sodium azide (14 mg, 0.22 mmol, 1.1 eq.), and ammonium chloride (29 mg, 0.40 mmol, 2.0 eq.) in DMF (0.2 mL). After 1 h at 110 °C, the reaction was complete by LCMS. Following reverse phase flash C18 column chromatography (0–80% MeCN in H2O), the desired product was isolated as a pale yellow solid (18.4 mg, 44%).

**^1^H NMR (400 MHz, DMSO-*d*_6_)** δ 9.15 (d, *J* = 0.7 Hz, 1H), 8.30 (d, *J* = 0.7 Hz, 1H), 8.01 – 7.89 (m, 2H), 7.60 – 7.49 (m, 2H), 7.45 – 7.35 (m, 1H).

**^13^C NMR (101 MHz, DMSO-*d*_6_)** δ 149.4, 139.6, 139.0, 129.7, 127.6, 127.3, 118.9, 108.8.

**HRMS (ESI+)** Exact mass calcd. for C_10_H_9_N_6_ [M+H]^+^ = 213.08832; found 213.08865.

**2-phenyl-5-(1H-tetrazol-5-yl)pyridine, 27.**

Prepared according to General Procedure **H** on a 0.2 mmol scale using compound **3** (36 mg, 0.20 mmol, 1.0 eq.), sodium azide (14 mg, 0.22 mmol, 1.1 eq.), and ammonium chloride (29 mg, 0.40 mmol, 2.0 eq.) in DMF (0.2 mL). After 1 h at 110 °C, the reaction was complete by LCMS. Following reverse phase flash C18 column chromatography (0–80% MeCN in H2O), the desired product was isolated as a white powder (43 mg, 96%).

**^1^H NMR (400 MHz, DMSO-*d*_6_)** δ 9.30 (dd, *J* = 2.3, 0.9 Hz, 1H), 8.46 (dd, *J* = 8.3, 2.3 Hz, 1H), 8.22 (dd, *J* = 8.4, 0.9 Hz, 1H), 8.20 – 8.17 (m, 2H), 7.59 – 7.46 (m, 3H).

**^13^C NMR (101 MHz, DMSO-*d*_6_)** δ157.7, 147.6, 137.6, 135.4, 129.9, 128.9, 126.8, 120.5, 120.0.*

**HRMS (ESI+)** Exact mass calcd. for C_12_H_10_N_5_ [M+H]^+^ = 268.06415; found 224.09307.

*Analysis note: The tetrazole-containing carbon atom was not observed in the ^13^C NMR spectrum.*

**2-phenyl-5-(1H-tetrazol-5-yl)pyrimidine, 28.**

 Prepared according to General Procedure **H** on a 0.2 mmol scale with a slight modification using compound **4** (36 mg, 0.20 mmol, 1.0 eq.), sodium azide (37 mg, 0.58 mmol, 2.9 eq.), and ammonium chloride (33.1 mg, 0.32 mmol, 3.0 eq.) in DMF (2.0 mL). After 18 h at 110 °C, the reaction was complete by LCMS. Following reverse phase flash C18 column chromatography (10–90% MeCN in H_2_O), the desired product was isolated as a white powder (23.4 mg, 52%).

**^1^H NMR (400 MHz, CD_3_OD)** δ 9.45 (s, 2H), 8.52 (dd, *J* = 7.9, 1.8 Hz, 2H), 7.58 – 7.48 (m, 3H).

**^13^C NMR (101 MHz, CD_3_OD)** δ 166.9, 156.7, 138.0, 132.7, 129.8, 129.6, 119.8.

**HRMS (DART+)** Exact mass calcd. for C_11_H_9_N_6_ [M+H]^+^ = 225.08832; found 225.08895.

**ethyl 2-(cyanomethyl)-3-oxobutanoate, 29.**

An oven-dried two-necked flask fitted with a stir bar and cooled under N_2_ was charged with THF (100 mL) and ethyl 3-oxobutanoate (6.0 mL, 50 mmol, 1.0 eq.). The solution was cooled to <0 °C (ice/brine bath) then, under a flow of N_2_, 60 wt% NaH wet with mineral oil (2.1 g, 55 mmol, 1.1 eq.) was slowly added portionwise. The septum was returned and the suspension stirred for 30 min at 0 C, then bromoacetonitrile (3.68 mL, 52.5 mmol, 1.05 eq.) was injected in one portion. The cooling bath was removed and the reaction mixture stirred overnight at rt, then quenched with aq. sat. NH_4_Cl (~ 50 mL). Volatiles were removed at reduced pressure; the organics were extracted from DCM (3x100 mL). The collected organics were dried (Na_2_SO_4_) and concentrated at reduced pressure. Following flash column chromatography on silica gel (0–10% EtOAc in hexanes) the desired product was isolated as a colourless liquid (2.9 g, 34%).

**^1^H NMR (500 MHz, CDCl_3_)** δ 4.28 (qd, *J* = 7.1, 2.5 Hz, 2H), 3.85 (t, *J* = 7.2 Hz, 1H), 2.91 – 2.76 (m, 2H), 2.37 (s, 3H), 1.32 (t, *J* = 7.1 Hz, 3H).

**^13^C NMR (126 MHz, CDCl_3_)** δ 198.9, 166.5, 117.5, 61.5, 55.3, 29.5, 15.7, 14.1.

The spectral data were consistent with the literature.^[4]^

**ethyl 2-acetyl-4-cyanobutanoate, 30.**

To a stirred solution of ethyl acetoacetate (38.0 mL, 300 mmol, 6.0 eq.) and acrylonitrile (3.3 mL, 50 mmol, 1.0 eq.) in EtOH (500 mL), was added via powder funnel sodium ethoxide (6.9 g, 100 mmol, 2.0 eq.) at 0 °C. The funnel was washed with 100 mL of EtOH. The stirred solution was warmed to rt and stirred for three days. The reaction was concentrated under reduced pressure and the residue was extracted EtOAc (3x) from H_2_O (300 mL). The combined organics were washed with brine (3x), dried (NaSO_4_), and concentrated under reduced pressure. The crude mixture was purified by flash chromatography on silica gel (10-30% EtOAc in hexanes) to give the product as colorless oil (4.04 g, 44%).

*Characterization reflects a 9:1 keto:enol ratio.*

**^1^H NMR (500 MHz, CDCl_3_)** δ 12.84 (brs, 0.1OH, enol), 4.19 (dq, J = 7.1, 1.0 Hz, 2H), 3.60 (t, J = 7.1 Hz, 0.8H, keto), 2.53 (td, J = 7.0, 0.9 Hz, 0.2H, enol), 2.46-2.36 (m, 2H), 2.25 (d, J = 0.5 Hz, 2.7 H, keto), 2.19-2.05 (m, 2H), 2.06 (d, J = 0.8 Hz, 0.2H, enol), 1.31-1.21 (m, 3H).

**^13^C NMR (126 MHz, CDCl_3_)** δ 201.3 (keto), 174.6 (enol), 172.3 (enol), 168.3 (keto), 119.4 (enol), 118.7 (keto), 97.2 (enol), 62.0 (keto), 60.8 (enol), 57.3 (keto), 29.5 (keto), 23.3 (keto), 22.7 (enol), 18.9 (enol), 17.6 (enol), 15.0 (keto), 14.2 (enol), 14.0 (keto).

The spectral data were consistent with the literature.^[5]^

**2-(5-methyl-3-oxo-2-phenyl-2,3-dihydro-1H-pyrazol-4-yl)acetonitrile, 31.**

A glass vial fitted with a stir bar was charged with EtOH (5 mL), and the β-ketoester, compound **29** (850 mg, 5.0 mmol, 1.0 eq.). While stirring at rt, phenyl hydrazine (0.50 mL, 5.0 mmol, 1.0 eq.) was added in one portion. The vial was sealed with a screw cap and the reaction mixture was stirred at gentle reflux (hotplate 80 C) for 16 h. The reaction mixture was cooled to rt and DCM was added until precipitation stopped, ~ 25 mL. The solids were filtered off (keep solids) and washed with DCM (3x), then dried, to afford the desired product as a beige solid (720 mg, 67%). No further purification was required. *

The procedure was also performed on a 1.0 mmol scale (127 mg, 60%).

**Safety note: The product irritates the eyes.*

**^1^H NMR (500 MHz, DMSO-*d*_6_)** δ 7.76 – 7.67 (m, 2H), 7.47 – 7.40 (m, 2H), 7.25 – 7.19 (m, 1H), 3.60 (s, 2H), 2.19 (s, 3H).

**^13^C NMR (126 MHz, DMSO-*d*_6_)** δ 147.3, 138.0, 128.9, 125.1, 119.9, 118.8, 11.8, 10.6. *The pyrazolone carbonyl-carbon and the nitrile-carbon were not observed.*

**HRMS (ESI+)** Exact mass calcd. for C_12_H_12_N_3_O [M+H]^+^ = 264.11314; found 214.09775.

**3-(4-hydroxy-6-methyl-2-phenylpyrimidin-5-yl)propanenitrile, 32.**

Prepared according to General Procedure **F** with a slight modification using compound **30** (1.0 g, 5.5 mmol, 1.0 eq.), benzamidine hydrochloride (0.90 g, 5.8 mmol, 1.1 eq.), K_2_CO_3_ (1.5 g, 11 mmol, 2.0 eq., *modified from 1.5 eq.*) in MeCN (28 mL, 0.2 M) at 80 °C, overnight. The crude was subject to reverse phase flash C18 column chromatography (20%−60% MeCN in H_2_O) and the collected product fractions were concentrated to remove volatiles. A precipitate was collected from the aqueous liquor to isolate the desired product as a beige solid (371 mg, 28%).

*Characterization for the tautomer as shown.*

**^1^H NMR (500 MHz, DMSO-*d*_6_)** δ 12.74 (brs, 1OH), 8.09 (d, J = 7.7 Hz, 2H), 7.59 – 7.47 (m, 3H), 3.02 – 2.61 (m, 4H), 2.38 (s, 3H).

**^13^C NMR (126 MHz, DMSO-*d*_6_)** δ 162.8, 161.1, 154.1, 132.2, 131.5, 128.6, 127.6, 120.2, 119.0, 21.6, 21.6, 15.0.

**HRMS (DART+)** Exact mass calcd. for C_14_H_14_N_3_O [M+H]^+^ = 240.11314; found 240.11245.

**3-(5-methyl-3-oxo-2-phenyl-2,3-dihydro-1H-pyrazol-4-yl)propanenitrile, 33.**

A glass vial fitted with a stir bar was charged with EtOH (9 mL), and the β-ketoester, compound **30** (1.67 g, 9.1 mmol, 1.0 eq.). While stirring at rt, phenyl hydrazine (0.89 mL, 9.1 mmol, 1.0 eq.) was added dropwise. The vial was sealed with a screw cap and the reaction mixture was stirred at 80 °C for 48 h. The reaction mixture was cooled to rt upon which precipitation was observed. Et_2_O was added until precipitation stopped, ~ 50 mL. The products were filtered and washed with Et_2_O, then dried to afford the desired product as a beige solid (1.69 g, 82%). No further purification was required.

**^1^H NMR (500 MHz, DMSO-*d*_6_)** δ 10.94 (s, 1H, NH), 7.72 (d, *J* = 8.0 Hz, 2H), 7.47 – 7.39 (m, 2H), 7.19 (t, *J* = 7.4 Hz, 1H), 2.64 (d, *J* = 5.8 Hz, 2H), 2.62 – 2.53 (m, 2H), 2.17 (s, 3H).*

**^13^C NMR (126 MHz, CDCl_3_)** δ 147.4, 136.3, 129.0, 126.1, 120.9, 119.9, 102.5, 18.9, 16.9, 11.0.*

**Analytical notes: In CDCl_3_ the ^1^H NMR spectrum showed a mixture of tautomers, DMSO-d_6_ simplified characterization. In the ^13^C NMR (DMSO-d6) multiple peaks were not observed, but characterization could be adequately performed in CDCl_3_. The pyrazolone carbonyl-carbon atom was never observed by ^13^C NMR.*

**HRMS (DART+)** Exact mass calcd. for C_12_H_12_N_3_O [M+H]^+^ = 228.11314; found 228.11336.

**2-(5-methyl-3-oxo-2-phenyl-2,3-dihydro-1H-pyrazol-4-yl)ethanethioamide, 34.**

Prepared according to General Procedure **D** on a 0.5 mmol scale using compound **31 (**110 mg, 0.50 mmol, 1.0 eq.), sodium hydrogen sulfide monohydrate (74.0 mg, 0.875 mmol, 1.75 eq.) and magnesium chloride (94 mg, 0.50 mmol, 1.0 eq.) in DMF (1.5 mL). The reaction was complete after 1 h at 60 °C where the product partially precipitated. The reaction mixture was cooled to rt, then diluted in ~10 mL H_2_O. Insoluble salts were filtered off (keep solid) and washed with H_2_O (3x) then dried to afford the desired product as a white solid (96 mg, 78%).

**^1^H NMR (500 MHz, DMSO-*d*_6_)** δ 9.40 (s, 1NH), 9.34 (s, 1NH), 7.83 – 7.73 (m, 2H), 7.42 – 7.33 (m, 2H), 7.13 (tt, *J* = 7.3, 1.2 Hz, 1H), 3.55 (s, 2H), 2.12 (s, 3H).

**^13^C NMR (126 MHz, DMSO-*d*_6_)** δ 206.4, 162.4, 158.3, 147.9, 138.8, 128.7, 124.1, 119.2, 98.0, 12.3.

**HRMS (ESI+)** Exact mass calcd. for C_12_H_14_N_3_OS^32^ [M+H]^+^ = 248.08521; found 248.08548.

**3-(4-hydroxy-6-methyl-2-phenylpyrimidin-5-yl)propanethioamide, 37.**

 Prepared according to General Procedure **D** on a 0.2 mmol scale using compound **32 (**47.7 mg, 0.2 mmol, 1.0 eq.), sodium hydrogen sulfide monohydrate (26 mg, 0.35 mmol, 1.75 eq.) and magnesium chloride (19 mg, 0.2 mmol, 1.0 eq.) in DMF (0.6 mL). The reaction was stirred at 60 °C for 24 h. The reaction mixture was cooled to rt, then diluted with ~0.5 mL H_2_O. Following reverse phase flash C18 column chromatography (5–35% MeCN in H_2_O), the desired product was isolated as a white solid (45 mg, 83%).

*Characterization for the tautomer as shown.*

**^1^H NMR (500 MHz, DMSO-*d*_6_)** δ 12.56 (brs, 1OH), 9.40 (s, 1NH), 9.24 (s, 1NH), 8.08 (d, J = 7.3 Hz, 2H), 7.58-7.46 (m, 3H), 2.83 (t*,* J = 6.2 Hz, 2H), 2.65 (t, J = 6.2 Hz, 2H), 2.36 (s, 3H).

**^13^C NMR (126 MHz, DMSO-*d*_6_)** δ 207.5, 163.0, 160.2, 153.5, 132.5, 131.3, 128.6, 127.5, 120.9, 42.4, 25.3, 21.5.

**HRMS (DART+)** Exact mass calcd. for C_14_H_16_N_3_OS [M+H]^+^ = 274.10086; found 274.10066.

**3-(4-hydroxy-6-methyl-2-phenylpyrimidin-5-yl)propanimidamide formate salt, 39.**

To a flame dried vial under N_2_ was added 1-butanol (0.38 mL, 4.1 mmol, 20 eq.) and acetyl chloride (0.15 mL, 2.1 mmol, 10 eq.) at 0 °C. After stirring for 30 min, compound **32** (50 mg, 0.2 mmol) was added. After stirring for 30 min. at 0 °C, the ice bath was removed, and the mixture was allowed to stir at rt overnight. The reaction mixture was diluted with 0.42 mL of 1-butanol, cooled to 0 °C, then ammonia was slowly added (1.6 mL, 2 M in *i*PrOH, 15 eq.). After stirring for 30 min. at 0 °C, the ice bath was removed, and the mixture was allowed to stir at rt overnight. The reaction mixture was concentrated under reduced pressure, then purified by reverse phase C18 column chromatography (5% MeCN in H_2_O) to give the desired product as a white solid (36 mg, 57%).

*Characterization for the major tautomer as shown.*

**^1^H NMR (500 MHz, DMSO-*d*_6_)** δ 9.95 (brs, 2NH), 8.93 (brs, 2NH), 8.41 (s, 0.6 OH), 8.09 (dt, J = 7.0, 1.5 Hz, 2H), 7.67-7.14 (m, 3H), 2.82 (t, J = 7.5 Hz, 2H), 2.56 (t, J = 7.5 Hz, 2H), 2.34 (s, 3H).

**^13^C NMR (126 MHz, DMSO-*d*_6_)** δ 170.6, 167.3, 164.0, 160.2, 154.5, 132.9, 131.2, 128.6, 127.5, 119.2, 30.7, 22.6, 21.3.

**HRMS (ESI+)** Exact mass calcd. for C_14_H_17_N_4_O [M+H]^+^ = 257.1397; found 257.1405.

**3-(5-methyl-3-oxo-2-phenyl-2,3-dihydro-1H-pyrazol-4-yl)propanethioamide, 40.**

Prepared according to General Procedure **D** on a 0.2 mmol scale using compound **33 (**45 mg, 0.20 mmol, 1.0 eq.), sodium hydrogen sulfide monohydrate (26 mg, 0.34 mmol, 1.75 eq.) and magnesium chloride (19 mg, 0.20 mmol, 1.0 eq.) in DMF (0.6 mL). After 2 h at 60 °C, the reaction was complete by LCMS. The reaction mixture was cooled to rt, then diluted in ~1 mL H_2_O. Following reversed phase flash C18 column chromatography (10–80% MeCN in H_2_O), the desired product was isolated as a yellow solid (42 mg, 82%).

**^1^H NMR (500 MHz, DMSO-*d*_6_)** δ 10.65 (brs, 1NH), 9.36 (brs, 1NH), 9.18 (brs, 1NH), 7.75 – 7.67 (m, 2H), 7.48 – 7.36 (m, 2H), 7.21 – 7.15 (m, 1H), 2.69 – 2.56 (m, 4H), 2.15 (s, 3H).

**^13^C NMR (126 MHz, DMSO-*d*_6_)** δ 208.3,148.1, 138.6, 129.2, 124.7, 119.4, 44.5, 22.2, 12.2.*

**HRMS (DART+)** Exact mass calcd. for C_12_H_14_N_3_OS^32^ [M+H]^+^ = 262.10068; found 262.10022.

**Analysis note: two carbon signals in the ^13^C NMR spectrum, including the pyrazolone carbonyl-bearing carbon, were not observed.*

***N*'-hydroxy-3-(5-methyl-3-oxo-2-phenyl-2,3-dihydro-1H-pyrazol-4-yl)propanimidamide, 41.**

Prepared according to General Procedure **B** on a 0.5 mmol scale using compound **33** (230 mg, 1.0 mmol, 1.0 eq.), hydroxylamine hydrochloride (76 mg, 1.1 mmol, 1.1 eq.), and Et_3_N (0.15 mL, 1.1 mmol, 1.1 eq.) in EtOH (2.50 mL). The reaction mixture was stirred at 70 °C for 24 h.* The reaction mixture was cooled to rt, upon which the mixture was acidified with 2M HCl. Following reversed phase flash C18 column chromatography (10–60% MeCN in H_2_O), the desired product was isolated as a yellow oil (156 mg, 60%).

**Practical note: The reaction was not fully complete by LCMS. Conversion was not improved when running the reaction for 24 h or 48 h. Use of excess of hydroxylamine hydrochloride leads to double addition product and was avoided.*

**^1^H NMR (400 MHz, DMSO-*d*_6_)** δ 8.21 (s, 1OH), 7.80 – 7.66 (m, 2H), 7.47 – 7.35 (m, 2H), 7.16 (tt, *J* = 7.3, 1.2 Hz, 1H), 5.89 (brs, 2NH), 2.45 (t, *J* = 6.9 Hz, 2H), 2.27 (t, *J* = 6.8 Hz, 2H), 2.09 (s, 3H).*

**^13^C NMR (101 MHz, DMSO-*d*_6_)** δ 154.4, 147.2, 138.8, 128.8, 124.4, 119.4, 31.2, 18.0, 8.6*.**

**HRMS (DART+)** Exact mass calcd. for C_12_H_12_N_3_O [M+H]^+^ = 261.13460; found 261.13534.

**Analysis notes: The ^1^H NMR contains 12 mol% triethylamine and the purity has been reflected in the final yield. In the ^13^C NMR, the pyrazolone carbonyl-bearing carbon and amino oxime-bearing carbons were not observed.*

**3-(5-methyl-3-oxo-2-phenyl-2,3-dihydro-1H-pyrazol-4-yl)propanimidamide, 42.**

To a flame dried vial cooled under N_2_ was added compound **33 (**114 mg, 0.50 mmol, 1.0 eq.) to dry EtOH (0.6 mL, 10 mmol, 20 eq.) at 0 °C. Acetyl chloride (0.36 mL, 20 mmol, 10 eq.) was added dropwise, and the reaction mixture warmed to rt and stirred for 8 h, where LCMS found full consumption of the starting material. The reaction mixture was cooled to 0 °C and ammonia (7 N in *i*PrOH, 1.4 mL, 10 mmol, 20 eq.) was added dropwise. The reaction mixture was warmed to rt and stirred overnight, then concentrated at reduced pressure. Following reversed phase flash C18 column chromatography (10–50% MeCN in H_2_O), the desired product was isolated as a white solid (117 mg, 96%).

**^1^H NMR (500 MHz, DMSO-*d*_6_)** δ 9.36 (brs, 1NH), 9.10 (s, 1NH), 8.34 (brs, 1NH), 7.86 – 7.80 (m, 2H), 7.37 – 7.31 (m, 2H), 7.10 – 7.06 (m, 1H), 2.58 – 2.52 (dq, *J* = 11.0, 6.0 Hz, 4H), 2.08 (s, 3H).

**^13^C NMR (126 MHz, DMSO-*d*_6_)** *δ* 170.7, 158.4, 147.0, 139.5, 128.5, 123.5, 118.8, 98.1, 32.8, 19.2, 12.3.

**HRMS (DART+)** Exact mass calcd. for C_12_H_12_N_3_O [M+H]^+^ = 245.13969; found 245.13949.

**ethyl 2-((5-methyl-3-oxo-2-phenyl-2,3-dihydro-1H-pyrazol-4-yl)methyl)thiazole-4-carboxylate, 43.**

 Prepared according to General Procedure **G** on a 0.2 mmol scale using thioamide **34** (49 mg, 0.20 mmol, 1.0 eq.) and ethyl bromopyruvate (30 μL, 0.24 mmol, 1.2 eq.) in EtOH (1.0 mL). After 1 h at 70 °C, the reaction was complete by LCMS. ~ 5 mL H_2_O was added and the insoluble solids filtered off (keep solids) and washed with H_2_O (3x) to afford the desired product as a white solid (16 mg, 24%). No further purification was required.

*Characterized as a mixture of tautomers in solution and reported as observed.*

**^1^H NMR (400 MHz, DMSO-*d*_6_)** δ 8.27 (s, 0.9H), 8.06 (s, 0.1H), 7.71 – 7.60 (m, 2H), 7.42 – 7.32 (m, 2H), 7.20 – 7.10 (m, 1H), 4.22 (q, *J* = 7.1 Hz, 1.8H), 4.15 – 4.11 (m, 0.2H), 3.96 (s, 2H), 2.05 (s, 2H), 1.23 (t, *J* = 7.1 Hz, 2.7H), 1.17 – 1.12 (m, 1H), 1.07 (t, *J* = 7.0 Hz, 0.3H).

**^13^C NMR (126 MHz, DMSO-*d*_6_)** δ 167.4, 166.6, 160.8, 145.8, 145.1, 128.9, 115.1, 82.9, 62.5, 60.6, 34.8, 25.9, 14.2, 13.9.

**HRMS (ESI+)** Exact mass calcd. for C_17_H_18_N_3_O_3_S^32^ [M+H]^+^ = 344.10699; found 344.10676.

**ethyl 2-(2-(4-hydroxy-6-methyl-2-phenylpyrimidin-5-yl)ethyl)thiazole-4-carboxylate, 44.**

 Prepared according to General Procedure **G** on a 0.11 mmol scale using compound **37** (30 mg, 0.11 mmol, 1.0 eq.) and ethyl bromopyruvate (16 μL, 0.13 mmol, 1.2 eq.) in EtOH (0.46 mL). After 1 h at 70 °C, the reaction was complete by LCMS. The reaction mixture was concentrated under reduced pressure, then subject to reverse phase C18 column chromatography (5-30%, 30-45% MeCN in H_2_O) to afford the desired product as a white solid (29 mg, 71%).

*Characterization for the tautomer as shown.*

**^1^H NMR (500 MHz, DMSO-*d*_6_)** δ 12.65 (brs, 1OH), 8.39 (s, 1H), 8.09 (dt, J = 7.0, 1.4 Hz, 2H), 7.58-7.45 (m, 3H), 4.29 (q, J = 7.1 Hz, 2H), 3.21 (t, J = 7.7 Hz, 2H), 2.89 (t, J = 7.7 Hz, 2H), 2.24 (s, 3H), 1.29 (t, J = 7.1 Hz, 3H).

**^13^C NMR (126 MHz, DMSO-*d*_6_)** δ 170.3, 163.2, 160.8, 160.2, 153.9, 145.6, 132.4, 131.4, 129.0, 128.6, 127.5, 120.0, 60.7, 30.8, 25.8, 21.2, 14.2.

**HRMS (DART+)** Exact mass calcd. for C_19_H_20_N_3_O_3_S [M+H]^+^ = 370.12199; found 370.12307.

***tert*-butyl((3-(2-(4-hydroxy-6-methyl-2-phenylpyrimidin-5-yl)ethyl)-1,2,4-oxadiazol-5-yl)methyl)carbamate, 45.**

 To a flame dried vial under N_2_ was added dried 1-butanol (0.38 mL, 4.1 mmol, 20 eq.) and acetyl chloride (0.15 mL, 2.1 mmol, 10 eq) at 0 °C. After stirring for 30 min, compound **32** (49 mg, 0.20 mmol, 1.0 eq.) was added. After stirring for 30 min. at 0 °C, the ice bath was removed, and the mixture was allowed to stir at rt overnight. The reaction mixture was diluted with 0.8 mL of 1-butanol, then hydroxylamine hydrochloride (21 mg, 0.3 mmol, 1.5 eq.) was then added and the reaction mixture cooled to 0 °C. Triethylamine was added (0.35 mL, 2.5 mmol, 12 eq.), then  after stirring for 30 min. at 0 °C, the ice bath was removed, and the mixture was allowed to stir at rt overnight. The reaction mixture was concentrated under reduced pressure, then subject to reverse phase C18 column chromatography (5% MeCN in H_2_O) to give a white solid that contained the desired amino oxime **38** and an unknown byproduct (total mass, 37.2 mg). General Procedure **E** was then immediately followed using compound **38** (37.2 mg, 0.14 mmol, 1.0 eq.), HCTU (62 mg, 0.15 mmol, 1.1 eq.), Boc-Gly-OH (24 mg, 0.14 mmol, 1.0 eq.) and DIPEA (50 mL, 0.27 mmol, 2.0 eq.) in DMF (0.68 mL) at 110 °C, overnight. Once cooled the crude reaction mixture was subject to reverse phase C18 column chromatography (5-25%, 25-60% MeCN in H_2_O) to afford the desired product as a brown solid (12 mg, 15% over 2 steps).

*Characterization for the tautomer as shown.*

**^1^H NMR (500 MHz, DMSO-*d*_6_)** δ 12.61 (brs, 1OH), 8.08 (d, J = 7.3 Hz, 2H), 7.66 (t, J = 6.0 Hz, 1NH), 7.55 (t, J = 7.3 Hz, 1H), 7.50 (t, J = 7.4 Hz, 2H), 4.39 (d, J = 6.0 Hz, 2H), 2.89 (t, J = 6.9 Hz, 2H), 2.83 (t, J = 6.9 Hz, 2H), 2.24 (s, 3H), 1.39 (s, 9H).

**^13^C NMR (126 MHz, DMSO-*d*_6_)** δ 177.4, 169.6, 163.0, 160.2, 155.6, 153.7, 132.4, 131.4, 128.6, 127.5, 120.1, 78.7, 36.5, 28.1, 23.6 ,23.3, 21.3.

**HRMS (DART+)** Exact mass calcd. for C_21_H_26_N_5_O_4_ [M+H]^+^ = 412.19793; found 412.19864.

**5-(2-(5-chloropyrimidin-2-yl)ethyl)-6-methyl-2-phenylpyrimidin-4-ol, 46.**

 Prepared according to General Procedure **A** on a 0.12 mmol scale using compound **1-Cl** (41 mg, 0.12 mmol, 1.1 eq.), DMF (0.36 mL), amidine **39** (36.4 mg, 0.12 mmol, 1.0 eq.), and *N*-methylmorpholine (30 mL, 0.3 mmol, 2.5 eq.) at 70 °C. After 3.5 h, the reaction was cooled to rt and the neat reaction mixture subjected to reverse phase C18 column chromatography (5%-45% MeCN in H_2_O) to afford the desired product as a white solid (10 mg, 25%).

*Characterization for the tautomer as shown.*

**^1^H NMR (500 MHz, CDCl_3_)** δ 12.86 (s, 1H), 8.32 (s, 2H), 8.01 (dd, *J* = 6.9, 2.8 Hz, 2H), 7.58 – 6.97 (m, 3H), 3.02 (t, *J* = 7.6 Hz, 2H), 2.86 (t, *J* = 7.6 Hz, 2H), 2.11 (s, 3H).

**^13^C NMR (126 MHz, CDCl_3_)** δ 168.6, 165.0, 162.1, 155.5, 153.7, 132.4, 131.7, 129.1, 129.0, 127.7, 121.5, 35.9, 25.3, 21.9.

**HRMS (DART+)** Exact mass calcd. for C_17_H_16_N_4_OCl [M+H]^+^ = 327.10166; found 327.10072.

**2-(2-(4-hydroxy-6-methyl-2-phenylpyrimidin-5-yl)ethyl)-6-methylpyrimidin-4(3H)-one, 47.**

Prepared according to General Procedure **F** on a 0.12 mmol scale using amidine **39** (36 mg, 0.12 mmol, 1.0 eq.), ethyl 3-oxobutanoate (20 μL, 0.15 mmol, 1.2 eq.) and K_2_CO_3_ (33 mg, 0.24 mmol, 2.0 eq.) at 70 °C. After stirring overnight, the reaction mixture was concentrated under reduced pressure. The crude reaction mixture was subject to reverse phase flash C18 column chromatography (5-45% MeCN in H_2_O) to afford the desired product as a white solid (20 mg, 38%).

*Characterization for the tautomer as shown.*

**^1^H NMR (500 MHz, DMSO-*d*_6_)** δ 13.03 – 11.95 (m, 2H), 8.08 (dt, *J* = 7.0, 1.4 Hz, 2H), 7.59 – 7.46 (m, 3H), 6.01 (s, 1H), 2.83 (t, *J* = 7.5 Hz, 2H), 2.69 (t, *J* = 7.5 Hz, 2H), 2.34 (s, 0.6H), 2.28 (s, 2.4H), 2.14 (d, *J* = 0.9 Hz, 3H).

**^13^C NMR (100 MHz, DMSO-*d*_6_)** δ 164.2, 163.0, 160.9, 160.7, 160.0, 153.6, 132.5, 131.3, 128.6, 127.5, 120.4 110.0, 32.4, 23.4, 23.2, 20.9.*

**HRMS (DART+)** Exact mass calcd. for C_18_H_19_N_4_O_2_ [M+H]^+^ = 323.15025; found 323.15055.

** Analytical note: several peaks were not detected by ^13^C NMR but could be identified by the provided ^1^H-^13^C HMBC cross-peaks.*

***tert*-butyl((3-(2-(5-methyl-3-oxo-2-phenyl-2,3-dihydro-1H-pyrazol-4-yl)ethyl)-1,2,4-oxadiazol-5-yl)methyl)carbamate, 48.**

Prepared according to General Procedure **E** on a 0.2 mmol scale using compound **41** (104 mg, 0.40 mmol, 1.0 eq.), HCTU (182 mg, 0.44 mmol, 1.1 eq.), Boc-Gly-OH (70 mg, 0.2 mmol, 1.0 eq.) and DIPEA (140 μL, 0.80 mmol, 2.0 eq.) in DMF (2.0 mL). After 15 min. at 110 °C, the reaction was complete by LCMS. The crude reaction mixture was subject to reversed phase flash C18 column chromatography on silica gel (10-80% MeCN in H_2_O) to afford the desired product as a white solid (9 mg, 6%).

**^1^H NMR (500 MHz, DMSO-*d*_6_)** δ 10.72 (brs, 1NH), 7.71 (d, *J* = 8.1 Hz, 2H), 7.66 (t, *J* = 6.0 Hz, 1H), 7.46 – 7.37 (m, 2H), 7.18 (t, *J* = 7.4 Hz, 1H), 4.39 (d, *J* = 6.0 Hz, 2H), 2.85 (t, *J* = 7.4 Hz, 2H), 2.68 – 2.53 (m, 2H), 2.03 (s, 3H), 1.39 (s, 9H).

**^13^C NMR (126 MHz, DMSO-*d*_6_)** δ 177.3, 169.7, 155.6, 147.1*, 137.6*, 128.8, 124.5*, 118.3*, 104.6*, 78.7, 36.5, 28.1, 24.5*, 19.6, 10.6*.

**HRMS (ESI+)** Exact mass calcd. for C_19_H_21_N_3_O_3_ [M+H]^+^ = 400.1979; found 400.1977.

**Analytical note: the highlighted signals in the ^13^C NMR spectrum were not observed directly and were identified by the provided ^1^H-^13^C HSQC and ^1^H-^13^C HMBC cross-peaks. The pyrazolone carbonyl-carbon was not observed.*

**4-(2-(5-chloropyrimidin-2-yl)ethyl)-5-methyl-2-phenyl-1,2-dihydro-3H-pyrazol-3-one, 49.**

 Prepared according to General Procedure **A** on a 0.2 mmol scale using compound **1-Cl** (21 mg, 0.07 mmol, 1.0 eq.), DMF (0.20 mL), amidine **42** (17 mg, 0.07 mmol, 1.0 eq.), and *N*-methylmorpholine (18 μL, 0.16 mmol, 2.3 eq.) at 70 °C. After 16 h, the reaction was complete by LCMS and 1 mL water was added. Following reversed phase flash C18 column chromatography (0–80% MeCN in H_2_O), the desired product was isolated as a white solid (24.3 mg, 39%).

**^1^H NMR (500 MHz, DMSO-*d*_6_)** δ 10.76 (brs, 1H, NH), 8.85 (s, 2H), 7.78 – 7.68 (m, 2H), 7.44 – 7.36 (m, 2H), 7.16 (tt, *J* = 7.3, 1.2 Hz, 1H), 3.09 – 3.02 (m, 2H), 2.71 (t, *J* = 7.7 Hz, 2H), 2.01 (s, 3H).

**^13^C NMR (126 MHz, DMSO-*d*_6_**) δ 173.0, 167.0, 162.9, 155.5, 137.7, 128.9, 128.5, 124.7, 117.9, 78.4, 32.8, 31.0, 12.7.

**HRMS (ESI+)** Exact mass calcd. for C_15_H_11_N_3_Cl^35^ [M+H]^+^ = 315.10072; found 315.10170.

**6-methyl-2-(2-(5-methyl-3-oxo-2-phenyl-2,3-dihydro-1H-pyrazol-4-yl)ethyl)pyrimidin-4(3H)-one, 50.**

Prepared according to General Procedure **F** on a 0.2 mmol scale using amidine **42** (39 mg, 0.2 mmol, 1.0 eq.), ethyl 3-oxobutanoate (26 mL, 0.20 mmol, 1.0 eq.) and K_2_CO_3_ (42 mg, 0.30 mmol, 1.5 eq.) at 70 °C. After 16 h, the reaction was concentrated at reduced pressure. The reaction was concentrated in vacuo, suspended in 1:1 DMSO/H_2_O (1 mL). The crude reaction mixture was subject to reversed phase flash C18 column chromatography on silica gel (10-80% MeCN in H_2_O) to afford the desired product as a yellow solid (16.6 mg, 26%).

*Characterization for the tautomer as shown.*

**^1^H NMR (500 MHz, DMSO-*d*_6_)** δ 7.79 – 7.71 (m, 2H), 7.44 – 7.35 (m, 2H), 7.14 (tt, *J* = 7.4, 1.2 Hz, 1H), 6.00 (d, *J* = 1.1 Hz, 1H), 2.70 (t, *J* = 7.6 Hz, 2H), 2.60 (t, *J* = 7.3 Hz, 2H), 2.50 (p, *J* = 1.9 Hz, 3H), 2.05 (s, 3H).

**^13^C NMR (126 MHz, DMSO-*d*_6_)** δ 163.5, 162.8, 161.5, 147.4, 138.6, 128.7, 128.7, 124.1, 119.0, 109.9, 34.1, 23.1, 19.5, 11.7.*

**HRMS (ESI+)** Exact mass calcd. for C_16_H_14_N_3_O [M+H]^+^ = 311.1503; found 311.1509.

**Analytical note: the pyrazolone carbonyl-carbon was not observed in the ^13^C NMR spectrum.*

**ethyl 2-(2-(5-methyl-3-oxo-2-phenyl-2,3-dihydro-1H-pyrazol-4-yl)ethyl)thiazole-4-carboxylate, 51.**

Prepared according to General Procedure **G** on a 0.5 mmol scale using compound **40** (131 mg, 0.50 mmol, 1.0 eq.) and ethyl bromopyruvate (76 μL, 0.60 mmol, 1.2 eq.) in EtOH (2.0 mL). After 1 h at 70 °C, the reaction was complete by LCMS. The crude reaction mixture was subject to reversed phase flash C18 column chromatography on silica gel (10-80% MeCN in H_2_O) to afford the desired product as a yellow solid (135 mg, 76%).

**^1^H NMR (400 MHz, DMSO-*d*_6_)** δ 10.77 (s, 1H), 8.39 (s, 1H), 7.77 – 7.67 (m, 2H), 7.46 – 7.39 (m, 2H), 7.18 (t, *J* = 7.4 Hz, 1H), 4.29 (q, *J* = 7.1 Hz, 2H), 3.19 (t, *J* = 7.4 Hz, 2H), 2.70 (s, 2H), 2.04 (s, 3H), 1.30 (t, *J* = 7.1 Hz, 3H).

**^13^C NMR (126 MHz, DMSO-*d*_6_)** δ 170.7, 160.8, 148.0*, 145.5, 138.1*, 128.9, 128.8, 124.5, 118.1, 60.7, 31.8*, 22.0, 14.2, 11.0*.

**HRMS (DART+)** Exact mass calcd. for C_12_H_12_N_3_O [M+H]^+^ = 358.12181; found 358.12159.

**Analytical note: the highlighted signals in the ^13^C NMR spectrum were not observed directly and were identified by the provided ^1^H-^13^C HSQC and ^1^H-^13^C HMBC cross-peaks. The pyrazolone carbonyl-carbon was not observed.*

**4-((1H-tetrazol-5-yl)methyl)-5-methyl-2-phenyl-1,2-dihydro-3H-pyrazol-3-one, 52.**

 Prepared according to General Procedure **H** on a 0.2 mmol scale using compound **31** (43 mg, 0.20 mmol, 1.0 eq.), sodium azide (14 mg, 0.22 mmol, 1.1 eq.), and ammonium chloride (29 mg, 0.40 mmol, 2.0 eq.) in DMF (0.2 mL). After 1 h at 110 °C, the reaction was complete by LCMS. Following reverse phase flash C18 column chromatography (20–80% MeCN in H_2_O), the desired product was isolated as a beige solid solid (29 mg, 56%).

**^1^H NMR (400 MHz, DMSO-*d*_6_)** δ 8.99 (brs, 2NH), 8.07 – 7.90 (m, 2H), 7.86 (dd, *J* = 15.4, 8.5 Hz, 1H), 7.40 – 7.29 (m, 2H), 7.12 – 7.03 (m, 1H), 2.15 (s, 3H).

**^1^H NMR (400 MHz, DMSO-*d*_6_+D_2_O spike)** δ 8.00 – 7.92 (m, 2H), 7.85 (s, 1H), 7.39 – 7.32 (m, 2H), 7.08 (tt, *J* = 7.3, 1.2 Hz, 1H), 2.14 (s, 3H).

**^13^C NMR (101 MHz, DMSO-*d*_6_)** δ 165.1, 152.5, 148.7, 139.5, 128.6, 123.2, 117.4, 99.5, 12.4.*

**HRMS (ESI+)** Exact mass calcd. for C_12_H_12_N_6_O [M+H]^+^ = 257.11454; found 257.11557.

**Analysis notes*: *thee pyrazolone carbonyl-carbon was not observed.* The characterization data are consistent with the following tautomer in solution, **4-((1,4-dihydro-5H-tetrazol-5-ylidene)methyl)-5-methyl-2-phenyl-1,2-dihydro-3H-pyrazol-3-one.**

**5-(2-(1H-tetrazol-5-yl)ethyl)-6-methyl-2-phenylpyrimidin-4-ol, 53.**

 Prepared according to General Procedure **H** on a 0.2 mmol scale using compound **32** (48 mg, 0.20 mmol, 1.0 eq.), sodium azide (14 mg, 0.22 mmol, 1.1 eq.), and ammonium chloride (22 mg, 0.40 mmol, 2.0 eq.) in DMF (0.4 mL) at 110 °C, overnight.* Following reverse phase flash C18 column chromatography (5–30% MeCN in H_2_O), the desired product was isolated as a white solid (39 mg, 69%) with starting material recovered (9 mg, 20%).

** Practical note: The reaction never completed by LCMS analysis.*

*Characterization for the tautomer as shown.*

**^1^H NMR (500 MHz, DMSO-*d*_6_)** δ 8.12 – 8.04 (m, 2H), 7.58 – 7.45 (m, 3H), 3.10 (t, *J* = 7.6 Hz, 2H), 2.86 (t, *J* = 7.6 Hz, 2H), 2.14 (s, 3H).

**^13^C NMR (126 MHz, DMSO-*d*_6_)** δ 163.2, 160.0, 155.4, 153.8, 132.4, 131.4, 128.6, 127.5, 119.9, 24.1, 21.4, 21.0.

**HRMS (DART+)** Exact mass calcd. for C_14_H_15_N_6_O [M+H]^+^ = 283.13019; found 283.12981.

**4-(2-(1H-tetrazol-5-yl)ethyl)-5-methyl-2-phenyl-1,2-dihydro-3H-pyrazol-3-one, 54.**

Prepared according to General Procedure **H** on a 0.5 mmol scale using compound **54** (45 mg, 0.20 mmol, 1.0 eq.), sodium azide (13 mg, 0.22 mmol, 1.1 eq.), and ammonium chloride (21 mg, 0.4 mmol, 2.0 eq.) in DMF (0.4 mL). After 16 h at 110 °C, the reaction was complete by LCMS. Following reversed phase flash C18 column chromatography (0–60% MeCN in H_2_O), the desired product was isolated as a white powder (44 mg, 81%).

**^1^H NMR (500 MHz, DMSO-*d*_6_)** δ 7.74 – 7.69 (m, 2H), 7.47 – 7.40 (m, 2H), 7.24 – 7.17 (m, 1H), 3.06 (t, *J* = 7.4 Hz, 2H), 2.69 (t, *J* = 7.4 Hz, 2H), 1.99 (s, 3H).

**^13^C NMR (126 MHz, DMSO-*d*_6_)** δ155.5, 147.5, 137.7, 128.9, 124.8, 119.3, 102.3, 22.9, 20.2, 11.1.*

**HRMS (DART+)** Exact mass calcd. for C_12_H_12_N_3_O [M+H]^+^ = 271.13019; found 271.13022.

**Analytical note: in the ^13^C NMR spectrum, the pyrazolone carbonyl-bearing carbon was not observed.*

**2-(1-phenyl-1H-pyrazol-4-yl)pyrimidine-5-carbonitrile, 55.**

Prepared according to General Procedure **A** on a 1.5 mmol scale using compound **1-CN** (430 mg, 1.5 mmol, 1.5 eq.), DMF (3.0 mL), amidine **6** (280 mg, 1.5 mmol, 1.0 eq.), and *N*-methylmorpholine (0.41 mL, 3.8 mmol, 2.5 eq.) at 70 °C. After 3 h, the reaction was complete by LCMS and 10 mL water was added. The solids were filtered off (keep solids), washed with water (3x), and dried to isolate the desired product as a pale yellow solid (164 mg, 44%). No further purification was required.

**^1^H NMR (500 MHz, CDCl_3_)** δ 8.93 (s, 2H), 8.72 (d, *J* = 0.6 Hz, 1H), 8.43 (d, *J* = 0.6 Hz, 1H), 7.81 – 7.75 (m, 2H), 7.55 – 7.47 (m, 2H), 7.41 – 7.35 (m, 1H).

**^13^C NMR (126 MHz, CDCl_3_)** δ 162.8, 160.0, 142.2, 139.6, 129.8, 129.4, 127.8, 123.7, 119.7, 115.2, 105.8.

**HRMS (ESI+)** Exact mass calcd. for C_14_H_10_N_5_ [M+H]^+^ = 248.09307; found 248.09286.

***N*'-hydroxy-2-(1-phenyl-1H-pyrazol-4-yl)pyrimidine-5-carboximidamide, 56.**

****Prepared according to General Procedure **B** on a 0.60 mmol scale using compound **55** (148 mg, 0.60 mmol, 1.0 eq.), hydroxylamine hydrochloride (62 mg, 0.90 mmol, 1.5 eq.), and Et_3_N (0.13 mL, 0.90 mmol, 1.5 eq.) in EtOH (1.5 mL). After 1 h at rt, the reaction was complete by LCMS and the desired product was precipitated from H_2_O, filtered off, and dried to afford the desired product as a white solid (69 mg, 41%).

**^1^H NMR (500 MHz, DMSO-*d*_6_)** δ 10.01 (s, 1H), 9.30 (d, *J* = 0.6 Hz, 1 NH), 9.28 (s, 1H), 9.21 – 9.17 (s, 1 NH), 9.03 (s, 1H), 8.44 (d, *J* = 0.6 Hz, 1H), 8.37 (d, *J* = 0.6 Hz, 1H), 8.01 – 7.97 (m, 2H), 7.59 – 7.49 (m, 2H), 7.43 – 7.30 (m, 1H), 6.14 (s, 1OH).*

**^13^C NMR (126 MHz, DMSO-*d*_6_)** δ 161.5, 160.8, 160.0, 154.1, 147.0, 141.5, 140.8, 139.3, 139.0, 129.9, 129.7, 129.6, 128.4, 127.3, 126.9, 124.2, 124.0, 123.4, 119.0, 118.7, 115.7, 105.4.*

**HRMS (ESI+)** Exact mass calcd. for C_14_H_13_N_6_O [M+H]^+^ = 281.11484; found 281.11454.

**Analysis notes: Obtained as a ~1:1 mixture of E:Z isomers. The two pyrazole protons underintegrated due to the highly quadrupolar environment and the two pyridine hydrogen atoms are not chemically equivalent.*

**(3-(2-(1-phenyl-1H-pyrazol-4-yl)pyrimidin-5-yl)-1,2,4-oxadiazol-5-yl)methanamine, 57.**

****Prepared according to General Procedure **E** on a 0.1 mmol scale using compound **56** (28 mg, 0.10 mmol, 1.0 eq.), HCTU (46 mg, 0.11 mmol, 1.1 eq.), Boc-Gly-OH (35 mg, 0.1 mmol, 1.0 eq.) and DIPEA (35 μL, 0.10 mmol, 1.0 eq.) in DMF (0.5 mL). After 1 h at 110 °C, the reaction was complete by LCMS. The crude reaction mixture was subject to normal phase flash column chromatography on silica gel (0-30% *i*PrOH in DCM) to afford the desired product as a pale orange solid (29.4 mg, 70%).

**^1^H NMR (500 MHz, DMSO-*d*_6_)** δ 9.61 (m, 1H), 9.30 (m, 1H), 9.28 (s, 2H), 8.44 (d, *J* = 1.8 Hz, 1H), 8.05 – 7.96 (m, 2H), 7.58 – 7.50 (m, 2H), 7.42 – 7.37 (m, 1H), 3.32 (s, 2H).*

**^13^C NMR (126 MHz, DMSO-*d*_6_)** δ 161.5, 160.8, 141.5, 139.0, 129.9, 129.7, 127.3, 123.4, 119.0, 118.8, 115.7, 105.4, 28.1.*

**HRMS (DART)** Exact mass calcd. for C_21_H_22_N_7_O_3_ [M+H]^+^ = 420.17786; found 420.17798.

**Analysis note: Boc deprotection occurred in solution.*

**2-(6-phenylpyridin-3-yl)pyrimidine-5-carbonitrile, 58.**

Prepared according to General Procedure **A** on a 0.3 mmol scale using compound **1-CN** (89 mg, 0.3 mmol, 1.0 eq.), DMF (0.43 mL), amidine **9** (59 mg, 0.3 mmol, 1.0 eq.), and *N*-methylmorpholine (75 μL, 0.69 mmol, 2.3 eq.) at 70 °C. After 3 h, the reaction was complete by LCMS and 5 mL of water was added, upon which precipitation was observed. The product was filtered off, washed with H_2_O (3x), and dried to afford the desired product as a white solid (44 mg, 57%).

**^1^H NMR (600 MHz, DMF-*d*_7_, 353 K)** δ 9.69 (d, *J* = 2.3 Hz, 1H), 9.40 (s, 2H), 8.83 (dd, *J* = 8.4, 2.3 Hz, 1H), 8.28 – 8.23 (m, 3H), 8.19 (d, *J* = 8.3 Hz, 1H), 7.56 (dd, *J* = 8.2, 6.5 Hz, 3H), 7.52 (d, *J* = 7.0 Hz, 1H).

**^13^C NMR (151 MHz, DMF-*d*_7_, 353 K)** δ 164.3, 161.0, 159.8, 150.3, 138.6, 137.2, 130.6, 130.0, 129.0, 127.4, 120.3, 115.2, 107.9.

**HRMS (DART+)** Exact mass calcd. for C_16_H_14_N_3_O [M+H]^+^ = 259.09782; found 259.09720.

**2-(6-phenylpyridin-3-yl)pyrimidine-5-carbothioamide, 59.**

****Prepared according to General Procedure **D** on a 0.15 mmol scale using compound **58** (39 mg, 0.15 mmol, 1.0 eq.), sodium hydrogen sulfide monohydrate (19 mg, 0.26 mmol, 1.75 eq.) and magnesium chloride (14 mg, 0.15 mmol, 1.0 eq.) in DMF (0.45 mL). After 3 h at 60 °C, the reaction was complete by LCMS and 5 mL of water was added, upon which precipitation was observed. The product was filtered off, washed with H_2_O (3x), and dried to afford the desired product as a yellow solid (38 mg, 87%).

**^1^H NMR (500 MHz, DMSO-*d*_6_)** δ 10.28 (brs, 1NH_2_), 9.94 (brs, 1NH_2_), 9.63 (dd, *J* = 2.3, 0.9 Hz, 1H), 9.31 (s, 2H), 8.79 (dd, *J* = 8.4, 2.3 Hz, 1H), 8.23 – 8.16 (m, 3H), 7.57 – 7.52 (m, 2H), 7.52 – 7.48 (m, 1H).

**^13^C NMR (126 MHz, DMSO-*d*_6_**) δ 194.5, 162.8, 158.2, 155.9, 149.3, 137.8, 136.6, 130.9, 130.5, 129.8, 128.9, 126.9, 120.3.

**HRMS (ESI+)** Exact mass calcd. for C_16_H_14_N_3_O [M+H]^+^ = 293.0855; found 293.0858.

**ethyl 2-(2-(6-phenylpyridin-3-yl)pyrimidin-5-yl)thiazole-4-carboxylate, 60.**

****Prepared according to General Procedure **G** on a 0.13 mmol scale using compound **59** (38 mg, 0.13 mmol, 1.0 eq.) and ethyl bromopyruvate (20 μL, 0.16 mmol, 1.2 eq.) in EtOH (0.54 mL). After 16 h at 70 °C, the reaction was complete by LCMS and 5 mL of water was added, upon which precipitation was observed. The product was filtered off, washed with EtOH (3x), and dried to afford the desired product as a off-white solid solid (47.6 mg, 94%).

**^1^H NMR (500 MHz, THF-*d*_8_)** δ 9.76 (dd, *J* = 2.3, 0.8 Hz, 1H), 9.46 (s, 2H), 8.86 (dd, *J* = 8.4, 2.3 Hz, 1H), 8.48 (s, 1H), 8.27 – 8.19 (m, 2H), 8.04 (dd, *J* = 8.4, 0.9 Hz, 1H), 7.50 – 7.44 (m, 3H), 7.44 – 7.39 (m, 1H), 4.39 (q, *J* = 7.1 Hz, 2H), 1.39 (t, *J* = 7.1 Hz, 3H).

**^13^C NMR (126 MHz, THF-*d*_8_**) δ 164.7*, 163.1*, 161.5*, 160.0*, 156.0, 150.0, 139.8*, 137.1, 131.9, 130.3, 129.5, 129.3, 127.9, 126.5, 120.3, 61.7, 14.7.

**HRMS (ESI+)** Exact mass calcd. for C_16_H_14_N_3_O [M+H]^+^ = 389.1067; found 389.1067.

**Analysis notes: For highlighted carbons, the ^13^C assignments of unresolved peaks in the NMR spectrum were made by the available ^1^H-^13^C HSQC and ^1^H-^13^C HMBC cross-peaks. The ester carbonyl-carbon was not observed.*

**2-(4-methyl-6-oxo-2'-phenyl-1,6-dihydro-[2,5'-bipyrimidin]-5-yl)acetonitrile, 61.**

****Prepared according to General Procedure **A** on a 0.67 mmol scale with a slight modification using amidine **9** (165.0 mg, 0.6782mmol, 1.0 eq.), ethyl 3-oxobutanoate (171.7 mg, 1.015 mmol, 1.5 eq.) and *DBU replacing NMM* (0.20 mL, 0.13 mmol, 2 eq.) in DMF (3.4 mL), at 70 °C. After 18 h, water (~3 mL) was added. The solids were filtered off (keep solids), washed with water (3x), and dried to isolate the desired product as a beige solid (83.5 mg, 41%)

**^1^H NMR (500 MHz, DMSO-*d6*)** δ 13.14 (s, 1H), 9.31 (d, *J* = 2.4 Hz, 1H), 8.52 (dd, *J* = 8.4, 2.4 Hz, 1H), 8.21 – 8.13 (m, 3H), 7.58 – 7.48 (m, 3H), 3.79 (s, 2H), 2.43 (s, 3H).

**^13^C NMR (126 MHz, CDCl_3_)** δ 162.0^‡^, 158.7, 149.3, 138.0, 136.9, 130.4, 129.4, 127.4, 120.2, 118.3, 113.0^‡^, 109.0^‡^, 21.5^†^, 14.0.

**HRMS (DART+)** Exact mass calcd. for C_18_H_15_N_4_O [M+H]^+^ = 303.12404; found 303.12449.

**Analysis notes: 13C chemical shifts were extracted from 1D, HSQC^†^ and HMBC^‡^ spectra. Some signals were not observed.*

**2-(4-methyl-6-oxo-2'-phenyl-1,6-dihydro-[2,5'-bipyrimidin]-5-yl)acetonitrile, 62.**

****Prepared according to General Procedure **H** on a 0.11 mmol scale using compound **61** (33.4 mg, 0.11 mmol, 1.0 eq.), sodium azide (19.4 mg, 0.30 mmol, 2.7 eq.) and ammonium chloride (18.1 mg, 0.34 mmol, 3.0 eq.), in DMF (1.0 mL). After 18 h at 110 °C, the reaction was complete by LCMS. Following reverse phase flash C18 column chromatography (10–90% MeCN in H_2_O), the desired product was isolated as a white powder (27.1 mg, 71%).

**^1^H NMR (500 MHz, DMSO-*d*_6_)** δ 13.01 (br s, 1NH), 9.33 (d, *J* = 2.4 Hz, 1H), 8.53 (dd, *J* = 8.4, 2.4 Hz, 1H), 8.21 – 8.13 (m, 3H), 7.58 – 7.46 (m, 3H), 4.14 (s, 2H), 2.40 (s, 3H).

**^13^C NMR (126 MHz, CDCl_3_)** δ ^13^C NMR (126 MHz, dmso) δ 162.3^‡^, 161.9^‡^, 158.1, 154.3^‡^, 148.7, 137.6, 136.3, 129.9, 128.9, 126.9, 119.8, 116.9^‡^, 21.7, 20.0.*

**HRMS (ESI+)** Exact mass calcd. for C_18_H_16_N_7_O [M+H]^+^ = 346.1411; found 346.1415

**Analysis notes: 13C chemical shifts were extracted from 1D and HMBC^‡^ spectra. Some signals were not observed.*

**Glycyl-L-phenylalanine benzyl ester hydrochloride, 63.**

H-Phe-OBn•HCl (581 mg, 2.0 mmol, 1.0 eq.)* and Boc-Gly-OH (698 mg, 4.0 mmol, 2.0 eq.) were added to a sample vial fitted with a stir bar and dissolved in DCM (4.0 mL, 0.5 M). DIPEA (0.70 mL, 4.0 mmol, 2.0 eq.) was added, then the reaction mixture was cooled to 0 °C. EDC•HCl (570 mg, 3.0 mmol, 1.5 eq.) was added and the reaction mixture was stirred at 0 °C for 15 min. The reaction mixture was warmed to rt and stir for 5 h. The reaction mixture was diluted with EtOAc (50 mL) then washed with 0.1 M HCl (2 x 50 mL), sat. aq. NaHCO_3_ (2 x 50 mL), and brine (50 mL). The collected organics were dried (NaSO_4_) and concentrated under reduced pressure to give **Boc-Gly-Phe-OBn** as a pale yellow oil, which was used without further purification. The crude material was dissolved in HFIP (10 mL, 0.2 M), and conc. aq. HCl (20 drops) was added. The reaction mixture was vigorously stirred for 2 hours, then the reaction was concentrated under a flow of nitrogen to give the desired product **63** as a light pink solid (555 mg, 79% yield over 2 steps).

**Synthesis note: H-Phe-OBn•HCl was prepared according to Sepelgy and coworkers in comparable scale and yield.^6^*

**^1^H NMR (500 MHz, DMSO-*d*_6_)** δ 9.12 (d, *J* = 7.6 Hz, 1NH), 8.25 (s, 3NH), 7.39 – 7.19 (m, 10H), 5.12 (d, *J* = 12.5 Hz, 1H), 5.07 (d, *J* = 12.5 Hz, 1H), 4.60 (ddd, *J* = 8.6, 7.5, 6.0 Hz, 1H), 3.54 (q, *J* = 16.9 Hz, 2H), 3.07 (dd, *J* = 13.7, 6.0 Hz, 1H), 2.97 (dd, *J* = 13.8, 8.6 Hz, 1H).

**^13^C NMR (100 MHz, DMSO-*d*_6_)** δ 170.9, 166.1, 136.7, 135.6, 129.2, 128.4, 128.4, 128.1, 127.9, 126.7, 66.2, 54.0, 39.9, 36.7.

**HRMS (ESI+)** Exact mass calcd. for C_18_H_20_N_2_O_3_ [M+H]^+^ = 313.1547; found 313.1551.

**(S)-amino((2-((1-(benzyloxy)-1-oxo-3-phenylpropan-2-yl)amino)-2-oxoethyl)amino)methaniminium formate salt, 64.**

To a sample vial of compound **63** (524.8 mg, 1.5 mmol, 1.0 eq.) fitted with a stir bar was added 1*H*-pyrazole-carboxamidine hydrochloride (i.e., praxidine, 276 mg, 1.9 mmol, 1.25 eq.) and MeCN (7.5 mL, 0.2 M). DIPEA (0.70 mL, 3.8 mmol, 2.5 eq.) was added and the reaction was stirred overnight. The reaction mixture was concentrated under reduced pressure and subject to reverse phase flash C18 column chromatography (5-45% MeCN in H_2_O) to afford the desired product as a yellow oil that solidified into a yellow solid after standing overnight (298 mg, 49%).

**^1^H NMR (500 MHz, DMSO-*d*_6_)** δ 8.79 (d, *J* = 7.5 Hz, 1NH), 8.43 (s, NH), 8.31 (s, 1NH), 7.98 (s, 3NH), 7.40 – 7.14 (m, 10H), 5.08 (dd, *J* = 20.7, 12.4 Hz, 2H), 4.56 (dd, *J* = 14.4, 7.6 Hz, 1H), 3.82 (s, 2H), 3.05 (dd, *J* = 13.7, 6.2 Hz, 1H), 2.97 (dd, *J* = 13.8, 8.4 Hz, 1H).

**^13^C NMR (100 MHz, DMSO-*d*_6_)** δ 171.1, 167.9, 167.1, 158.1, 136.8, 135.6, 129.1, 128.4, 128.3, 128.1, 127.9, 126.7, 66.1, 54.0, 43.1, 36.8.

**HRMS (ESI+)** Exact mass calcd. for C_19_H_22_N_4_O_3_ [M+H]^+^ = 355.1765; found 355.1772.

**benzyl (5-cyanopyrimidin-2-yl)glycyl-L-phenylalaninate, 65.**

Prepared according to General Procedure **A** on a 0.13 mmol scale using **1-CN** (60 mg, 0.20 mmol, 1.5 eq.), DMF (0.40 mL), guanidine compound **64** (54 mg, 0.13 mmol, 1.0 eq.), and *N*-methylmorpholine (40 μL, 0.34 mmol, 2.5 eq.) at 70 °C. After stirring overnight, the reaction was cooled to rt and the neat reaction mixture subjected to reverse phase C18 column chromatography (5%-45% MeCN in H_2_O) to afford the desired product as a yellow solid (199 mg, 61%).

**^1^H NMR (500 MHz, DMSO-*d*_6_)** δ δ 8.68 (d, *J* = 3.0 Hz, 1H), 8.59 (d, *J* = 3.0 Hz, 1H), 8.44 (d, *J* = 7.7 Hz, 1NH), 8.37 (t, *J* = 6.3 Hz, 1NH), 7.45 – 7.10 (m, 10H), 5.08 (dd, *J* = 18.3, 12.6 Hz, 2H), 4.54 (td, *J* = 8.2, 5.8 Hz, 1H), 3.94 (ddd, *J* = 22.7, 16.8, 6.1 Hz, 2H), 3.04 (dd, *J* = 13.8, 5.9 Hz, 1H), 2.96 (dd, *J* = 13.8, 8.8 Hz, 1H).

**^13^C NMR (100 MHz, DMSO-*d*_6_)** δ 171.3, 168.8, 161.8, 161.4, 161.3, 137.0, 135.7, 129.1, 128.4, 128.3, 128.0, 127.8, 126.5, 117.0, 95.8, 66.0, 53.7, 43.6, 36.6.

**HRMS (ESI+)** Exact mass calcd. for C_23_H_21_N_5_O_3_ [M+H]^+^ = 416.1717; found 416.1721.

**benzyl (Z)-(5-(N'-hydroxycarbamimidoyl)pyrimidin-2-yl)glycyl-L-phenylalaninate, 66.**

Prepared according to General Procedure **B** on a 0.45 mmol scale using compound **65** (189 mg, 0.45 mmol, 1.0 eq.), hydroxylamine hydrochloride (47 mg, 0.68 mmol, 1.5 eq.) and Et_3_N (0.10 mL, 0.68 mmol, 1.5 eq.) in EtOH (2.3 mL, 0.2 M). The reaction mixture was stirred at 70 °C overnight, then concentrated under reduced pressure. The neat reaction mixture subjected to reverse phase C18 column chromatography (5%-45% MeCN in H_2_O) to afford the desired product **66** as a white solid (87 mg, 43%).

**^1^H NMR (500 MHz, DMSO-*d*_6_)** δ 9.55 (s, 1OH), 8.51 (s, 2H), 8.29 (d, *J* = 7.7 Hz, 1NH), 7.48 (t, *J* = 6.2 Hz, 1NH), 7.40 – 7.05 (m, 10H), 5.85 (s, 2NH), 5.07 (dd, *J* = 17.3, 12.6 Hz, 2H), 4.54 (td, *J* = 8.1, 5.8 Hz, 1H), 3.89 (ddd, *J* = 23.2, 16.8, 6.5 Hz, 2H), 3.03 (dd, *J* = 13.7, 5.9 Hz, 1H), 2.96 (dd, *J* = 13.7, 8.4 Hz, 1H).

**^13^C NMR (100 MHz, DMSO-*d*_6_)** δ 171.3, 169.8, 162.1, 147.8, 136.9, 135.7, 129.1, 128.4, 128.2, 128.0, 127.9, 126.5, 116.5, 66.0, 53.6, 43.9, 36.7.

**HRMS (ESI+)** Exact mass calcd. for C_23_H_24_N_6_O_4_ [M+H]^+^ = 449.1932; found 449.1935.

**benzyl(5-(5-(((tert-butoxycarbonyl)amino)methyl)-1,2,4-oxadiazol-3-yl)pyrimidin-2-yl)glycyl-L-phenylalaninate, 67.**

Prepared according to General Procedure **E** on a 0.17 mmol scale using compound **66** (78 mg, 0.17 mmol, 1.0 eq.), HCTU (79 mg, 0.19 mmol, 1.1 eq.), Boc-Gly-OH (30 mg, 0.17 mmol, 1.0 eq.) and DIPEA (60 µL, 0.35 mmol, 2.0 eq.) in DMF (0.87 mL). at 110 °C, overnight. Once cooled the crude reaction mixture was subject to reverse phase C18 column chromatography (5-25%, 25-60% MeCN in H_2_O) to afford the desired product **67** as a brown solid (35 mg, 34%).

**^1^H NMR (500 MHz, CDCl_3_)** δ 8.85 (s, 2H), 7.39 – 7.30 (m, 3H), 7.26 (dd, *J* = 6.4, 3.2 Hz, 2H), 7.14 – 7.05 (m, 3H), 6.98 – 6.85 (m, 3H), 6.49 (s, 1NH), 5.54 (s, 0.7NH, major rotamer), 5.50 (s, 0.1NH, minor rotamer), 5.11 (q, *J* = 12.1 Hz, 2H), 4.96 (dt, *J* = 8.3, 5.9 Hz, 1H), 4.61 (d, *J* = 6.1 Hz, 1.6H, major rotamer), 4.53 (s, 0.2H, minor rotamer), 4.11 (dtd, *J* = 19.1, 16.7, 5.9 Hz, 2H), 3.09 (qd, *J* = 13.9, 5.9 Hz, 2H), 1.98 (s, 0.5H, minor rotamer), 1.46 (s, 8.5H, major rotamer).

**^13^C NMR (100 MHz, CDCl_3_)** δ 176.9, 171.4, 171.3, 169.3, 165.0, 162.7, 157.4, 155.7, 135.5, 135.5, 135.1, 130.2, 129.3, 128.7, 128.6, 128.6, 128.6, 127.1, 111.6, 80.9, 67.4, 53.2, 45.5, 37.8, 37.2, 28.4.

**HRMS (ESI+)** Exact mass calcd. for C_30_H_34_N_7_O_6_ [M+H]^+^ = 588.2565; found 588.2565.

**(S)-1-(((3-(2-((2-(((S)-1-(benzyloxy)-1-oxo-3-phenylpropan-2-yl)amino)-2-oxoethyl)amino)pyrimidin-5-yl)-1,2,4-oxadiazol-5-yl)methyl)amino)-1-oxopropan-2-aminium chloride, 68.**

To a sample vial containing compound **67** (23.6 mg, 40 µmol, 1.0 eq.) with a stir bar was added was added HFIP (0.80 mL, 50 mM) and conc. aq, HCl (2 drops). The reaction was stirred vigorously for 2 h, then concentrated under flow of N_2_. Boc-Ala-OH (15 mg, 80 µmol, 2.0 eq.) and 6-Cl-HOBt (10 mg, 80 µmol, 1.5 eq.) were added to the vial and dissolved in DCM (400 µL, 0.1 M). DIPEA (20 µL, 115 µmol, 2.9 eq.) was added, then the reaction mixture was cooled to 0 °C. EDC•HCl (12 mg, 60 µmol, 1.5 eq.) was added and the reaction mixture was stirred at 0 °C for 15 min. The reaction mixture was allowed to warm to room temperature and stir for 2 h. The reaction mixture was diluted with EtOAc (20 mL) and transferred to a sep. funnel. The organic layer was successively washed with 0.1 M HCl (2 x 20 mL), sat. aq. NaHCO_3_ (2 x 20 mL), and brine (20 mL). The organic layer was dried over NaSO_4_, decanted, and concentrated under reduced pressure. The crude material was dissolved in HFIP (0.80 mL, 50 mM), and conc. aq. HCl (2 drops) was added. The reaction mixture was vigorously stirred for 2 hours, then the reaction was concentrated under a flow of nitrogen. The crude product was subject to reverse phase C18 column chromatography (5-60% MeCN in H_2_O) to afford the desired product as a white solid (35 mg, 34%).

**^1^H NMR (500 MHz, CDCl_3_)** δ 8.92 – 8.62 (m, 3H), 8.39 (d, *J* = 7.7 Hz, 1NH), 8.01 (t, *J* = 6.3 Hz, 1NH), 7.39 – 7.12 (m, 10H), 5.08 (dd, *J* = 18.0, 12.7 Hz, 2H), 4.63 (s, 2H), 4.55 (ddd, *J* = 8.8, 7.6, 5.8 Hz, 1H), 3.94 (ddd, *J* = 23.1, 16.9, 6.4 Hz, 2H), 3.45 (q, *J* = 6.9 Hz, 1H), 3.04 (dd, *J* = 13.7, 5.8 Hz, 1H), 2.96 (dd, *J* = 13.7, 8.8 Hz, 1H), 1.21 (d, *J* = 6.9 Hz, 3H).

**^13^C NMR (100 MHz, CDCl_3_)** δ 177.5, 175.6, 171.3, 169.3, 164.6, 162.9, 156.8, 156.3, 137.0, 135.7, 129.1, 128.4, 128.2, 128.0, 127.8, 126.5, 109.7, 66.0, 53.6, 49.8, 43.8, 36.6, 35.2, 20.7.

**HRMS (ESI+)** Exact mass calcd. for C_28_H_30_N_8_O_5_ [M+H]^+^ = 559.2412; found 559.2418.

## **2.3 Controls for the stability of 2-Cl-Trt resin**

Two 2-Cl-Trt resin-loaded samples (10 mg each) of Fmoc-Gly-Phe-OH were placed in separate 0.5-dram vials, followed by the addition of 1 mL of DMF. One sample was heated at 80 °C for 6 h, while the other was kept at rt. Afterward, the DMF was drained, and the resin was washed twice with *i*PrOH, followed by two washes with DCM, then dried under a stream of nitrogen. Both samples were then subjected to cleavage under standard 20% HFIP/DCM washes, and the cleavage mixtures were evaporated under a stream of N_2_. The resulting residues were dissolved in 1 mL of 50% MeCN/H₂O and analyzed by LCMS. The UV trace integration was used to approximate the degree of thermal cleavage of Fmoc-Gly-Phe-OH from 2-Cl-resin.

## **2.4 Solid-phase peptide synthesis**

**Fmoc-Phe-Gly-Wang, 69.**

Wang resin (228 mg, 0.1 mmol) was preloaded with Fmoc-phenylalanine. Wang-Phe-Fmoc was swollen in DMF (5 mL/g resin) for 15 min. The solvent was then drained, and the resin was used directly in peptide elongation.

**General Procedure I: manual solid phase-peptide synthesis.**

**SPPS:** Fully protected resin-bound peptides were synthesized using manual Fmoc solid-phase peptide chemistry. All *N*-Fmoc amino acids were employed. The Fmoc protecting group was removed by treating the resin with 20% piperidine in DMF for 20 min. at rt with gentle shaker agitation. The solution was drained, and the resin was washed with DCM (5x) and DMF (5x). For amino acid coupling, the resin was treated with the respective Fmoc-protected amino acid (5 eq.), HCTU (5 eq.), and DIPEA (20% in DMF, 10 eq.) for 1 h. Completion of each coupling step was assessed by Kaiser test and micro-cleavage followed by LCMS analysis, and a repeat coupling was performed when necessary.

**Cleavage:** Once the linear hybrid molecule was assembled, the sequence was cleaved from Wang resin using TFA/H₂O, 95:5, v/v, at rt for 1 hour. The resin was then filtered, and the filtrate was evaporated under a stream of N_2_.

**methyl carbamimidoylglycyl-L-phenylalaninate-Wang, 70.**

The Fmoc protecting group was removed by treating the resin **69** with 20% piperidine in DMF for 20 min., twice, at rt with gentle agitation. After deprotection, 1H-pyrazole-1-carboxamidine hydrochloride (i.e., paraxidine, 73.3 mg, 0.50 mmol, 5.0 eq.) was dissolved in 1 mL of DMF, followed by the addition of DIPEA (10 μL, 0.6 mmol, 6.0 eq.). The resulting mixture was then added to the resin, and the reaction was allowed to proceed overnight at rt with gentle agitation. Completion of the reaction was monitored by micro-cleavage followed by LC-MS analysis.

**methyl (5-cyanopyrimidin-2-yl)glycyl-L-phenylalaninate-Wang, 71.**

DMF (1 mL) was added to a vial, followed by the resin **70**. NMM (44 μL, 0.40 mmol, 4.0 eq.) was then added to the vial, followed by the addition of **1-CN** (60 mg, 0.20 mmol, 2.0 eq.). The resulting mixture was heated to 70°C for 2 hours. Completion of the reaction was monitored by micro-cleavage followed by LCMS analysis.

**methyl (Z)-(5-(N'-hydroxycarbamimidoyl)pyrimidin-2-yl)glycyl-L-phenylalaninate-Wang, 72.**

A solution of hydroxylamine hydrochloride (69 mg, 1.0 mmol, 10 eq.) and DIPEA (210 μL, 1.2 mmol, 12 eq.) in DMF was added to a vial containing the resin. The reaction mixture was stirred at 70°C for 3 hours. The reaction was monitored by micro-cleavage followed by LC-MS analysis.

**methyl (5-(5-(((((9H-fluoren-9-yl)methoxy)carbonyl)amino)methyl)-1,2,4-oxadiazol-3-yl)pyrimidin-2-yl)glycyl-L-phenylalaninate-Wang, 73.**

General procedure **I** was first followed, with resin **72** and Fmoc-Gly-OH. The coupling step was monitored by micro-cleavage followed by LCMS analysis. Once the coupling reaction was complete, the resin was washed sequentially with DMF (×3), *i*PrOH (×1), and DCM (×3). Dry THF (0.1 M) was then added to the resin, followed by TBAF (1.0 M in THF, 200 μL, 2 eq.). The mixture was shaken for 2 h, and the reaction progress was monitored by micro-cleavage followed by LCMS analysis.

**(5-(5-(((S)-2-aminopropanamido)methyl)-1,2,4-oxadiazol-3-yl)pyrimidin-2-yl)glycyl-L-phenylalanine- TFA salt, 74.**

General procedure **I** was first followed with resin **73** and Fmoc-Ala-OH. Upon completion of the coupling reaction, the Fmoc protecting group was removed using general procedure **I** deprotection conditions (20% piperidine in DMF). The resulting peptide was cleaved from the resin using 95% trifluoroacetic acid (TFA)/H₂O (2×1 h). The combined cleavage solutions were concentrated under a stream of nitrogen. The resulting crude material was purified by reverse-phase HPLC (MeCN/H_2_O) to afford the desired compound as a white powder (13mg, 27% overall yield).

**^1^H NMR (500 MHz, DMSO-*d_6_*)**: δ 9.08 (s, 1H), 8.80-8.78 (m, 2H), 8.08 (t, *J* = 6.4 Hz, 1H), 7.75 (d, *J* = 7.4 Hz, 1H), 7.15-7.08 (m, 5H), 4.66 (s, 2H), 4.28-4.24 (m, 1H), 3.96 (dd, *J* = 16.7, 6.3 Hz, 1H), 3.86 (dd, *J* = 16.7, 6.2 Hz, 1H), 3.64 (q, *J* = 6.9 Hz, 1H), 3.02 (dd, *J* = 13.5, 5.0 Hz, 1H), 2.90 (dd, *J* = 13.5, 7.1 Hz, 1H), 1.27 (d, *J* = 7.0 Hz, 1H).

**^13^C NMR (126 MHz, DMSO-*d_6_*)**: δ 179.5, 177.8, 174.2, 173.2, 168.8, 165.1, 163.4, 157.2, 138.2, 129.8, 125.3, 110.2, 54.7, 49.6, 44.6, 37.8, 35.7, 19.8.

**^19^F NMR (377 MHz, DMSO-*d_6_*)**: δ 71.65 (s, TFA counterion).

**HRMS (ESI+)** Exact mass calcd. for C_19_H_21_N_3_O_3_ [M+H]^+^ = 469.1942; found 469.1934.

# **3. Copies of spectra for all novel compounds**

**(*Z*)-*N*-(2-cyano-3-(dimethylamino)allylidene)-N-methylmethanaminium hexafluorophosphate(V), 1-CN**

**^1^H NMR (500 MHz, MeCN-*d3*)**

**^13^C NMR (126 MHz, MeCN-*d3*)**

**2-phenylpyrimidine-5-carbonitrile, 4.**

**^1^H NMR (400 MHz, DMSO-*d*_6_)**


**^13^C NMR (100 MHz, DMSO-*d*_6_)**

***N*'-hydroxy-1-phenyl-1H-pyrazole-4-carboximidamide, 5.**

**^1^H NMR (500 MHz DMSO-*d*_6_)**

**
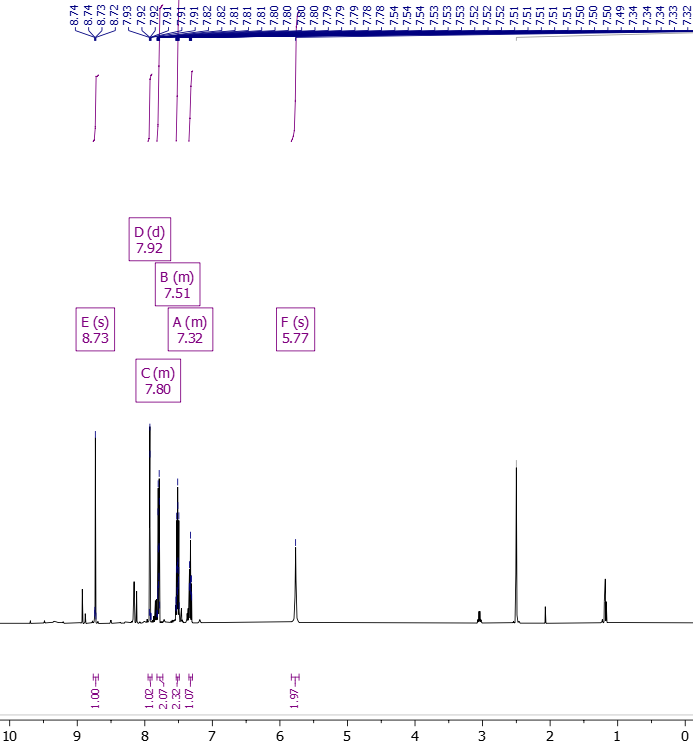
**

**^13^C NMR (126 MHz, DMSO-*d*_6_)**

**
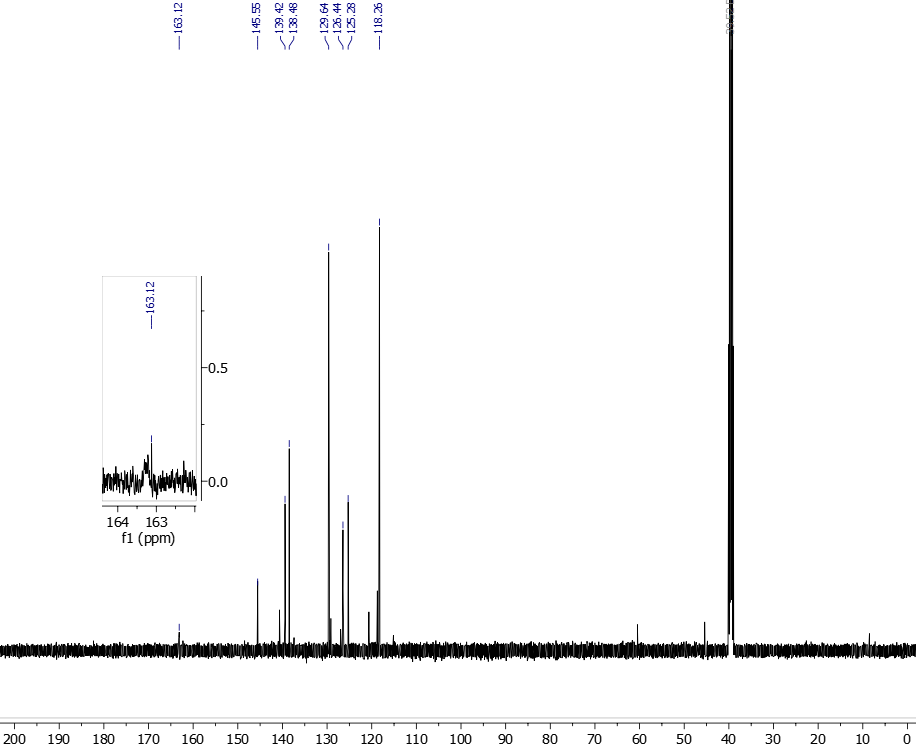
**

**1-phenyl-1H-pyrazole-4-carboximidamide, 6.**

**^1^H NMR (500 MHz, CDCl_3_)**


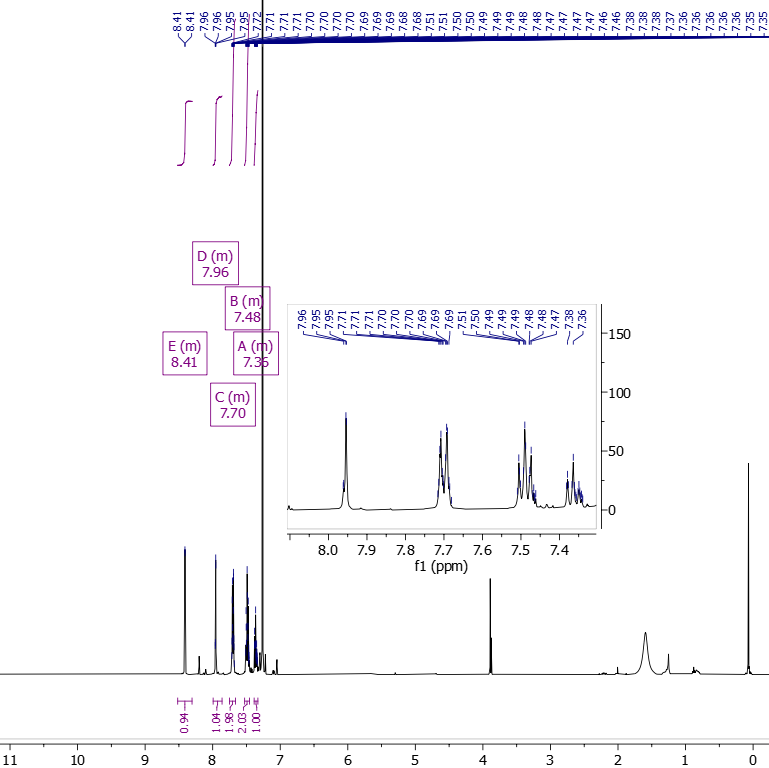


**^13^C NMR (126 MHz, CDCl_3_)**

**
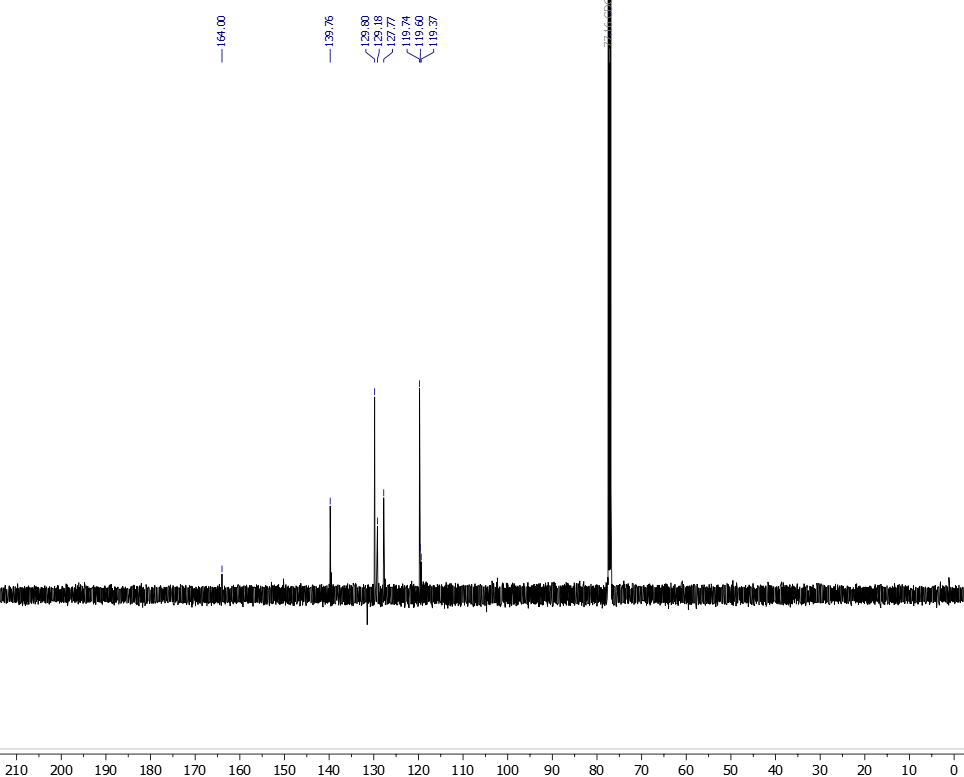
**

**1-phenyl-1H-pyrazole-4-carbothioamide, 7.**

**^1^H NMR (500 MHz, CDCl_3_)**


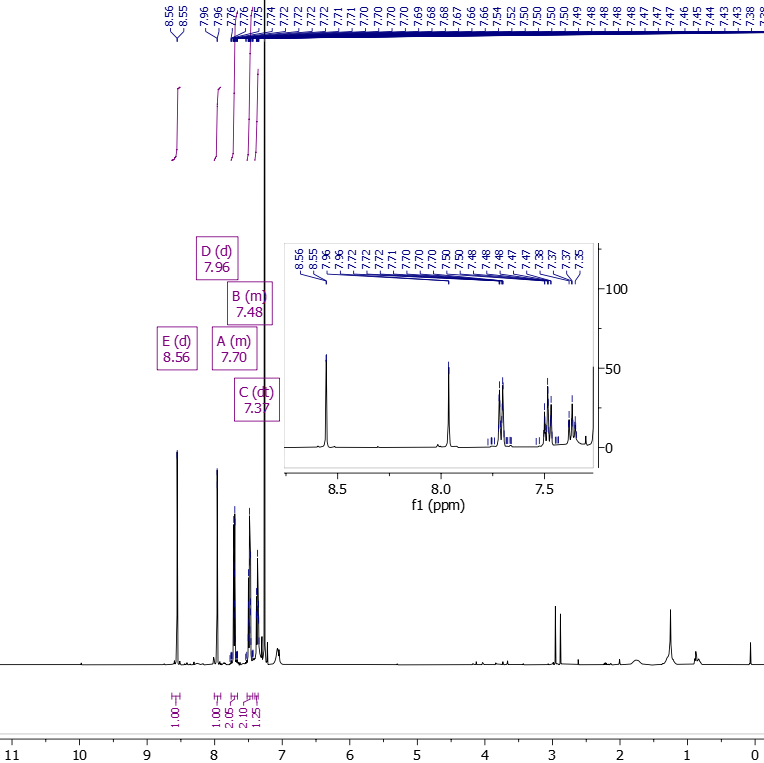


**^13^C NMR (126 MHz, CDCl_3_)**

**
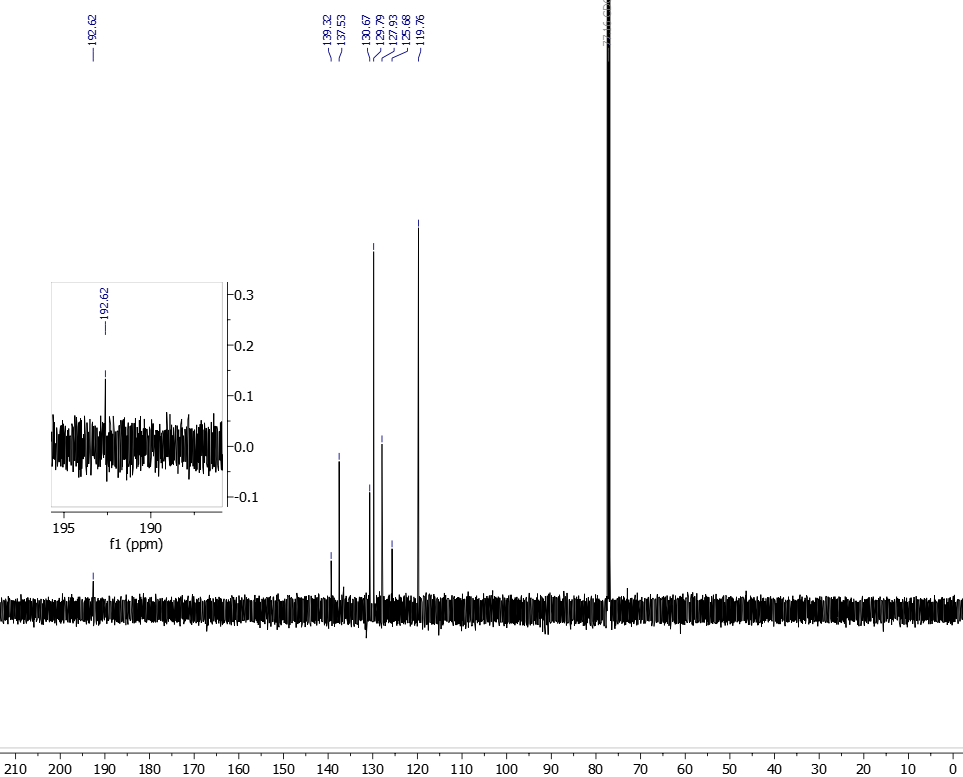
**

**N'-hydroxy-6-phenylnicotinimidamide, 8.**

**^1^H NMR (400 MHz, DMSO-*d*_6_)**

**
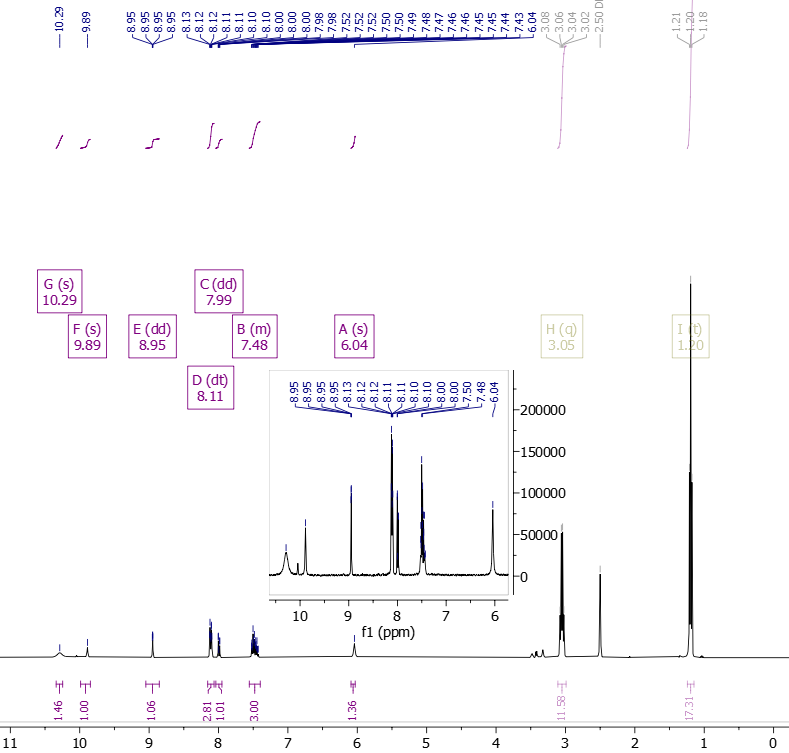
**

**^13^C NMR (126 MHz, DMSO-*d*_6_)**

**
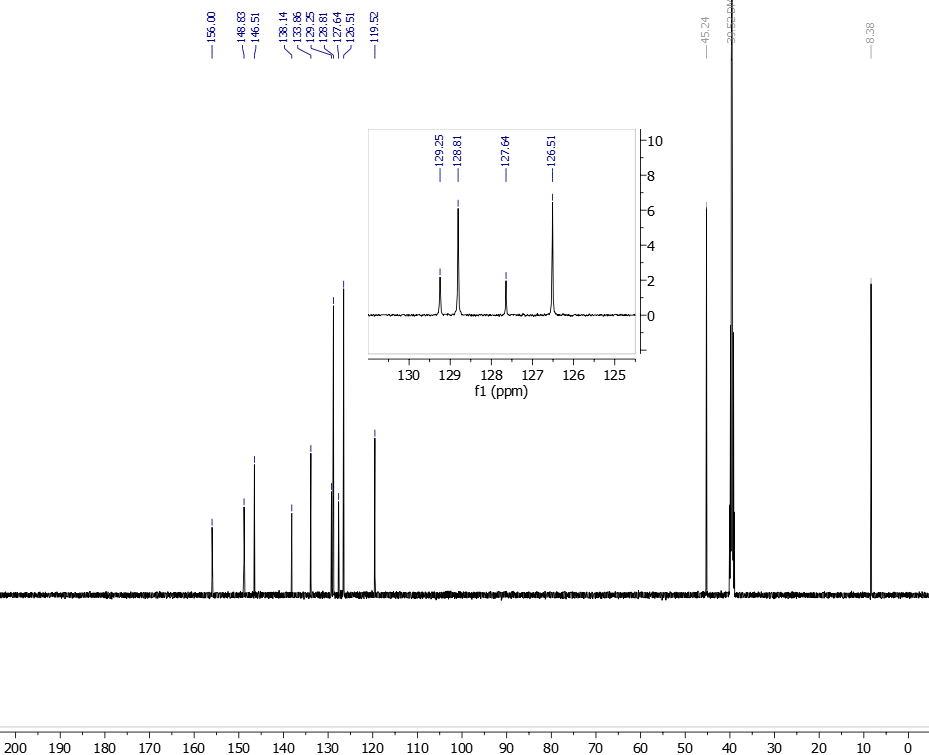
**

**6-phenylnicotinimidamide formic acid salt, 9.**

**^1^H NMR (400 MHz, DMSO-*d6*)**

**^13^C NMR (100 MHz, DMSO-*d6*)**

**6-phenylpyridine-3-carbothioamide, 10.**

**^1^H NMR (500 MHz, CDCl_3_)**


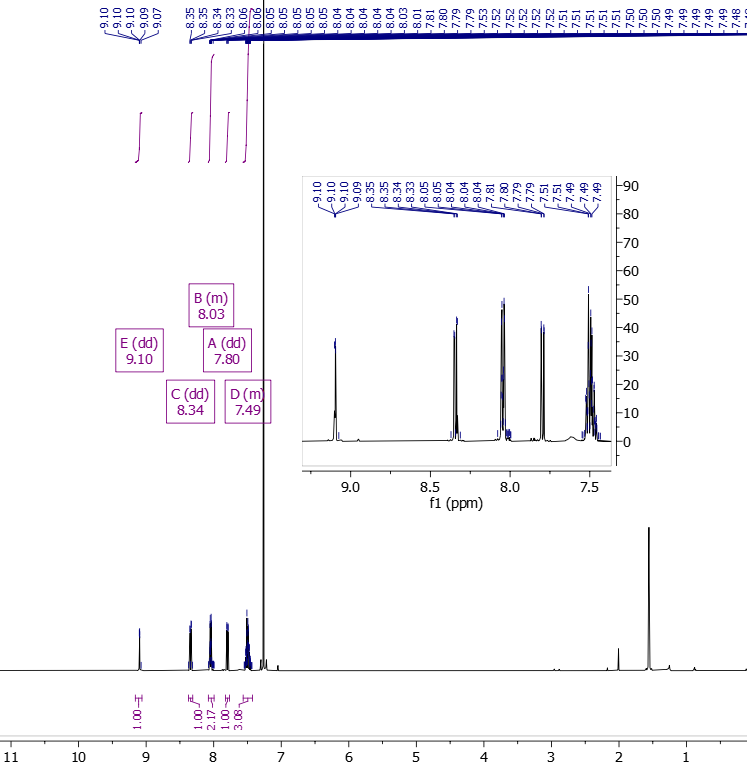


**^13^C NMR (126 MHz, DMSO-*d*_6_)**


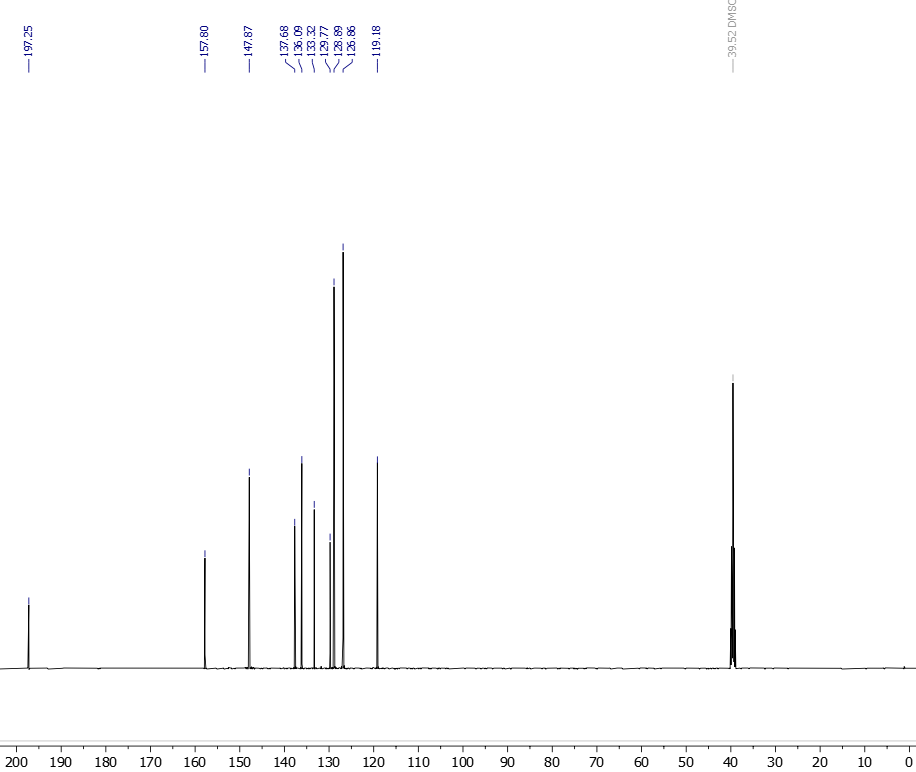


***N*'-hydroxy-2-phenylpyrimidine-5-carboximidamide, 11.**

**^1^H NMR (500 MHz, DMSO-*d*_6_)**

**^13^C NMR (126 MHz, DMSO-*d*_6_)**

**2-phenylpyrimidine-5-carboximidamide formic acid salt, 12.**

**^1^H NMR (500 MHz, DMSO-*d*_6_)**

**^13^C NMR (126 MHz, DMSO-*d*_6_)**

**2-phenylpyrimidine-5-carbothioamide, 13.**

**^1^H NMR (500 MHz, DMSO-*d*_6_)**

**^13^C NMR (126 MHz, DMSO-*d*_6_)**

***tert-*butyl ((3-(1-phenyl-1H-pyrazol-4-yl)-1,2,4-oxadiazol-5-yl)methyl)carbamate, 14.**

**^1^H NMR (500 MHz, DMSO-*d*_6_)**

**^13^C NMR (126 MHz, DMSO-*d*_6_)**

**5-chloro-2-(1-phenyl-1H-pyrazol-4-yl)pyrimidine, 15.**

**^1^H NMR (500 MHz, DMSO-*d*_6_)**


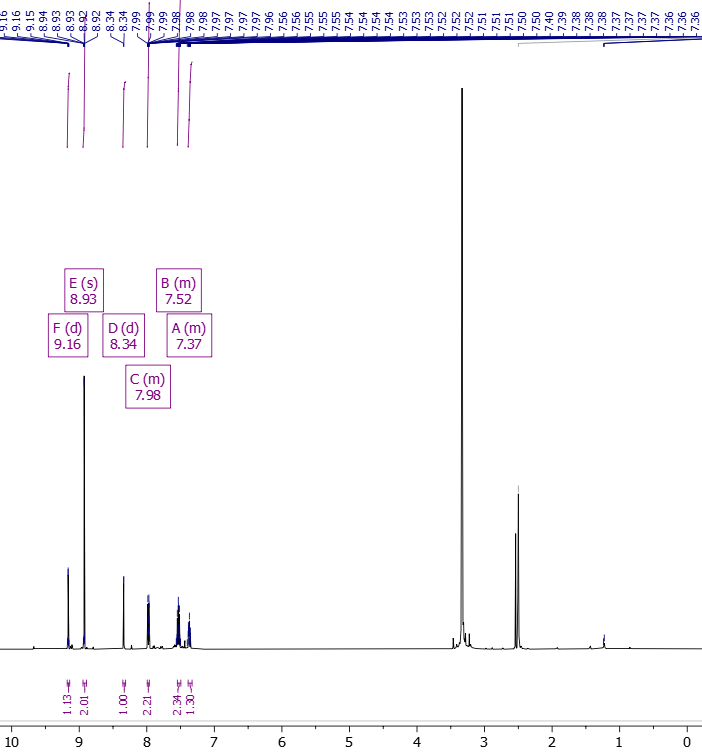


**^13^C NMR (126 MHz, DMSO-*d*_6_)**

**
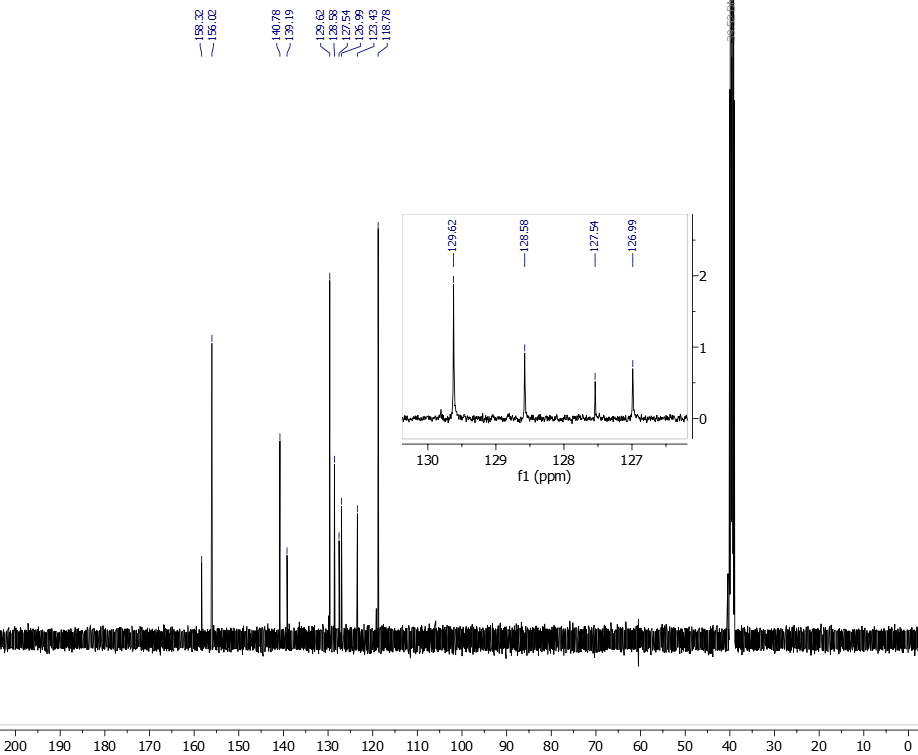
**

**6-methyl-2-(1-phenyl-1H-pyrazol-4-yl)pyrimidin-4-ol, 16.**

**^1^H NMR (500 MHz, DMSO-*d*_6_)**

**^
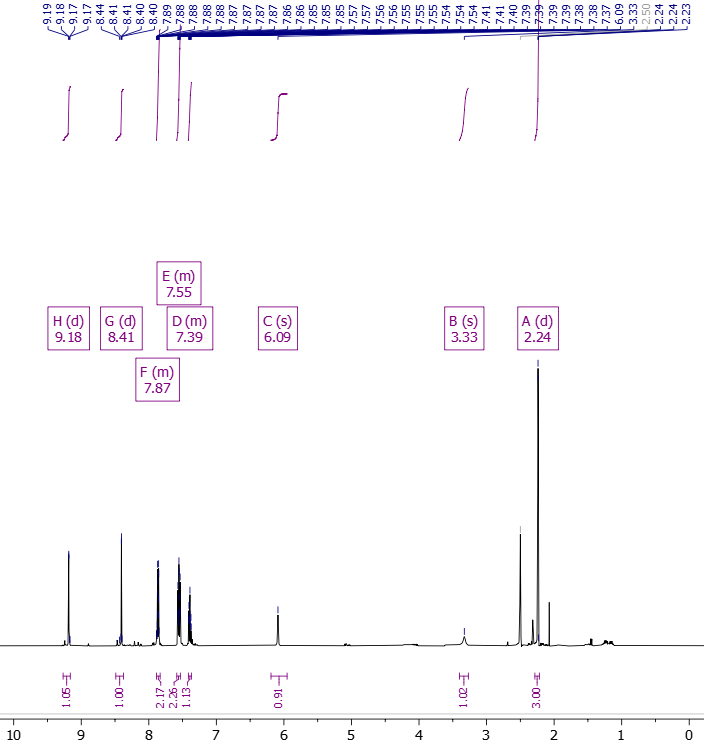
^**

**^13^C NMR (126 MHz, DMSO-*d*_6_)**

**
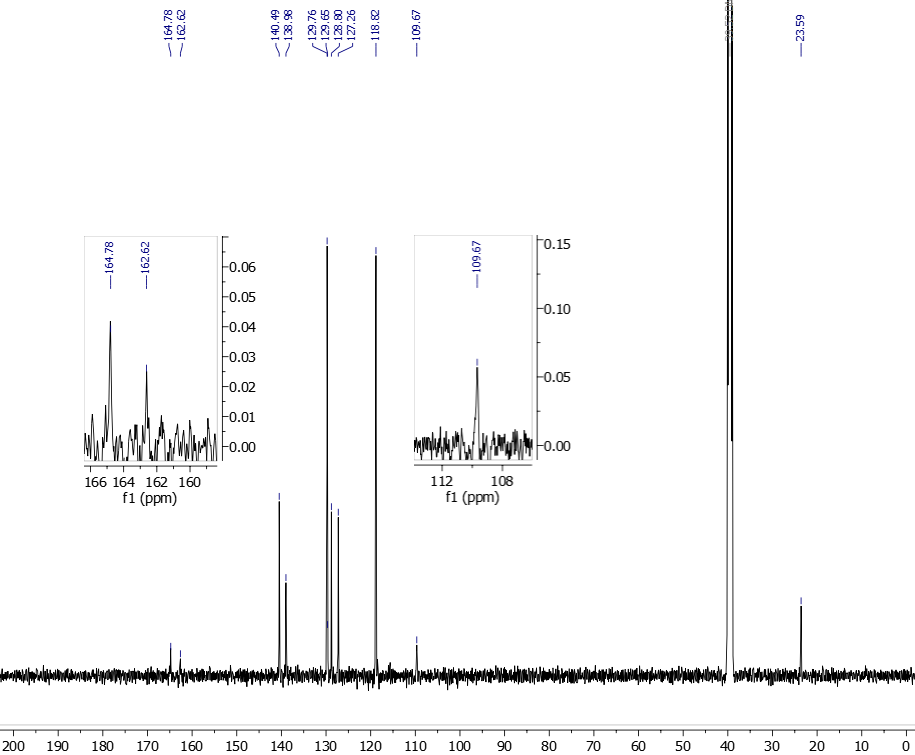
**

**ethyl 2-(1-phenyl-1H-pyrazol-4-yl)thiazole-4-carboxylate, 17.**

**^1^H NMR (500 MHz, CDCl_3_)**


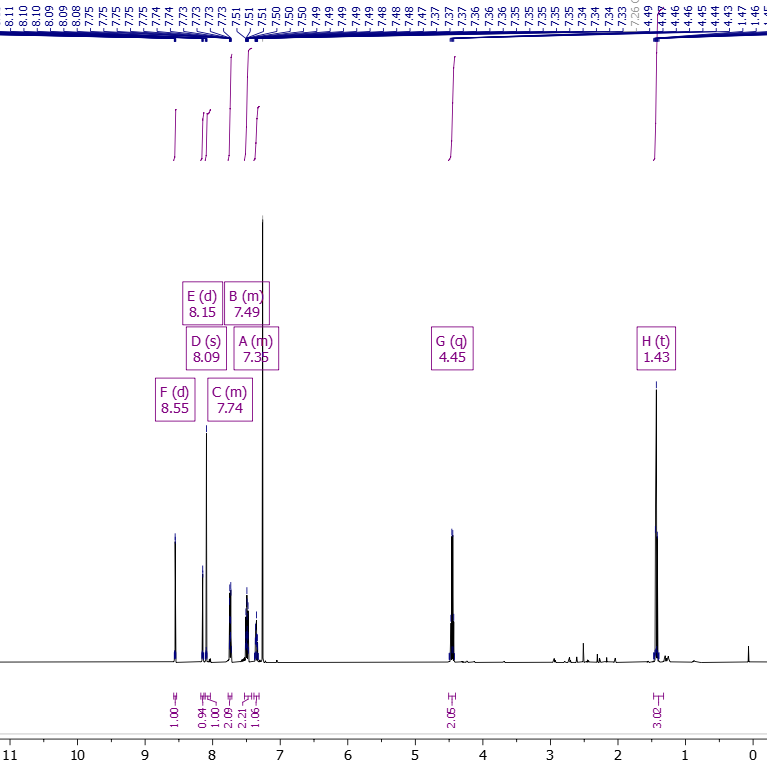


**^13^C NMR (126 MHz, CDCl_3_)**

**
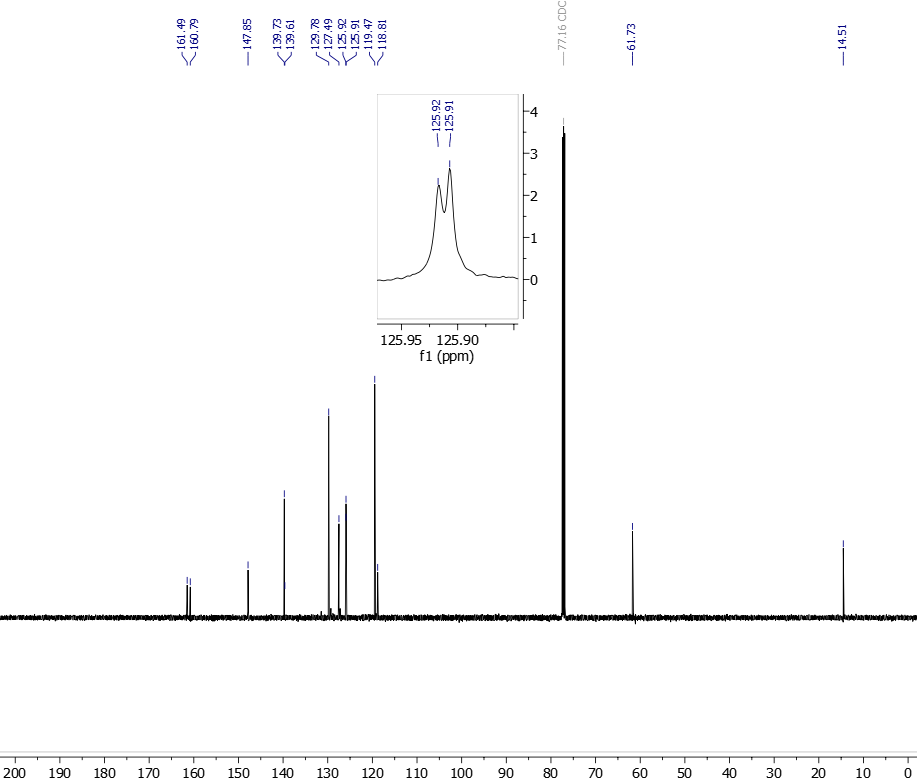
**

***tert*-butyl ((3-(6-phenylpyridin-3-yl)-1,2,4-oxadiazol-5-yl)methyl)carbamate, 18.**

**^1^H NMR (500 MHz, CDCl_3_)**


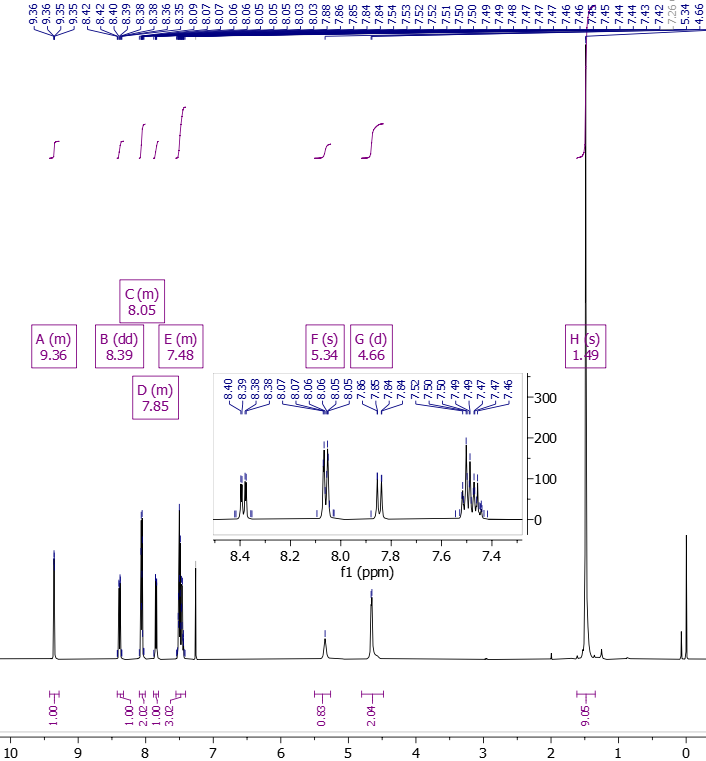


**^13^C NMR (126 MHz, CDCl_3_)**


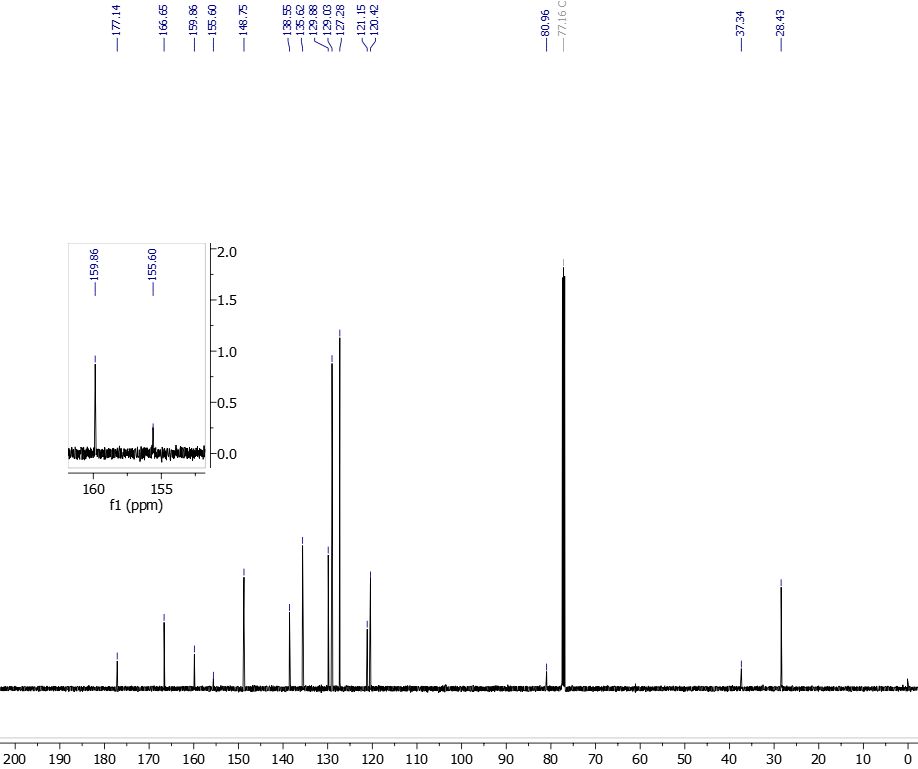


**5-chloro-2-(6-phenylpyridin-3-yl)pyrimidine, 19.**

**^1^H NMR (500 MHz, CDCl_3_)**


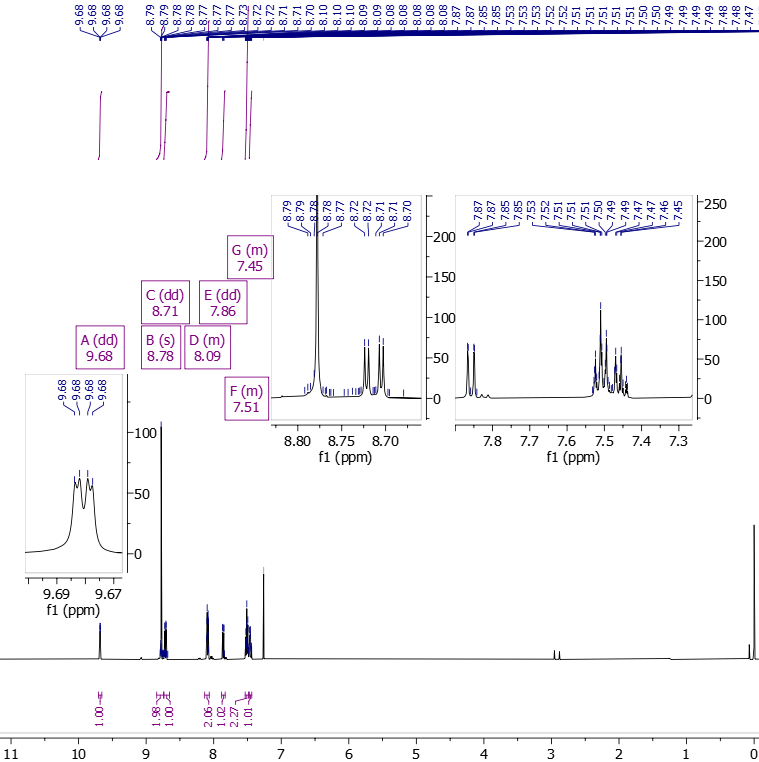


**^13^C NMR (126 MHz, CDCl_3_)**

**
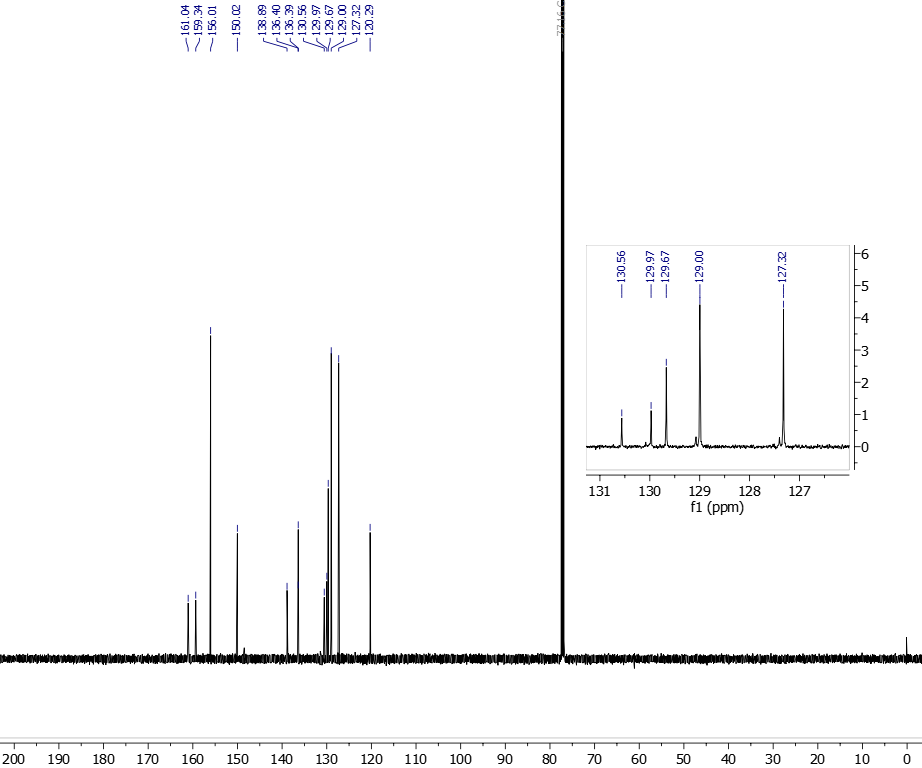
**

**6-methyl-2-(6-phenylpyridin-3-yl)pyrimidin-4(3H)-one, 20.**

**^13^C NMR (126 MHz, DMSO-*d*_6_)**

**
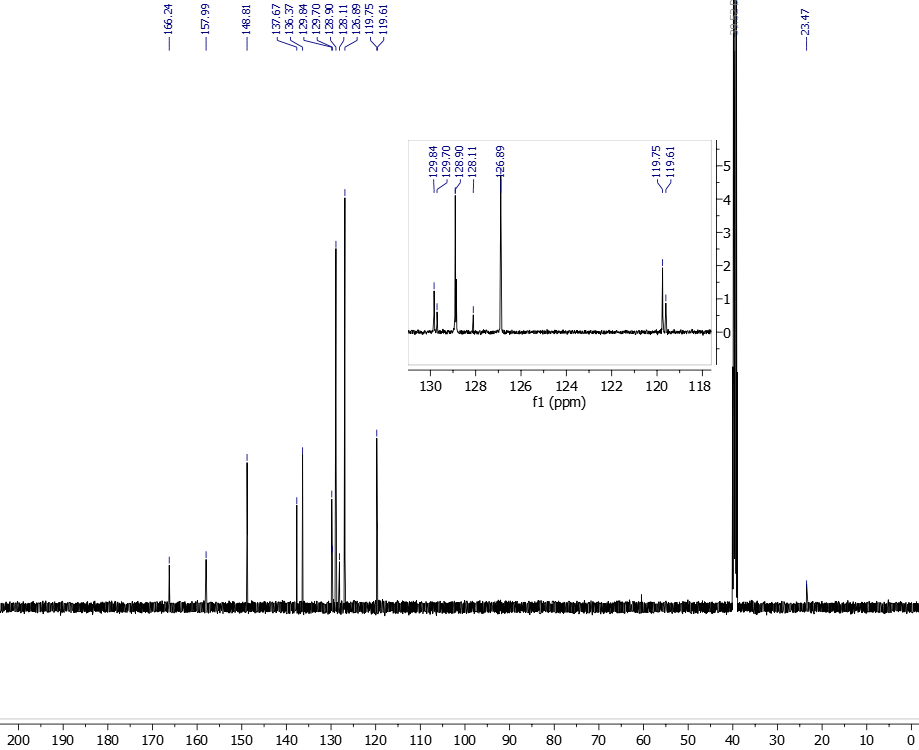
**

**ethyl 2-(6-phenylpyridin-3-yl)thiazole-4-carboxylate, 21.**

**^1^H NMR (500 MHz, CDCl_3_)**


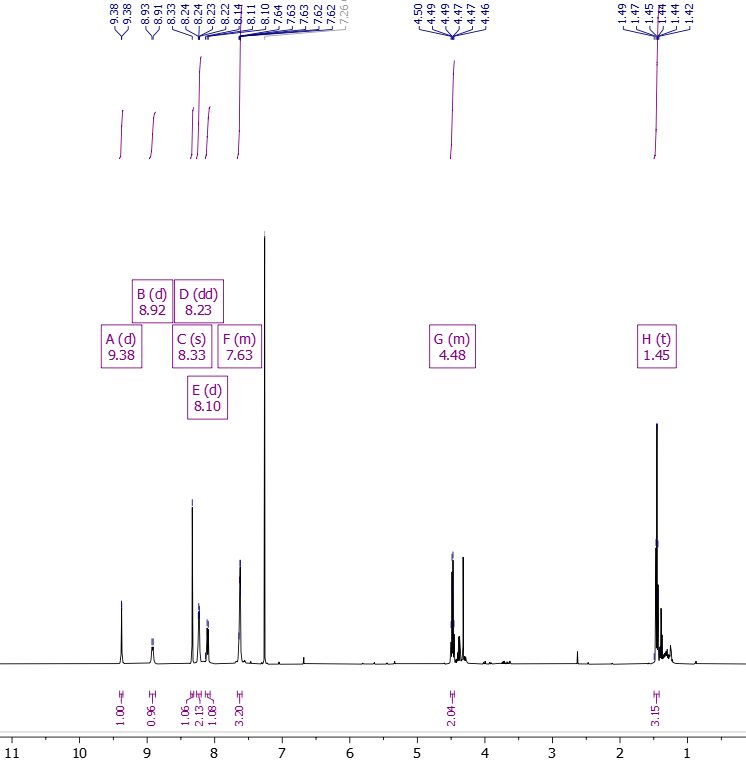


**^13^C NMR (126 MHz, CDCl_3_)**

**
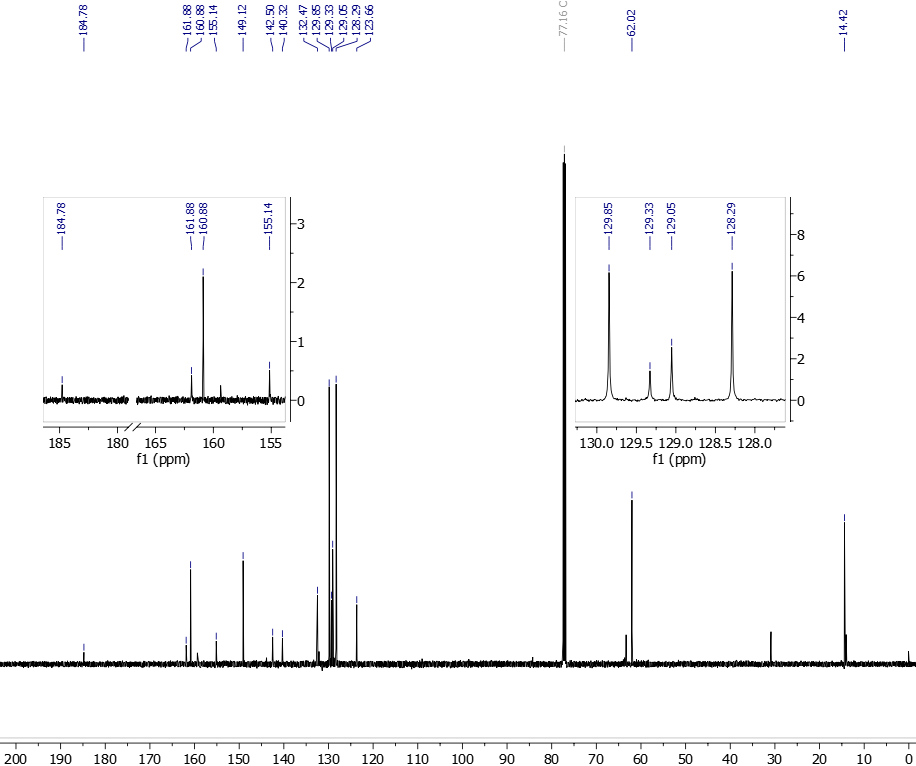
**

***tert*-butyl ((3-(2-phenylpyrimidin-5-yl)-1,2,4-oxadiazol-5-yl)methyl)carbamate, 22.**

**^1^H NMR (500 MHz, DMSO-*d*_6_)**

**^13^C NMR (126 MHz, DMSO-*d*_6_)**

**5-chloro-2'-phenyl-2,5'-bipyrimidine, 23.**

**^1^H NMR (700 MHz, PhMe-*d*_8_)**

**^1^H-^13^C HSQC (700 MHz, PhMe-*d*_8_)**

**^1^H-^13^C HMBC (700 MHz, PhMe-*d*_8_)**

**6-methyl-2'-phenyl-[2,5'-bipyrimidin]-4(3H)-one, 24.**

**^1^H NMR (500 MHz, DMSO-*d*_6_)**

**^13^C NMR (125 MHz, DMSO-*d*_6_)**

**^1^H-^13^C HMBC (400 MHz)**

**ethyl 2-(2-phenylpyrimidin-5-yl)thiazole-4-carboxylate , 25.**

**1H NMR (500 MHz, CDCl_3_)**

**^13^C NMR (126 MHz, CDCl_3_)**

**5-(1-phenyl-1H-pyrazol-4-yl)-1H-tetrazole, 26.**

**^1^H NMR (400 MHz, DMSO-*d*_6_)**


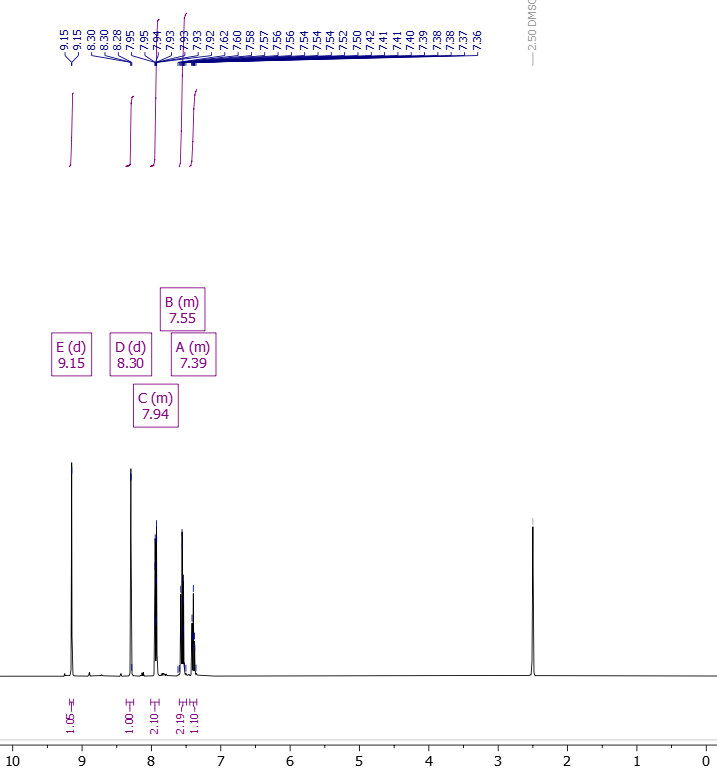


**^13^C NMR (101 MHz, DMSO-*d*_6_)**


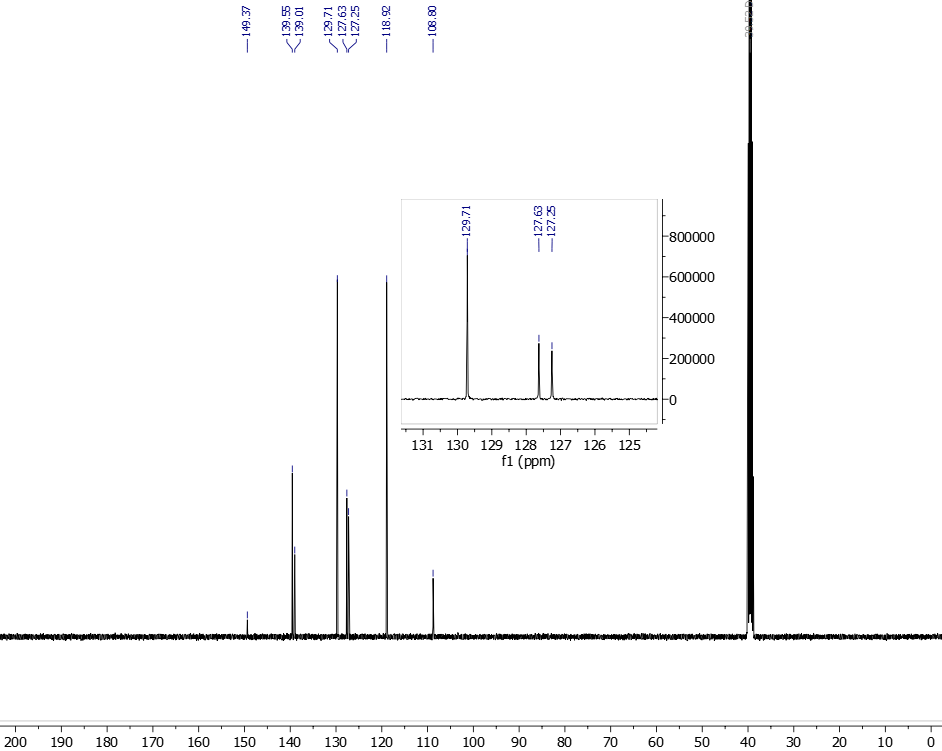


**2-phenyl-5-(1H-tetrazol-5-yl)pyridine, 27.**

**^1^H NMR (400 MHz, DMSO-*d*_6_)**

**
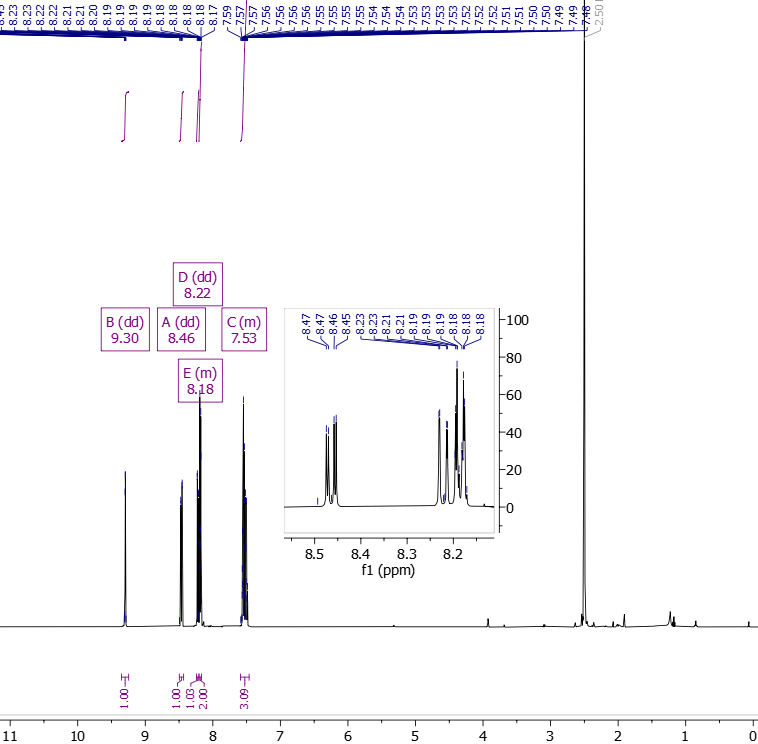
**

**^13^C NMR (126 MHz, DMSO-*d*_6_)**


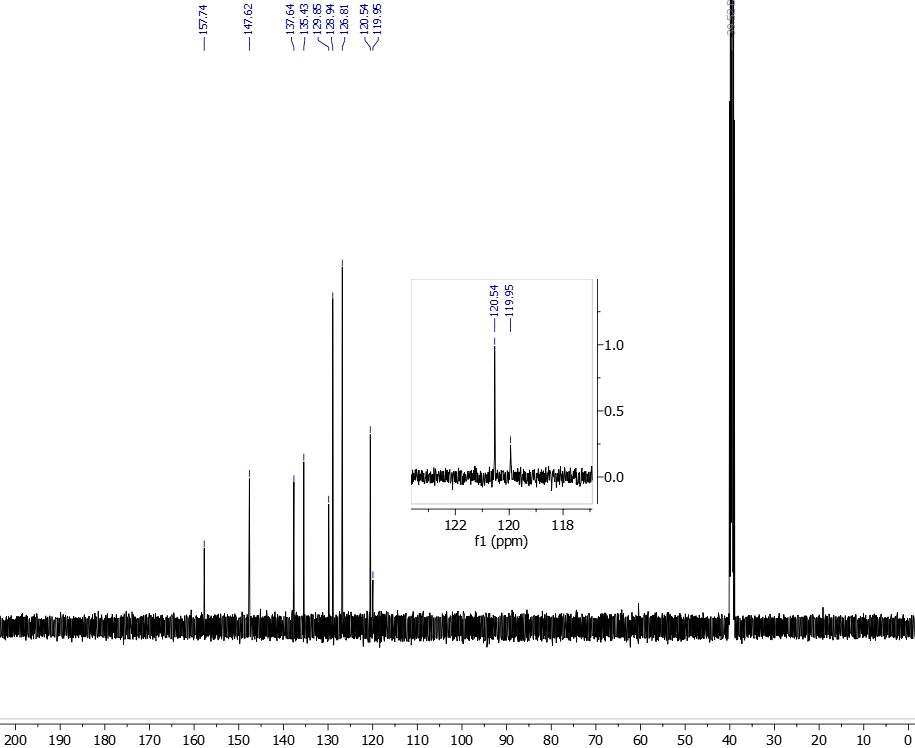


**2-phenyl-5-(1H-tetrazol-5-yl)pyrimidine, 28.**

**^1^H NMR (400 MHz, MeOD)**

**^13^C NMR (101 MHz, MeOD)**

**3-(4-hydroxy-6-methyl-2-phenylpyrimidin-5-yl)propanenitrile,30.**

**^1^H NMR (500 MHz, DMSO-*d_6_*)**

**^13^C NMR (125 MHz, DMSO-*d_6_*)**

**2-(5-methyl-3-oxo-2-phenyl-2,3-dihydro-1H-pyrazol-4-yl)acetonitrile, 31.**

**^1^H NMR (500 MHz, DMSO-*d*_6_)**


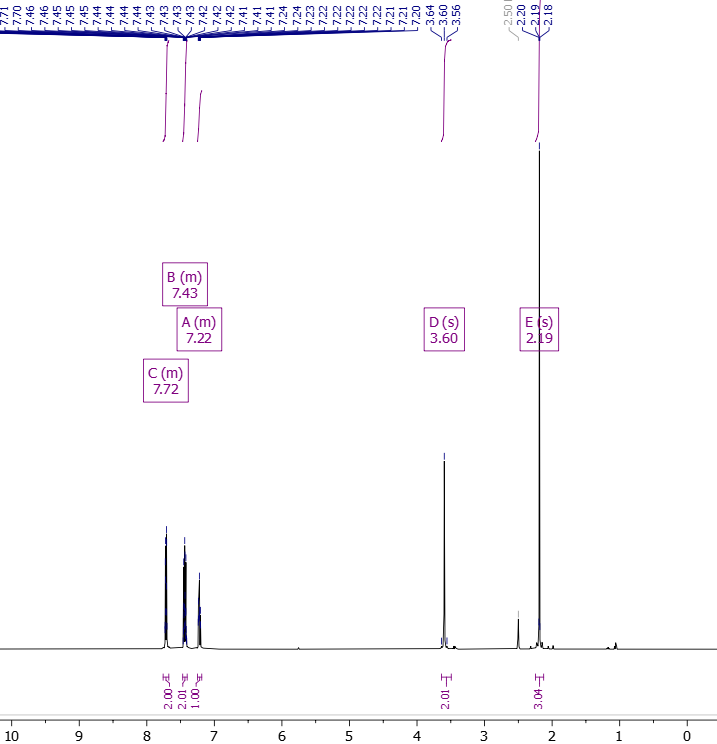


**^13^C NMR (126 MHz, DMSO-*d*_6_)**


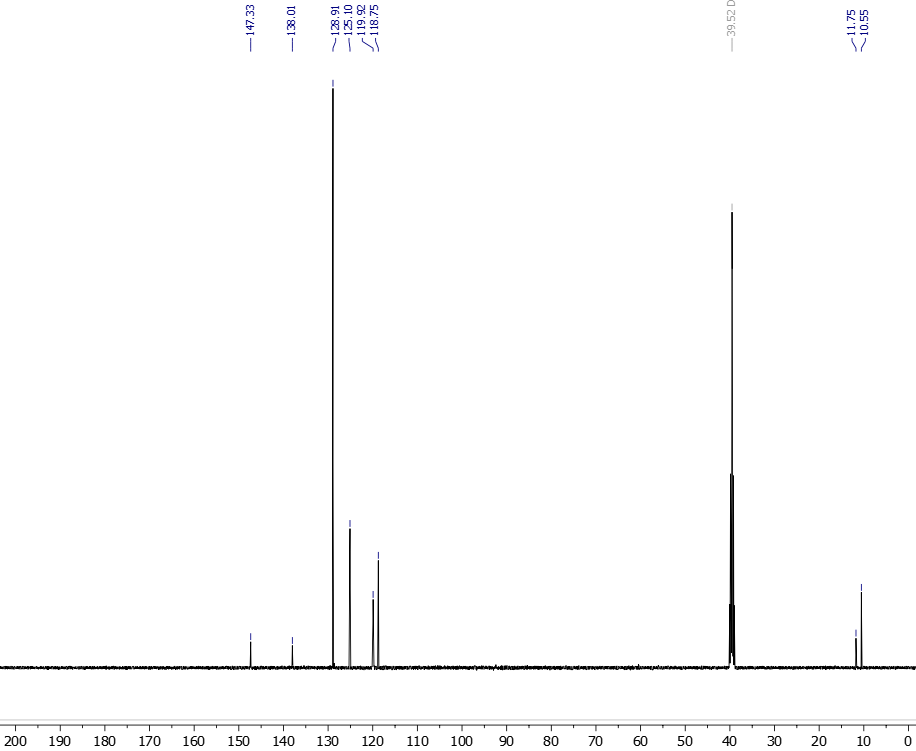


**3-(5-methyl-3-oxo-2-phenyl-2,3-dihydro-1H-pyrazol-4-yl)propanenitrile, 33.**

**^1^H NMR (500 MHz, DMSO-*d*_6_)** *the reported spectrum for characterization.*

**^1^H NMR (400 MHz, CDCl_3_)** *showing tautomerism observed in an alternative solvent.*

**^13^C NMR (101 MHz, DMSO-*d*_6_)** *showing significant signal suppression.*

**^13^C NMR (101 MHz, CDCl_3_)** *the reported spectrum for characterization.*

**^1^H -^13^C NMR HSQC (400 MHz,101 MHz, DMSO-*d*_6_)**

**^1^H -^13^C NMR HSQC (400 MHz,101 MHz, CDCl_3_)**

**^1^H -^13^C NMR HMBC (400 MHz,101 MHz, DMSO-*d*_6_)**

**2-(5-methyl-3-oxo-2-phenyl-2,3-dihydro-1H-pyrazol-4-yl)ethanethioamide, 34.**

**^1^H NMR (500 MHz, DMSO-*d*_6_)**


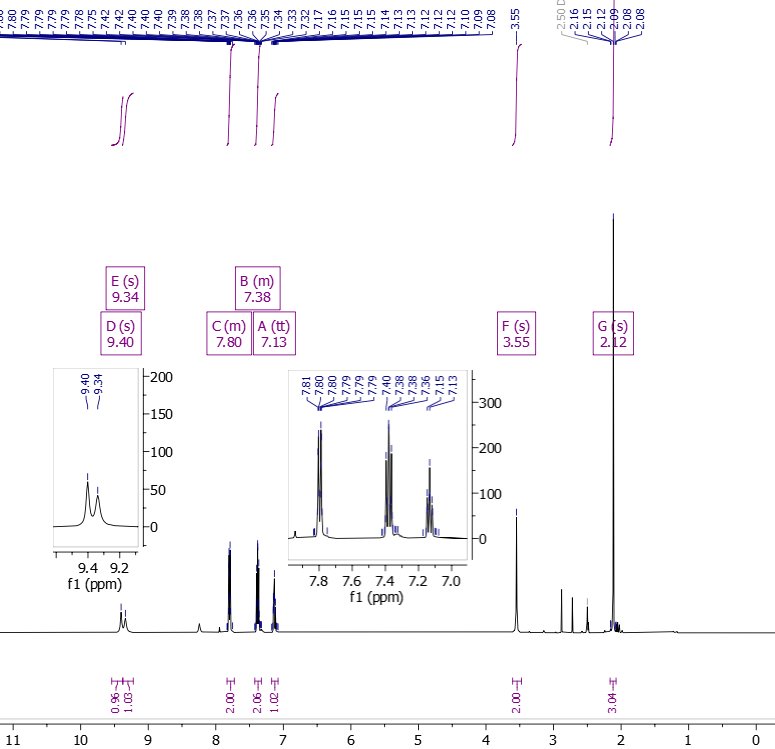


**^13^C NMR (126 MHz, DMSO-*d*_6_)**


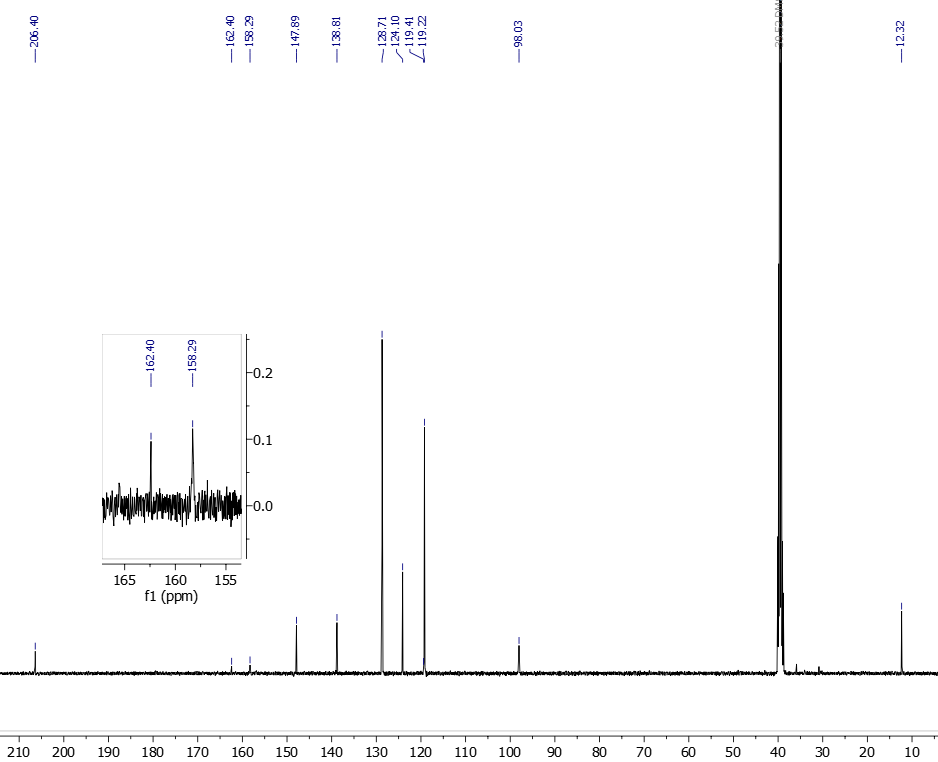


**3-(4-hydroxy-6-methyl-2-phenylpyrimidin-5-yl)propanethioamide, 37.**

**^1^H NMR (500 MHz, DMSO-*d_6_*)**

**^13^C NMR (125 MHz, DMSO-*d_6_*)**

**3-(4-hydroxy-6-methyl-2-phenylpyrimidin-5-yl)propanimidamide, 39.**

**^1^H NMR (500 MHz, DMSO-*d_6_*)**

**^13^C NMR (125 MHz, DMSO-*d_6_*)**

**3-(5-methyl-3-oxo-2-phenyl-2,3-dihydro-1H-pyrazol-4-yl)propanethioamide, 40.**

**^1^H NMR (500 MHz, DMSO-*d*_6_)**

**^13^C NMR (126 MHz, DMSO-*d*_6_)**

**^1^H -^13^C NMR HSQC (400 MHz,101 MHz, DMSO-*d*_6_)**

**^1^H -^13^C NMR HMBC (400 MHz,101 MHz, DMSO-*d*_6_)**

***N*'-hydroxy-3-(5-methyl-3-oxo-2-phenyl-2,3-dihydro-1H-pyrazol-4-yl)propanimidamide, 41.**

**^1^H NMR (500 MHz, DMSO-*d*_6_)**

**^13^C NMR (126 MHz, DMSO-*d*_6_)**

**^1^H -^13^C NMR HSQC (400 MHz,101 MHz, DMSO-*d*_6_)**

**^1^H -^13^C NMR HMBC (400 MHz,101 MHz, DMSO-*d*_6_)**

**3-(5-methyl-3-oxo-2-phenyl-2,3-dihydro-1H-pyrazol-4-yl)propanimidamide, 42.**

**^1^H NMR (500 MHz, DMSO-*d*_6_)**

**^13^C NMR (126 MHz, DMSO-*d*_6_)**

**ethyl 2-((5-methyl-3-oxo-2-phenyl-2,3-dihydro-1H-pyrazol-4-yl)methyl)thiazole-4-carboxylate, 43.**

**^1^H NMR (500 MHz, DMSO-*d*_6_)**


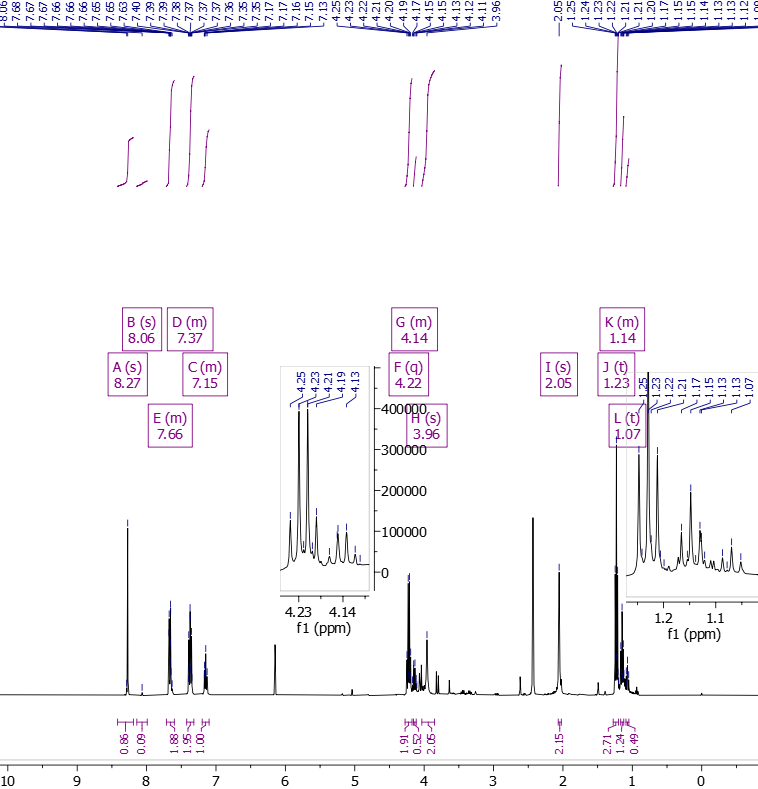


**^13^C NMR (126 MHz, DMSO-*d*_6_)**


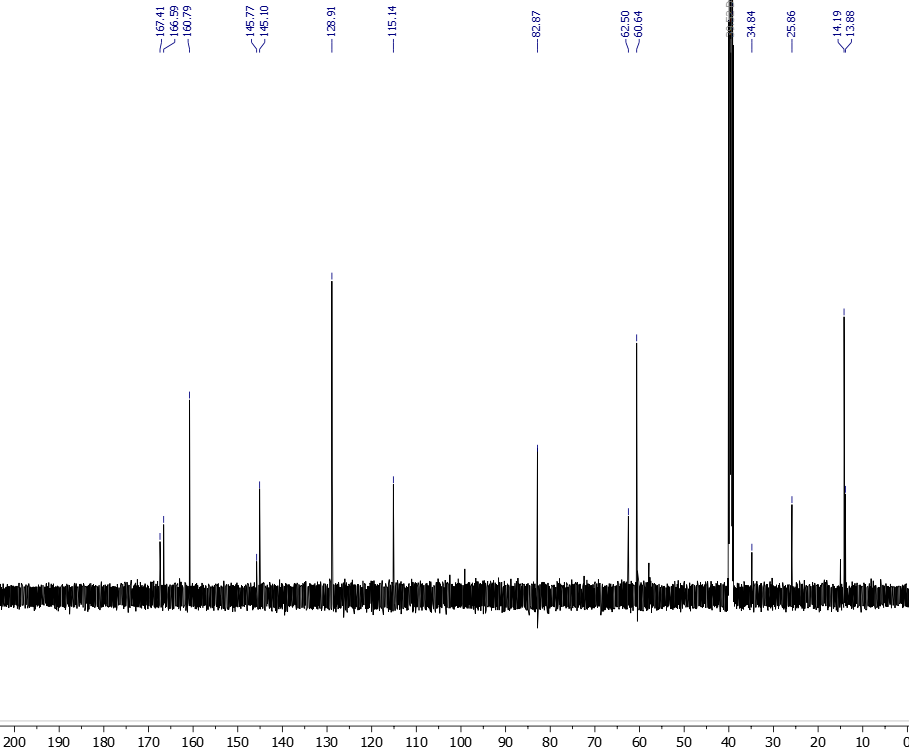


**ethyl 2-(2-(4-hydroxy-6-methyl-2-phenylpyrimidin-5-yl)ethyl)thiazole-4-carboxylate, 44.**

**^1^H NMR (500 MHz, DMSO-*d_6_*)**

**^13^C NMR (125 MHz, DMSO-*d_6_*)**

***tert*-butyl ((3-(2-(4-hydroxy-6-methyl-2-phenylpyrimidin-5-yl)ethyl)-1,2,4-oxadiazol-5-yl)methyl)carbamate, 45.**

**^1^H NMR (500 MHz, DMSO-*d_6_*)**

**^13^C NMR (125 MHz, DMSO-*d_6_*)**

**5-(2-(5-chloropyrimidin-2-yl)ethyl)-6-methyl-2-phenylpyrimidin-4-ol, 46.**

**^1^H NMR (500 MHz, CDCl_3_)**

**^13^C NMR (125 MHz, CDCl_3_)**

**2-(2-(4-hydroxy-6-methyl-2-phenylpyrimidin-5-yl)ethyl)-6-methylpyrimidin-4(3H)-one, 47.**

**^1^H NMR (500 MHz, DMSO-*d_6_*)**

**^13^C NMR (125 MHz, DMSO-*d_6_*)**

**^1^H-^13^C HMBC (400 MHz, DMSO-*d_6_*)**

***tert*-butyl ((3-(2-(5-methyl-3-oxo-2-phenyl-2,3-dihydro-1H-pyrazol-4-yl)ethyl)-1,2,4-oxadiazol-5-yl)methyl)carbamate, 48.**

**^1^H NMR (500 MHz, DMSO-*d*_6_)**

**^13^C NMR (126 MHz, DMSO-*d*_6_)**

**^1^H -^13^C NMR HSQC (400 MHz,101 MHz, DMSO-*d*_6_)**

**^1^H -^13^C NMR HMBC (400 MHz,101 MHz, DMSO-*d*_6_)**

**4-(2-(5-chloropyrimidin-2-yl)ethyl)-5-methyl-2-phenyl-1,2-dihydro-3H-pyrazol-3-one, 49.**

**^1^H NMR (500 MHz, DMSO-*d*_6_)**

**^13^C NMR (126 MHz, DMSO-*d*_6_)**

**^1^H -^13^C NMR HMBC (400 MHz,101 MHz, DMSO-*d*_6_)**

**6-methyl-2-(2-(5-methyl-3-oxo-2-phenyl-2,3-dihydro-1H-pyrazol-4-yl)ethyl)pyrimidin-4(3H)-one, 50.**

**^1^H NMR (500 MHz, DMSO-*d*_6_)**

**^13^C NMR (126 MHz, DMSO-*d*_6_)**

**^1^H -^13^C NMR HSQC (400 MHz,101 MHz, DMSO-*d*_6_)**

**^1^H -^13^C NMR HMBC (400 MHz,101 MHz, DMSO-*d*_6_)**

**ethyl 2-(2-(5-methyl-3-oxo-2-phenyl-2,3-dihydro-1H-pyrazol-4-yl)ethyl)thiazole-4-carboxylate, 51.**

**^1^H NMR (500 MHz, DMSO-*d*_6_)**

**^13^C NMR (126 MHz, DMSO-*d*_6_)**

**^1^H -^13^C NMR HSQC (400 MHz,101 MHz, DMSO-*d*_6_)**

**^1^H -^13^C NMR HMBC (400 MHz,101 MHz, DMSO-*d*_6_)**

**4-((1,4-dihydro-5H-tetrazol-5-ylidene)methyl)-5-methyl-2-phenyl-1,2-dihydro-3H-pyrazol-3-one, 52.**

**^1^H NMR (400 MHz, DMSO-*d*_6_)**


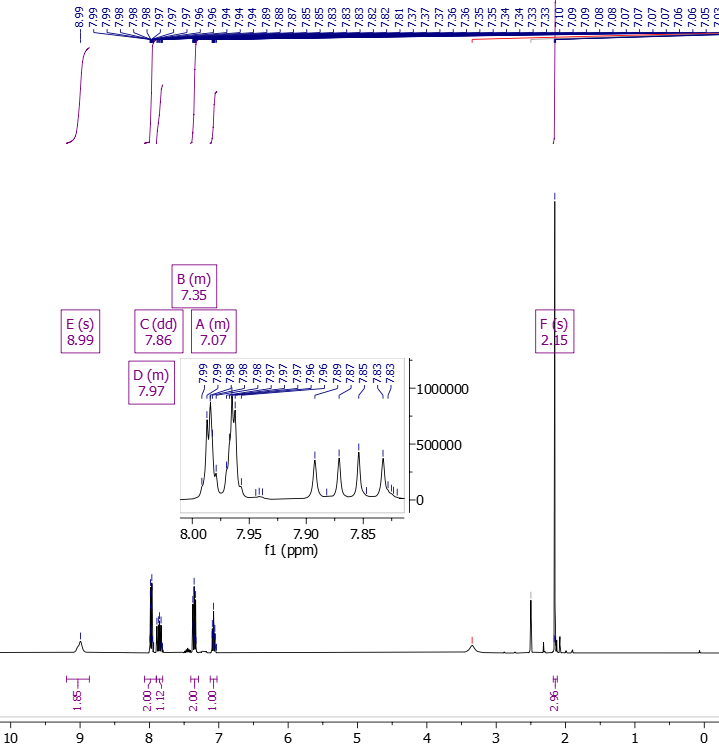


**^1^H NMR (400 MHz, DMSO-*d*_6_+D_2_O spike)**


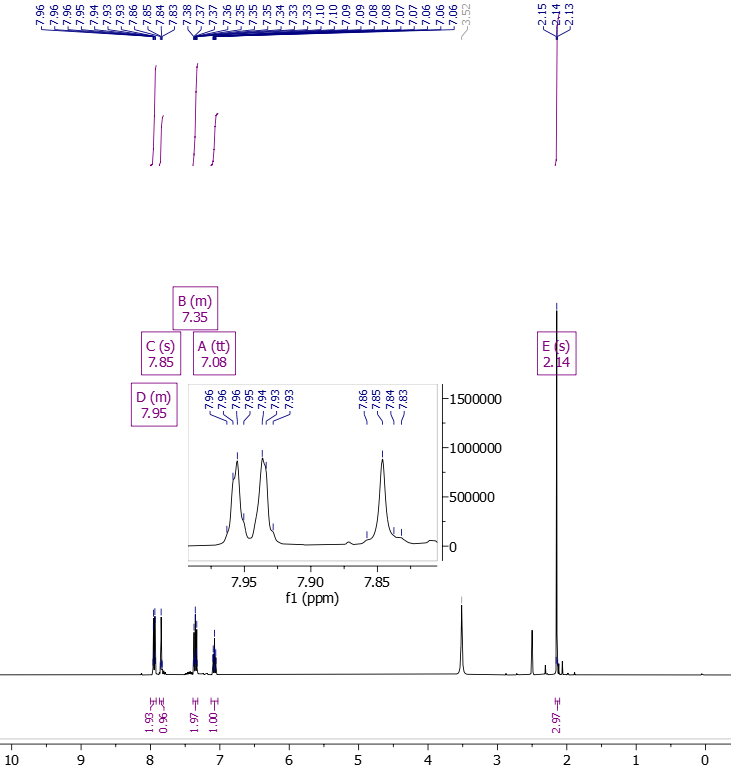


**^13^C NMR (101 MHz, DMSO-*d*_6_)**


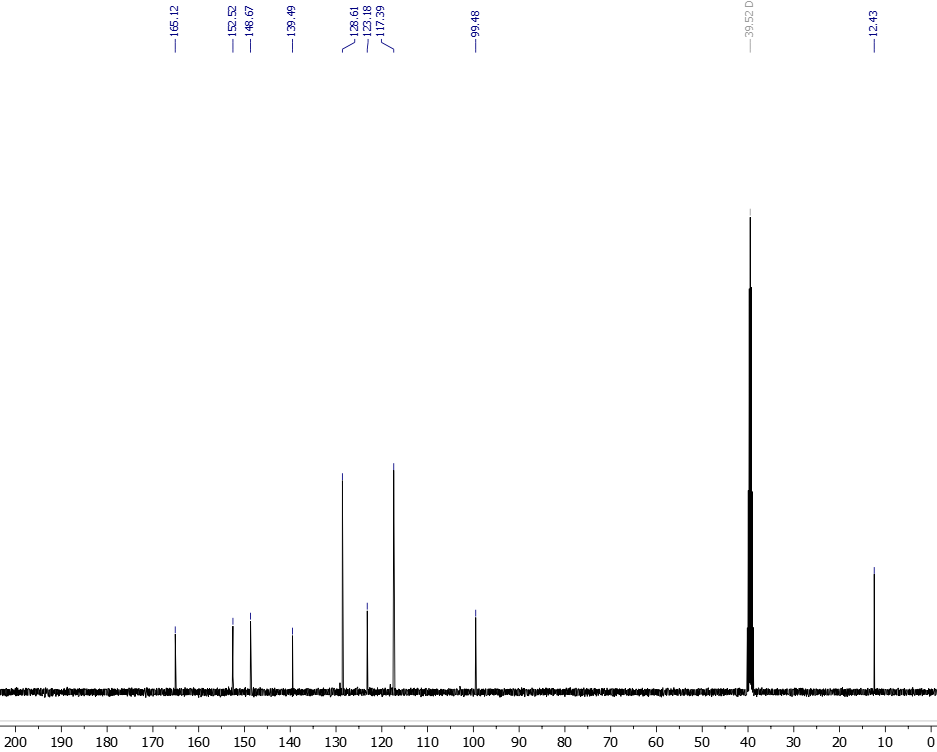


**5-(2-(1H-tetrazol-5-yl)ethyl)-6-methyl-2-phenylpyrimidin-4-ol, 53.**

**^1^H NMR (500 MHz, DMSO-*d_6_*)**

**^13^C NMR (125 MHz, DMSO-*d_6_*)**

**4-(2-(1H-tetrazol-5-yl)ethyl)-5-methyl-2-phenyl-1,2-dihydro-3H-pyrazol-3-one, 54.**

**^1^H NMR (500 MHz, DMSO-*d*_6_)**

**^13^C NMR (126 MHz, DMSO-*d*_6_)**

**2-(1-phenyl-1H-pyrazol-4-yl)pyrimidine-5-carbonitrile, 55.**

**^1^H NMR (500 MHz, CDCl_3_)**


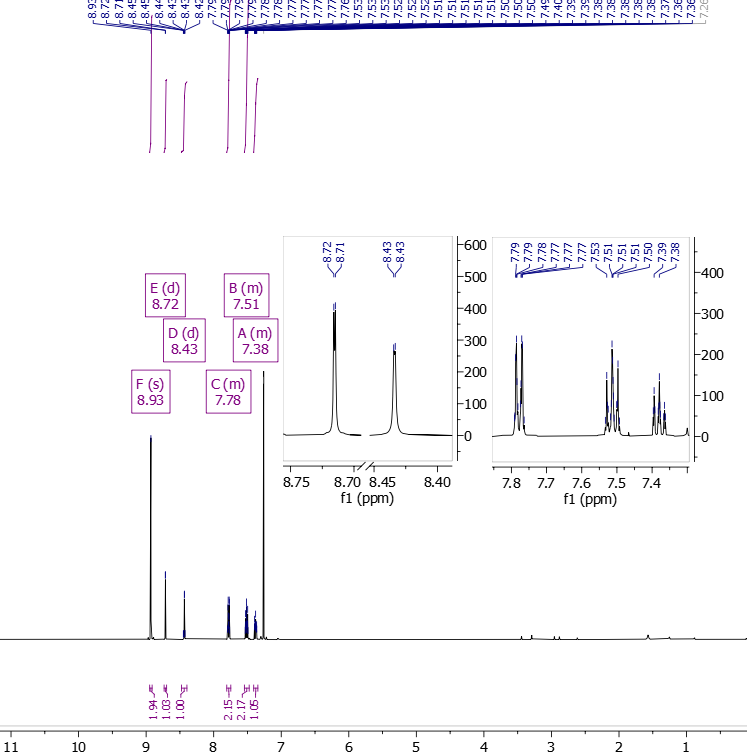


**^13^C NMR (126 MHz, CDCl_3_)**

**
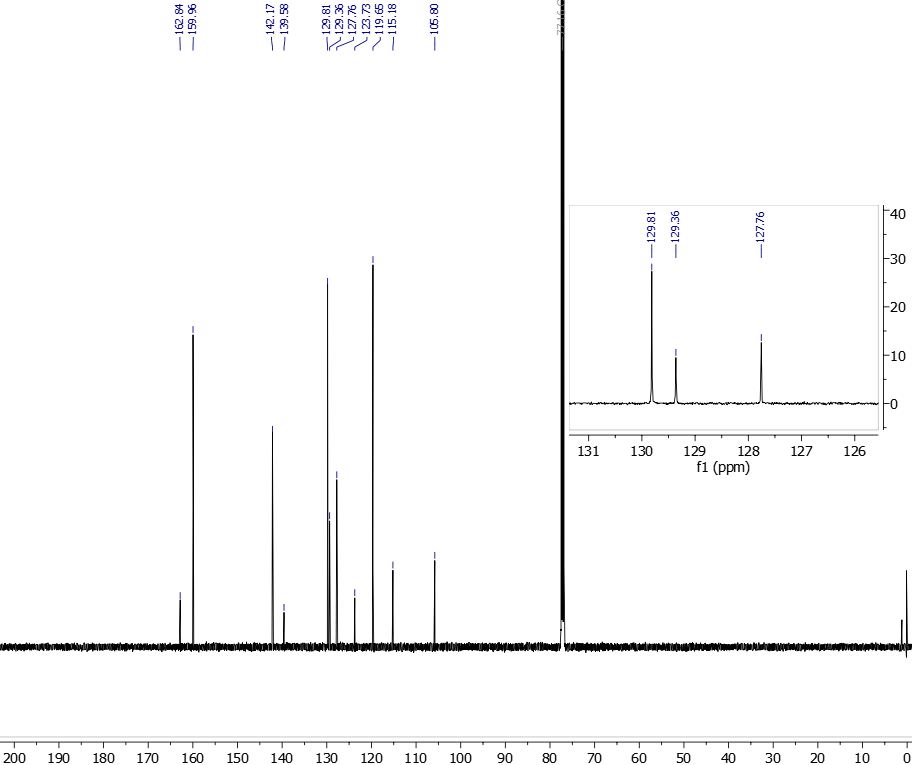
**

**N'-hydroxy-2-(1-phenyl-1H-pyrazol-4-yl)pyrimidine-5-carboximidamide, 56.**

**^1^H NMR (500 MHz, DMSO-*d*_6_)**


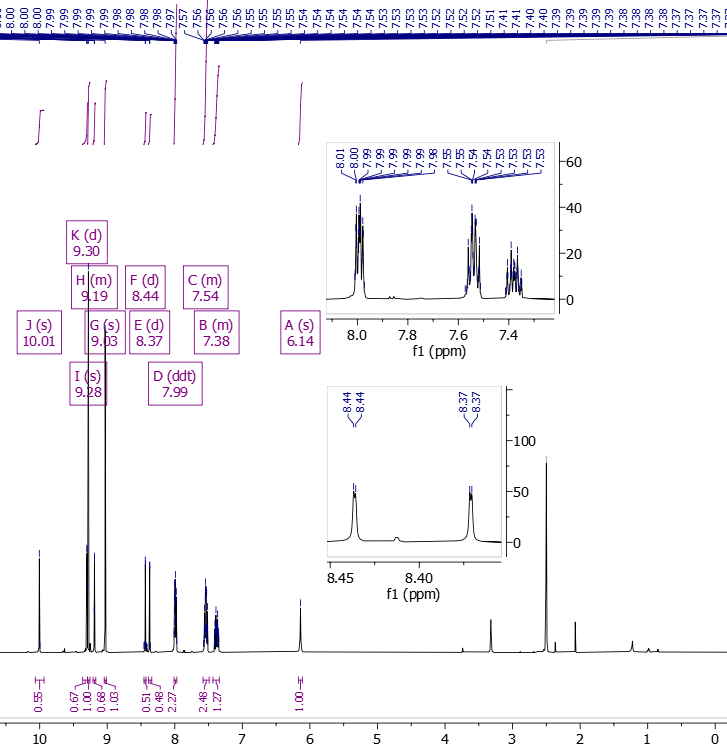


**^13^C NMR (126 MHz, DMSO-*d*_6_)**


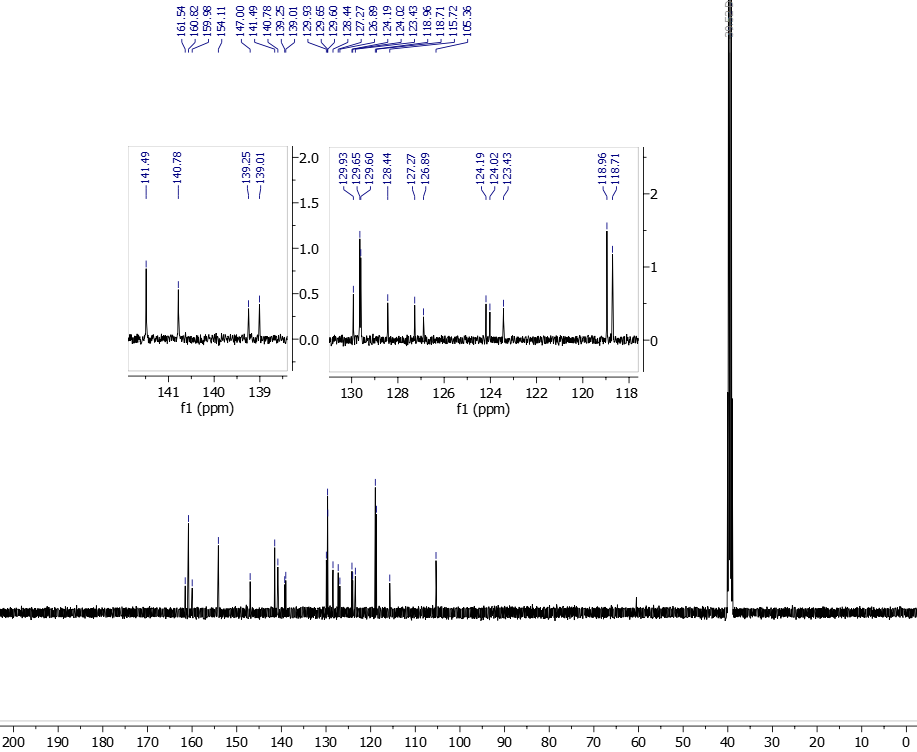


**(3-(2-(1-phenyl-1H-pyrazol-4-yl)pyrimidin-5-yl)-1,2,4-oxadiazol-5-yl)methanamine, 57.**

**^1^H NMR (500 MHz, DMSO-*d*_6_)**


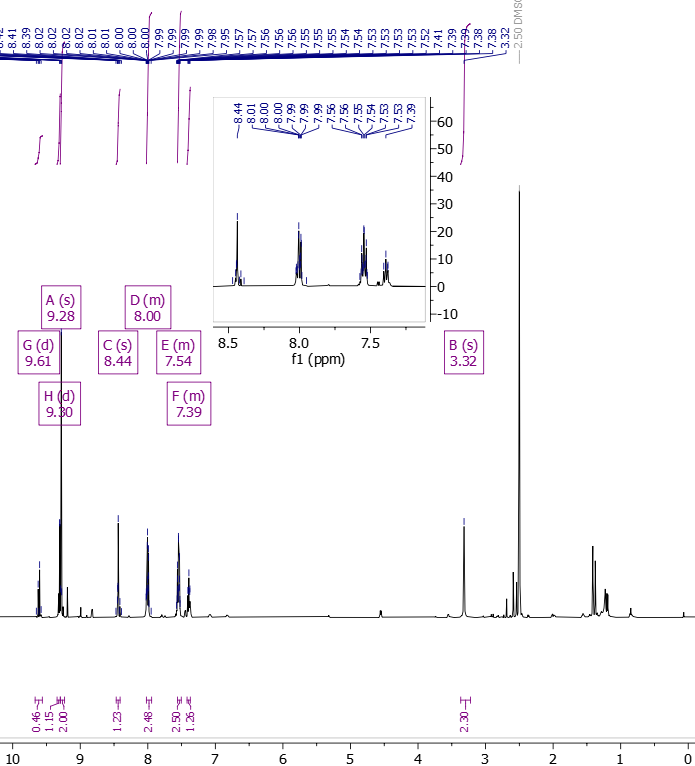


**^13^C NMR (126 MHz, DMSO-*d*_6_**)

**
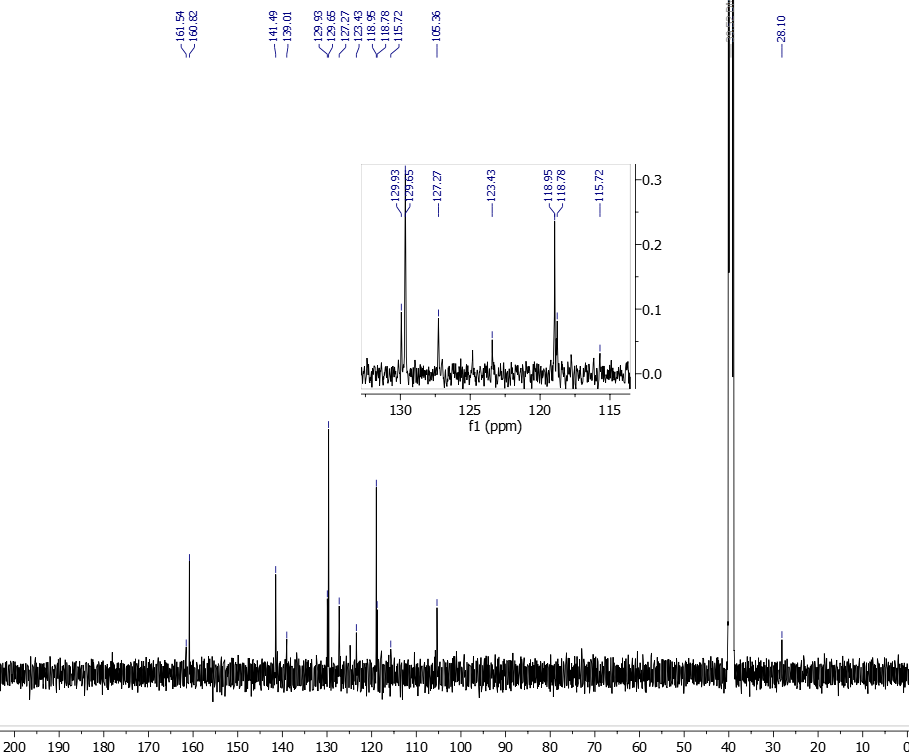
**

**2-(6-phenylpyridin-3-yl)pyrimidine-5-carbonitrile, 58.**

**^1^H NMR (600 MHz, DMF-*d*_7_, 353 K)**

**^13^C NMR (151 MHz, DMF-*d*_7_, 353 K**)

**^1^H -^13^C NMR HMBC (600 MHz,151 MHz, DMF-*d*_7_, 353 K)**

**2-(6-phenylpyridin-3-yl)pyrimidine-5-carbothioamide, 59.**

**^1^H NMR (500 MHz, DMSO-*d*_6_)**

**^13^C NMR (126 MHz, DMSO-*d*_6_**)

**ethyl 2-(2-(6-phenylpyridin-3-yl)pyrimidin-5-yl)thiazole-4-carboxylate, 60.**

**^1^H NMR (500 MHz, THF-*d*_8_)**

**^13^C NMR (126 MHz, THF-*d*_8_)**

**^1^H -^13^C NMR HSQC (400 MHz,101 MHz, THF-*d*_8_)**

**^1^H -^13^C NMR HMBC (400 MHz,101 MHz, THF-*d*_8_)**

**2-(4-methyl-6-oxo-2'-phenyl-1,6-dihydro-[2,5'-bipyrimidin]-5-yl)acetonitrile, 61.**

**^1^H NMR (500 MHz, DMSO-*d6*)**

**^13^C NMR (126 MHz,** **DMSO-*d6*)**

**^1^H -^13^C NMR HSQC (400 MHz,101 MHz, DMSO-*d6*)**

**^1^H -^13^C NMR HMBC (700 MHz, DMSO-*d6*)**

**2-(4-methyl-6-oxo-2'-phenyl-1,6-dihydro-[2,5'-bipyrimidin]-5-yl)acetonitrile, 62.**

**^1^H NMR (500 MHz, DMSO-*d*_6_)**

**^13^C NMR (126 MHz, DMSO-*d6*)**

**^1^H -^13^C NMR HMBC (400 MHz,101 MHz, DMSO-*d*_6_)**

**glycyl-L-phenylalanine benzyl ester hydrochloride, 63.**

**^1^H NMR (500 MHz, DMSO-*d_6_*)**

**^13^C NMR (125 MHz, DMSO-*d_6_*)**

**(*S*)-amino((2-((1-(benzyloxy)-1-oxo-3-phenylpropan-2-yl)amino)-2-oxoethyl)amino)methaniminium formate salt, 64.**

**^1^H NMR (500 MHz, DMSO-*d_6_*)**

**^13^C NMR (125 MHz, DMSO-*d_6_*)**

**benzyl (5-cyanopyrimidin-2-yl)glycyl-L-phenylalaninate, 65.**

**^1^H NMR (500 MHz, DMSO-*d_6_*)**

**^13^C NMR (125 MHz, DMSO-*d_6_*)**

**benzyl (*Z*)-(5-(*N*'-hydroxycarbamimidoyl)pyrimidin-2-yl)glycyl-L-phenylalaninate, 66.**

**^1^H NMR (500 MHz, DMSO-*d_6_*)**

**^13^C NMR (125 MHz, DMSO-*d_6_*)**

**benzyl (5-(5-(((tert-butoxycarbonyl)amino)methyl)-1,2,4-oxadiazol-3-yl)pyrimidin-2-yl)glycyl-L-phenylalaninate, 67.**

**^1^H NMR (500 MHz, CDCl_3_)**

**^13^C NMR (125 MHz, CDCl_3_)**

**(5-(5-(((S)-2-aminopropanamido)methyl)-1,2,4-oxadiazol-3-yl)pyrimidin-2-yl)glycyl-L-phenylalanine- TFA salt, 74.**

**^1^H NMR (500 MHz, DMSO-*d_6_*)**

**^13^C NMR (126 MHz, DMSO-*d_6_*)**:


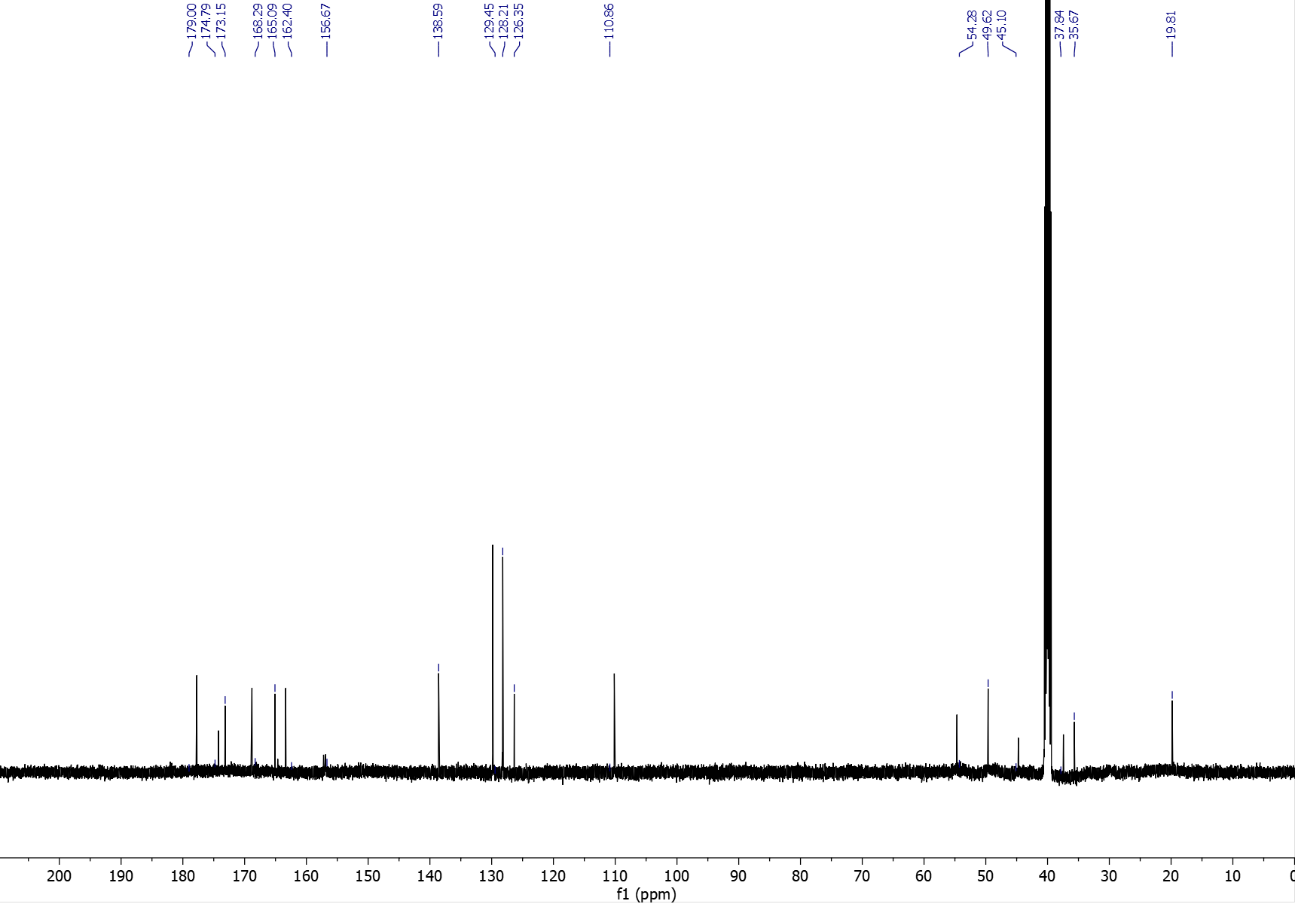


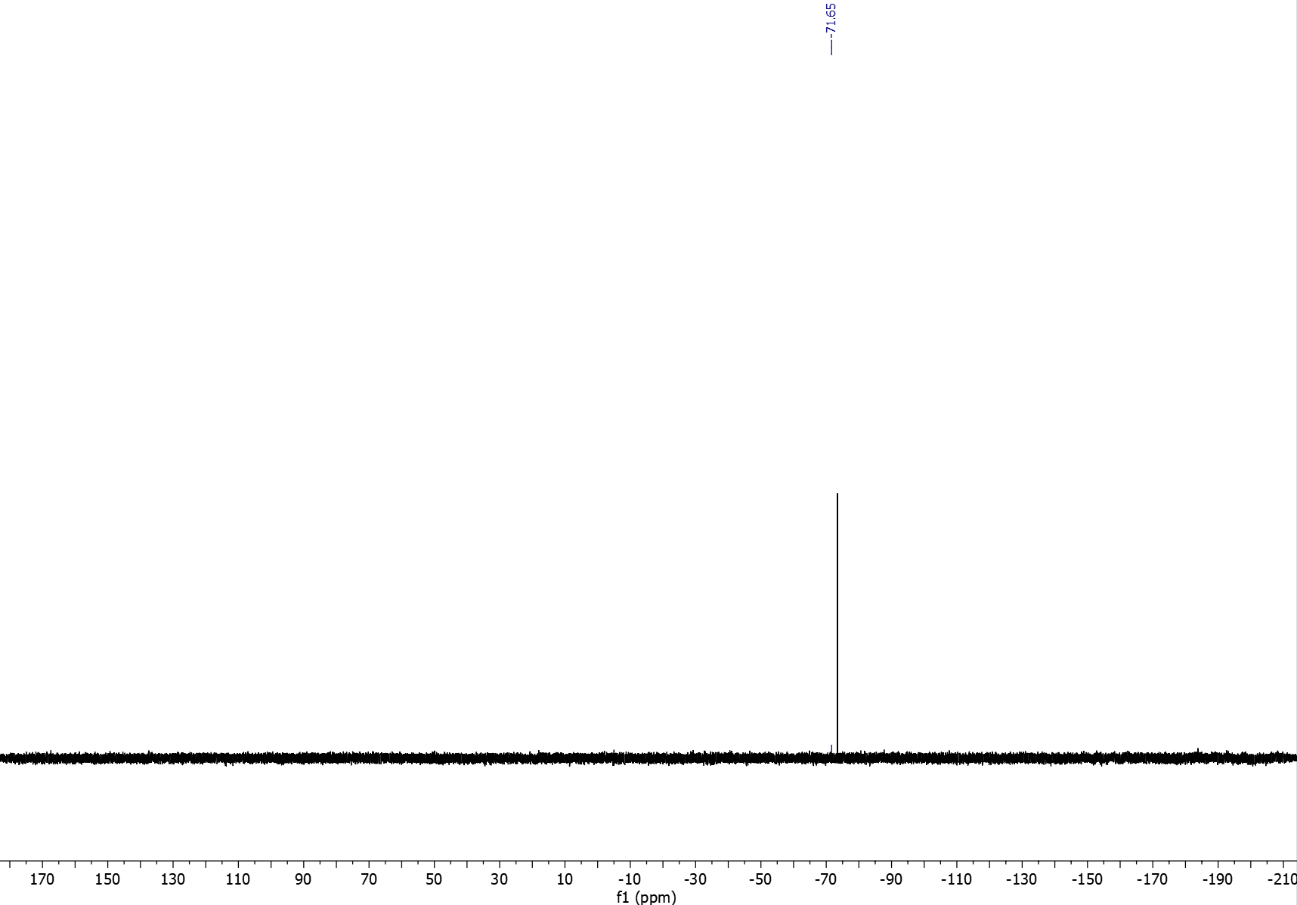
**^19^F NMR (377 MHz, DMSO-*d_6_*)**

# **4. X-ray crystallography**

**(*Z*)-*N*-(2-cyano-3-(dimethylamino)allylidene)-*N*-methylmethanaminium hexafluorophosphate(V), 1-CN.**

Table 1. Crystal data and structure refinement for **1-CN**.

Identification code d2453_a

Empirical formula C8 H14 F6 N3 P

Formula weight 297.19

Temperature 150(2) K

Wavelength 1.54178 Å

Crystal system Monoclinic

Space group Cc

Unit cell dimensions a = 13.1995(11) Å a= 90°.

b = 10.7025(9) Å b= 115.696(3)°.

c = 9.8336(8) Å g = 90°.

Volume 1251.79(18) Å3

Z 4

Density (calculated) 1.577 Mg/m3

Absorption coefficient 2.593 mm-1

F(000) 608

Crystal size 0.330 x 0.170 x 0.060 mm3

Theta range for data collection 5.560 to 66.411°.

Index ranges -15<=h<=15, -12<=k<=12, -11<=l<=11

Reflections collected 12542

Independent reflections 2089 [R(int) = 0.0354]

Completeness to theta = 66.411° 98.6 %

Absorption correction Semi-empirical from equivalents

Max. and min. transmission 0.7528 and 0.5939

Refinement method Full-matrix least-squares on F2

Data / restraints / parameters 2089 / 2 / 167

Goodness-of-fit on F2 1.044

Final R indices [I>2sigma(I)] R1 = 0.0399, wR2 = 0.1028

R indices (all data) R1 = 0.0407, wR2 = 0.1035

Absolute structure parameter 0.172(9)

Extinction coefficient n/a

Largest diff. peak and hole 0.527 and -0.365 e.Å-3

Table 2. Atomic coordinates ( x 104) and equivalent isotropic displacement parameters (Å2x 103)

for d2453_a. U(eq) is defined as one third of the trace of the orthogonalized Uij tensor.

________________________________________________________________________________

x y z U(eq)

________________________________________________________________________________

N(1) 4240(3) 840(3) 6981(4) 25(1)

N(2) 4259(3) 4198(3) 3634(4) 26(1)

N(3) 1942(3) 1781(3) 3033(4) 32(1)

C(1) 4472(3) 1836(4) 6399(4) 23(1)

C(2) 3921(3) 2416(4) 4984(4) 22(1)

C(3) 4489(3) 3478(4) 4790(4) 24(1)

C(4) 2828(4) 2050(4) 3897(5) 24(1)

C(5) 4931(4) 540(5) 8585(5) 33(1)

C(6) 3307(4) -20(4) 6197(5) 33(1)

C(7) 4955(4) 5317(4) 3798(6) 37(1)

C(8) 3379(4) 3988(4) 2107(5) 34(1)

P(1) 6746(1) 2319(1) 2833(1) 30(1)

F(1) 6984(4) 881(3) 3221(6) 76(1)

F(2) 6565(4) 3780(3) 2546(4) 66(1)

F(3) 5461(3) 2085(5) 1863(4) 77(1)

F(4) 6513(3) 2459(3) 4290(4) 51(1)

F(5) 8049(3) 2583(4) 3855(4) 64(1)

F(6) 7000(3) 2193(4) 1402(4) 59(1)

________________________________________________________________________________

Table 3. Bond lengths [Å] and angles [°] for d2453_a.

_____________________________________________________

N(1)-C(1) 1.308(6)

N(1)-C(6) 1.461(6)

N(1)-C(5) 1.475(5)

N(2)-C(3) 1.296(6)

N(2)-C(8) 1.464(6)

N(2)-C(7) 1.475(6)

N(3)-C(4) 1.145(6)

C(1)-C(2) 1.405(6)

C(1)-H(1A) 0.9500

C(2)-C(3) 1.420(6)

C(2)-C(4) 1.429(6)

C(3)-H(3A) 0.9500

C(5)-H(5A) 0.9800

C(5)-H(5B) 0.9800

C(5)-H(5C) 0.9800

C(6)-H(6A) 0.9800

C(6)-H(6B) 0.9800

C(6)-H(6C) 0.9800

C(7)-H(7A) 0.9800

C(7)-H(7B) 0.9800

C(7)-H(7C) 0.9800

C(8)-H(8A) 0.9800

C(8)-H(8B) 0.9800

C(8)-H(8C) 0.9800

P(1)-F(3) 1.564(4)

P(1)-F(1) 1.584(3)

P(1)-F(6) 1.587(3)

P(1)-F(2) 1.589(3)

P(1)-F(5) 1.596(3)

P(1)-F(4) 1.597(3)

C(1)-N(1)-C(6) 125.9(3)

C(1)-N(1)-C(5) 119.4(4)

C(6)-N(1)-C(5) 114.6(4)

C(3)-N(2)-C(8) 126.0(4)

C(3)-N(2)-C(7) 119.3(4)

C(8)-N(2)-C(7) 114.6(4)

N(1)-C(1)-C(2) 132.4(4)

N(1)-C(1)-H(1A) 113.8

C(2)-C(1)-H(1A) 113.8

C(1)-C(2)-C(3) 114.2(3)

C(1)-C(2)-C(4) 122.8(4)

C(3)-C(2)-C(4) 122.7(4)

N(2)-C(3)-C(2) 131.1(4)

N(2)-C(3)-H(3A) 114.4

C(2)-C(3)-H(3A) 114.4

N(3)-C(4)-C(2) 178.4(5)

N(1)-C(5)-H(5A) 109.5

N(1)-C(5)-H(5B) 109.5

H(5A)-C(5)-H(5B) 109.5

N(1)-C(5)-H(5C) 109.5

H(5A)-C(5)-H(5C) 109.5

H(5B)-C(5)-H(5C) 109.5

N(1)-C(6)-H(6A) 109.5

N(1)-C(6)-H(6B) 109.5

H(6A)-C(6)-H(6B) 109.5

N(1)-C(6)-H(6C) 109.5

H(6A)-C(6)-H(6C) 109.5

H(6B)-C(6)-H(6C) 109.5

N(2)-C(7)-H(7A) 109.5

N(2)-C(7)-H(7B) 109.5

H(7A)-C(7)-H(7B) 109.5

N(2)-C(7)-H(7C) 109.5

H(7A)-C(7)-H(7C) 109.5

H(7B)-C(7)-H(7C) 109.5

N(2)-C(8)-H(8A) 109.5

N(2)-C(8)-H(8B) 109.5

H(8A)-C(8)-H(8B) 109.5

N(2)-C(8)-H(8C) 109.5

H(8A)-C(8)-H(8C) 109.5

H(8B)-C(8)-H(8C) 109.5

F(3)-P(1)-F(1) 92.4(3)

F(3)-P(1)-F(6) 92.0(2)

F(1)-P(1)-F(6) 92.0(2)

F(3)-P(1)-F(2) 90.5(3)

F(1)-P(1)-F(2) 176.5(3)

F(6)-P(1)-F(2) 90.0(2)

F(3)-P(1)-F(5) 178.3(3)

F(1)-P(1)-F(5) 88.4(2)

F(6)-P(1)-F(5) 89.4(2)

F(2)-P(1)-F(5) 88.6(2)

F(3)-P(1)-F(4) 89.0(2)

F(1)-P(1)-F(4) 88.3(2)

F(6)-P(1)-F(4) 178.9(2)

F(2)-P(1)-F(4) 89.7(2)

F(5)-P(1)-F(4) 89.6(2)

Symmetry transformations used to generate equivalent atoms: none.

Table 4. Anisotropic displacement parameters (Å2x 103) for d2453_a. The anisotropic

displacement factor exponent takes the form: -2p2[ h2 a*2U11 + ... + 2 h k a* b* U12 ]

______________________________________________________________________________

U11 U22 U33 U23 U13 U12

______________________________________________________________________________

N(1) 23(2) 27(2) 22(2) 1(1) 8(1) 1(1)

N(2) 22(2) 26(2) 28(2) 0(1) 10(2) -2(1)

N(3) 21(2) 34(2) 31(2) 2(2) 4(2) -4(2)

C(1) 17(2) 28(2) 24(2) -5(2) 8(2) -2(2)

C(2) 19(2) 25(2) 20(2) -3(2) 6(2) -2(2)

C(3) 21(2) 26(2) 24(2) -6(2) 9(2) -3(2)

C(4) 22(2) 26(2) 24(2) 0(2) 9(2) 1(2)

C(5) 34(2) 38(2) 23(2) 6(2) 10(2) 8(2)

C(6) 33(2) 31(2) 33(2) 0(2) 13(2) -8(2)

C(7) 36(2) 34(2) 40(3) 1(2) 16(2) -11(2)

C(8) 34(2) 38(3) 21(2) 3(2) 5(2) -5(2)

P(1) 30(1) 33(1) 29(1) -2(1) 14(1) -3(1)

F(1) 108(3) 33(2) 127(4) 8(2) 89(3) 8(2)

F(2) 98(3) 39(2) 76(3) 15(2) 51(2) 12(2)

F(3) 35(2) 138(4) 48(2) -14(2) 10(2) -24(2)

F(4) 52(2) 72(2) 39(2) 11(1) 29(2) 15(2)

F(5) 33(2) 98(3) 55(2) -5(2) 14(2) -10(2)

F(6) 66(2) 84(3) 38(2) -19(2) 34(2) -22(2)

______________________________________________________________________________

Table 5. Hydrogen coordinates ( x 104) and isotropic displacement parameters (Å2x 10 3)

for d2453_a.

________________________________________________________________________________

x y z U(eq)

________________________________________________________________________________

H(1A) 5143 2247 7062 28

H(3A) 5152 3700 5658 29

H(5A) 5599 1076 8985 49

H(5B) 5164 -338 8680 49

H(5C) 4490 681 9157 49

H(6A) 2966 162 5113 49

H(6B) 2742 82 6585 49

H(6C) 3587 -881 6366 49

H(7A) 5489 5420 4859 55

H(7B) 4469 6056 3460 55

H(7C) 5368 5220 3183 55

H(8A) 3256 3088 1925 50

H(8B) 3611 4349 1370 50

H(8C) 2680 4385 2002 50

________________________________________________________________________________

Table 6. Torsion angles [°] for d2453_a.

_______________________________________________________________

C(6)-N(1)-C(1)-C(2) 4.2(7)

C(5)-N(1)-C(1)-C(2) -173.4(4)

N(1)-C(1)-C(2)-C(3) -177.9(4)

N(1)-C(1)-C(2)-C(4) 8.5(7)

C(8)-N(2)-C(3)-C(2) -8.2(7)

C(7)-N(2)-C(3)-C(2) 174.6(4)

C(1)-C(2)-C(3)-N(2) 176.7(4)

C(4)-C(2)-C(3)-N(2) -9.7(7)

________________________________________________________________

Symmetry transformations used to generate equivalent atoms: none.

**
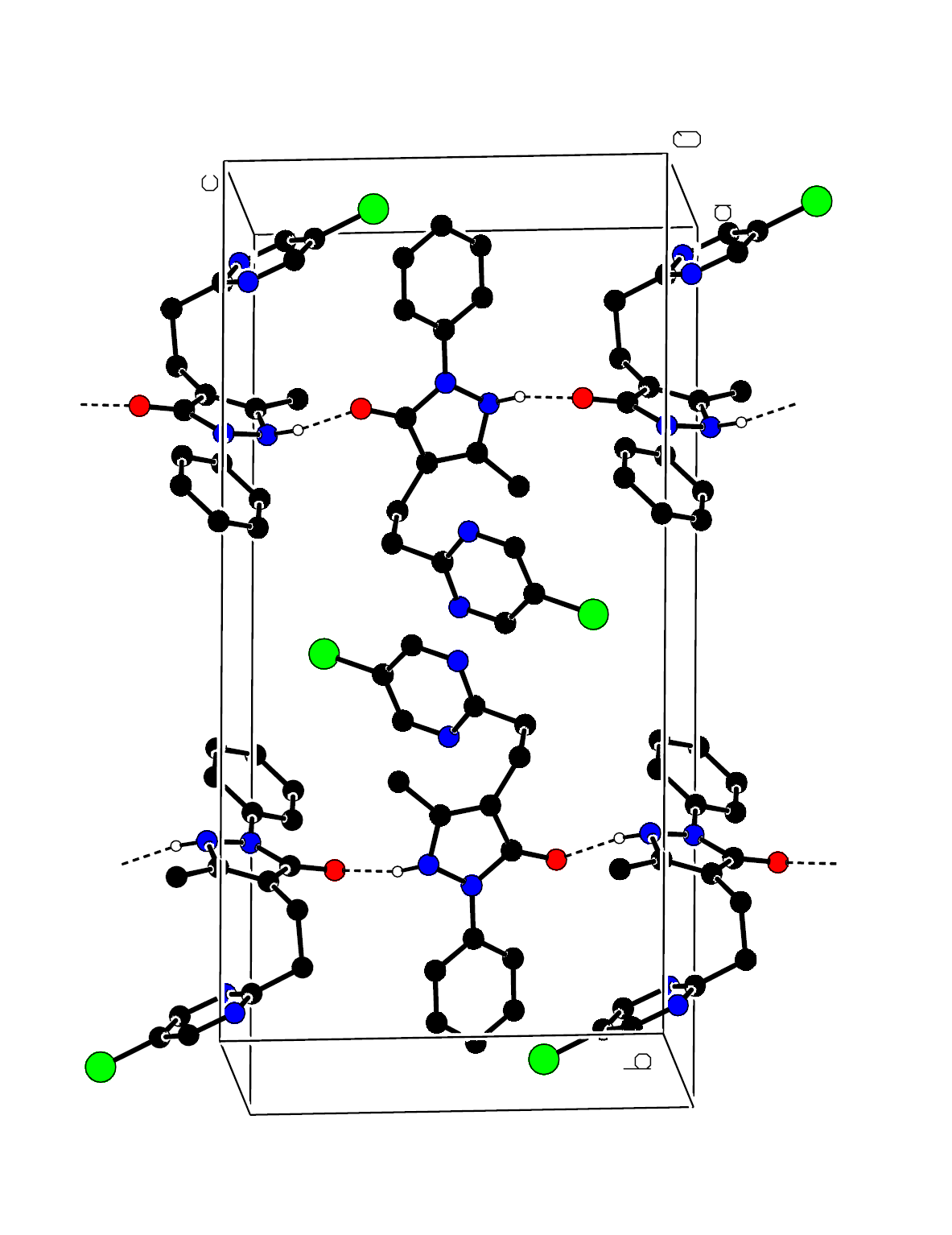

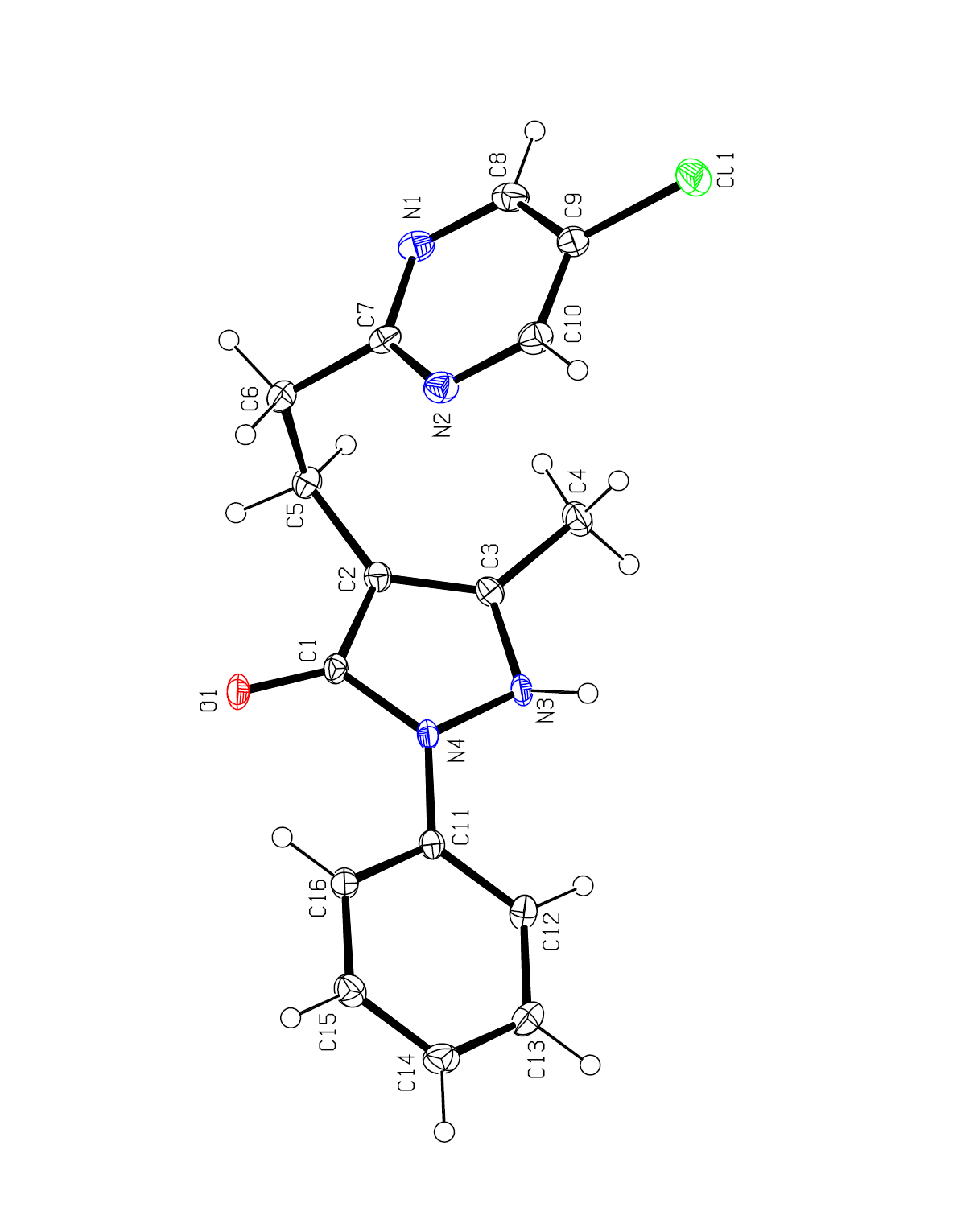
4-(2-(5-chloropyrimidin-2-yl)ethyl)-5-methyl-2-phenyl-1,2-dihydro-3H-pyrazol-3-one, 49.**

Table 7. Crystal data and structure refinement for **49**.

Identification code d2526_a

Empirical formula C16 H15 Cl N4 O

Formula weight 314.77

Temperature 150(2) K

Wavelength 0.71073 Å

Crystal system Monoclinic

Space group P2**_1_**/c

Unit cell dimensions a = 5.1121(8) Å a= 90°.

b = 24.899(4) Å b= 95.703(6)°.

c = 11.622(2) Å g = 90°.

Volume 1472.0(4) Å3

Z 4

Density (calculated) 1.420 Mg/m3

Absorption coefficient 0.267 mm-1

F(000) 656

Crystal size 0.260 x 0.140 x 0.040 mm3

Theta range for data collection 1.636 to 27.486°.

Index ranges -6<=h<=6, -32<=k<=32, -13<=l<=15

Reflections collected 20728

Independent reflections 3356 [R(int) = 0.0436]

Completeness to theta = 25.242° 100.0 %

Absorption correction Semi-empirical from equivalents

Max. and min. transmission 0.7456 and 0.6972

Refinement method Full-matrix least-squares on F2

Data / restraints / parameters 3356 / 0 / 204

Goodness-of-fit on F2 1.029

Final R indices [I>2sigma(I)] R1 = 0.0359, wR2 = 0.0782

R indices (all data) R1 = 0.0541, wR2 = 0.0839

Extinction coefficient n/a

Largest diff. peak and hole 0.222 and -0.289 e.Å-3

Table 8. Atomic coordinates ( x 104) and equivalent isotropic displacement parameters (Å2x 103)

for d2526_a. U(eq) is defined as one third of the trace of the orthogonalized Uij tensor.

________________________________________________________________________________

x y z U(eq)

________________________________________________________________________________

Cl(1) 5275(1) 5175(1) 8053(1) 38(1)

O(1) 6421(2) 7462(1) 2873(1) 26(1)

N(1) 2427(2) 5523(1) 4839(1) 27(1)

N(2) 6260(2) 6058(1) 5300(1) 27(1)

N(3) 4503(2) 7656(1) 5622(1) 20(1)

N(4) 6120(2) 7765(1) 4755(1) 19(1)

C(1) 5408(3) 7434(1) 3811(1) 19(1)

C(2) 3365(3) 7091(1) 4155(1) 21(1)

C(3) 2974(3) 7227(1) 5257(1) 21(1)

C(4) 1250(3) 6982(1) 6080(1) 28(1)

C(5) 2093(3) 6654(1) 3409(1) 25(1)

C(6) 3821(3) 6144(1) 3410(1) 26(1)

C(7) 4207(3) 5889(1) 4579(1) 23(1)

C(8) 2745(3) 5308(1) 5897(1) 28(1)

C(9) 4806(3) 5461(1) 6690(1) 25(1)

C(10) 6533(3) 5843(1) 6357(1) 27(1)

C(11) 7683(3) 8238(1) 4821(1) 19(1)

C(12) 7346(3) 8624(1) 5659(1) 25(1)

C(13) 8915(3) 9080(1) 5728(1) 30(1)

C(14) 10800(3) 9157(1) 4973(1) 29(1)

C(15) 11133(3) 8770(1) 4141(1) 27(1)

C(16) 9598(3) 8308(1) 4059(1) 22(1)

Table 9. Bond lengths [Å] and angles [°] for **49**.

_____________________________________________________________________________________________

Cl(1)-C(9) 1.7311(16)

O(1)-C(1) 1.2535(17)

N(1)-C(8) 1.336(2)

N(1)-C(7) 1.3434(19)

N(2)-C(10) 1.334(2)

N(2)-C(7) 1.3445(19)

N(3)-C(3) 1.3672(18)

N(3)-N(4) 1.3917(16)

N(3)-H(3N) 0.91(2)

N(4)-C(1) 1.3919(18)

N(4)-C(11) 1.4193(18)

C(1)-C(2) 1.437(2)

C(2)-C(3) 1.3583(19)

C(2)-C(5) 1.4999(19)

C(3)-C(4) 1.4940(19)

C(4)-H(4A) 0.9800

C(4)-H(4B) 0.9800

C(4)-H(4C) 0.9800

C(5)-C(6) 1.546(2)

C(5)-H(5A) 0.9900

C(5)-H(5B) 0.9900

C(6)-C(7) 1.495(2)

C(6)-H(6A) 0.9900

C(6)-H(6B) 0.9900

C(8)-C(9) 1.383(2)

C(8)-H(8A) 0.9500

C(9)-C(10) 1.380(2)

C(10)-H(10A) 0.9500

C(11)-C(12) 1.391(2)

C(11)-C(16) 1.396(2)

C(12)-C(13) 1.389(2)

C(12)-H(12A) 0.9500

C(13)-C(14) 1.379(2)

C(13)-H(13A) 0.9500

C(14)-C(15) 1.388(2)

C(14)-H(14A) 0.9500

C(15)-C(16) 1.389(2)

C(15)-H(15A) 0.9500

C(16)-H(16A) 0.9500

C(8)-N(1)-C(7) 117.10(13)

C(10)-N(2)-C(7) 116.84(13)

C(3)-N(3)-N(4) 106.87(11)

C(3)-N(3)-H(3N) 119.0(11)

N(4)-N(3)-H(3N) 118.3(12)

N(3)-N(4)-C(1) 109.16(11)

N(3)-N(4)-C(11) 119.60(11)

C(1)-N(4)-C(11) 129.38(11)

O(1)-C(1)-N(4) 123.72(13)

O(1)-C(1)-C(2) 130.46(13)

N(4)-C(1)-C(2) 105.81(11)

C(3)-C(2)-C(1) 107.09(12)

C(3)-C(2)-C(5) 129.06(13)

C(1)-C(2)-C(5) 123.79(12)

C(2)-C(3)-N(3) 110.74(12)

C(2)-C(3)-C(4) 130.80(13)

N(3)-C(3)-C(4) 118.45(12)

C(3)-C(4)-H(4A) 109.5

C(3)-C(4)-H(4B) 109.5

H(4A)-C(4)-H(4B) 109.5

C(3)-C(4)-H(4C) 109.5

H(4A)-C(4)-H(4C) 109.5

H(4B)-C(4)-H(4C) 109.5

C(2)-C(5)-C(6) 112.41(12)

C(2)-C(5)-H(5A) 109.1

C(6)-C(5)-H(5A) 109.1

C(2)-C(5)-H(5B) 109.1

C(6)-C(5)-H(5B) 109.1

H(5A)-C(5)-H(5B) 107.9

C(7)-C(6)-C(5) 111.87(12)

C(7)-C(6)-H(6A) 109.2

C(5)-C(6)-H(6A) 109.2

C(7)-C(6)-H(6B) 109.2

C(5)-C(6)-H(6B) 109.2

H(6A)-C(6)-H(6B) 107.9

N(1)-C(7)-N(2) 125.24(14)

N(1)-C(7)-C(6) 117.48(13)

N(2)-C(7)-C(6) 117.24(13)

N(1)-C(8)-C(9) 121.23(14)

N(1)-C(8)-H(8A) 119.4

C(9)-C(8)-H(8A) 119.4

C(10)-C(9)-C(8) 117.95(14)

C(10)-C(9)-Cl(1) 120.44(12)

C(8)-C(9)-Cl(1) 121.60(12)

N(2)-C(10)-C(9) 121.63(14)

N(2)-C(10)-H(10A) 119.2

C(9)-C(10)-H(10A) 119.2

C(12)-C(11)-C(16) 120.03(13)

C(12)-C(11)-N(4) 120.14(13)

C(16)-C(11)-N(4) 119.82(12)

C(13)-C(12)-C(11) 119.70(14)

C(13)-C(12)-H(12A) 120.2

C(11)-C(12)-H(12A) 120.1

C(14)-C(13)-C(12) 120.83(14)

C(14)-C(13)-H(13A) 119.6

C(12)-C(13)-H(13A) 119.6

C(13)-C(14)-C(15) 119.28(14)

C(13)-C(14)-H(14A) 120.4

C(15)-C(14)-H(14A) 120.4

C(14)-C(15)-C(16) 120.95(14)

C(14)-C(15)-H(15A) 119.5

C(16)-C(15)-H(15A) 119.5

C(15)-C(16)-C(11) 119.21(1)

Table 10: Torsion angles [°] for **49**.

________________________________________________________________________________________________________

C(3)-N(3)-N(4)-C(1) 5.58(15)

C(3)-N(3)-N(4)-C(11) 171.50(11)

N(3)-N(4)-C(1)-O(1) 175.88(13)

C(11)-N(4)-C(1)-O(1) 11.8(2)

N(3)-N(4)-C(1)-C(2) -3.26(14)

C(11)-N(4)-C(1)-C(2) -167.37(13)

O(1)-C(1)-C(2)-C(3) -179.36(15)

N(4)-C(1)-C(2)-C(3) -0.30(15)

O(1)-C(1)-C(2)-C(5) 3.5(2)

N(4)-C(1)-C(2)-C(5) -177.47(12)

C(1)-C(2)-C(3)-N(3) 3.85(16)

C(5)-C(2)-C(3)-N(3) -179.18(13)

C(1)-C(2)-C(3)-C(4) -175.00(15)

C(5)-C(2)-C(3)-C(4) 2.0(3)

N(4)-N(3)-C(3)-C(2) -5.86(16)

N(4)-N(3)-C(3)-C(4) 173.15(12)

C(3)-C(2)-C(5)-C(6) -98.13(18)

C(1)-C(2)-C(5)-C(6) 78.38(17)

C(2)-C(5)-C(6)-C(7) 64.47(16)

C(8)-N(1)-C(7)-N(2) -0.6(2)

C(8)-N(1)-C(7)-C(6) -178.20(13)

C(10)-N(2)-C(7)-N(1) 0.0(2)

C(10)-N(2)-C(7)-C(6) 177.57(13)

C(5)-C(6)-C(7)-N(1) 88.97(15)

C(5)-C(6)-C(7)-N(2) -88.79(16)

C(7)-N(1)-C(8)-C(9) 0.6(2)

N(1)-C(8)-C(9)-C(10) 0.0(2)

N(1)-C(8)-C(9)-Cl(1) -178.66(11)

C(7)-N(2)-C(10)-C(9) 0.7(2)

C(8)-C(9)-C(10)-N(2) -0.7(2)

Cl(1)-C(9)-C(10)-N(2) 178.01(12)

N(3)-N(4)-C(11)-C(12) -8.50(19)

C(1)-N(4)-C(11)-C(12) 154.19(14)

N(3)-N(4)-C(11)-C(16) 170.16(12)

C(1)-N(4)-C(11)-C(16) -27.1(2)

C(16)-C(11)-C(12)-C(13) 0.3(2)

N(4)-C(11)-C(12)-C(13) 178.93(13)

C(11)-C(12)-C(13)-C(14) 0.3(2)

C(12)-C(13)-C(14)-C(15) -0.4(2)

C(13)-C(14)-C(15)-C(16) 0.0(2)

C(14)-C(15)-C(16)-C(11) 0.5(2)

C(12)-C(11)-C(16)-C(15) -0.7(2)

N(4)-C(11)-C(16)-C(15) -179.32(13)

Table 11. Hydrogen bonds for **49** [Å and °].

____________________________________________________________________________

D-H...A d(D-H) d(H...A) d(D...A) <(DHA)

____________________________________________________________________________

N(3)-H(3N)...O(1)#1 0.91(2) 1.82(2) 2.7172(16) 167.4(17)

____________________________________________________________________________

**
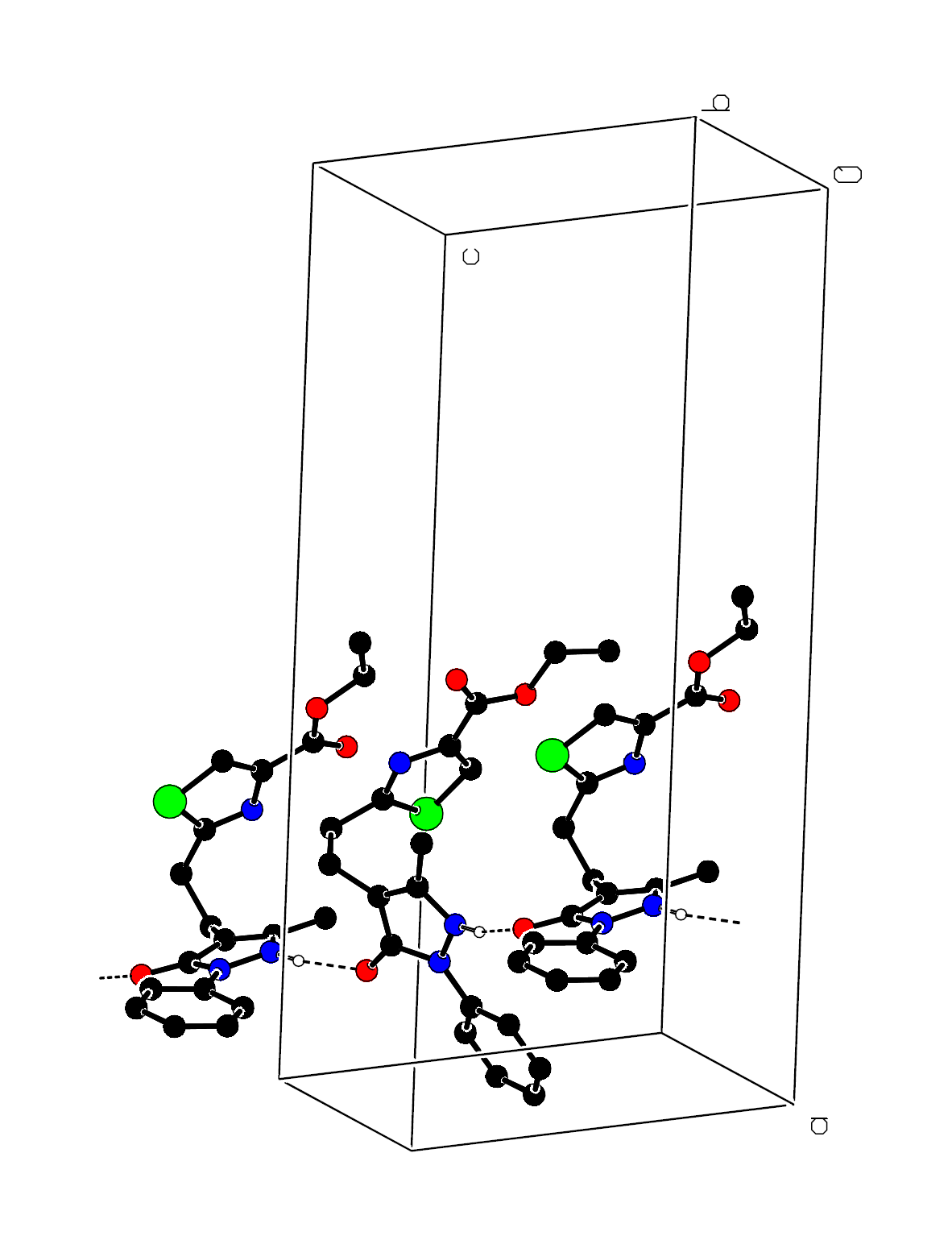
**
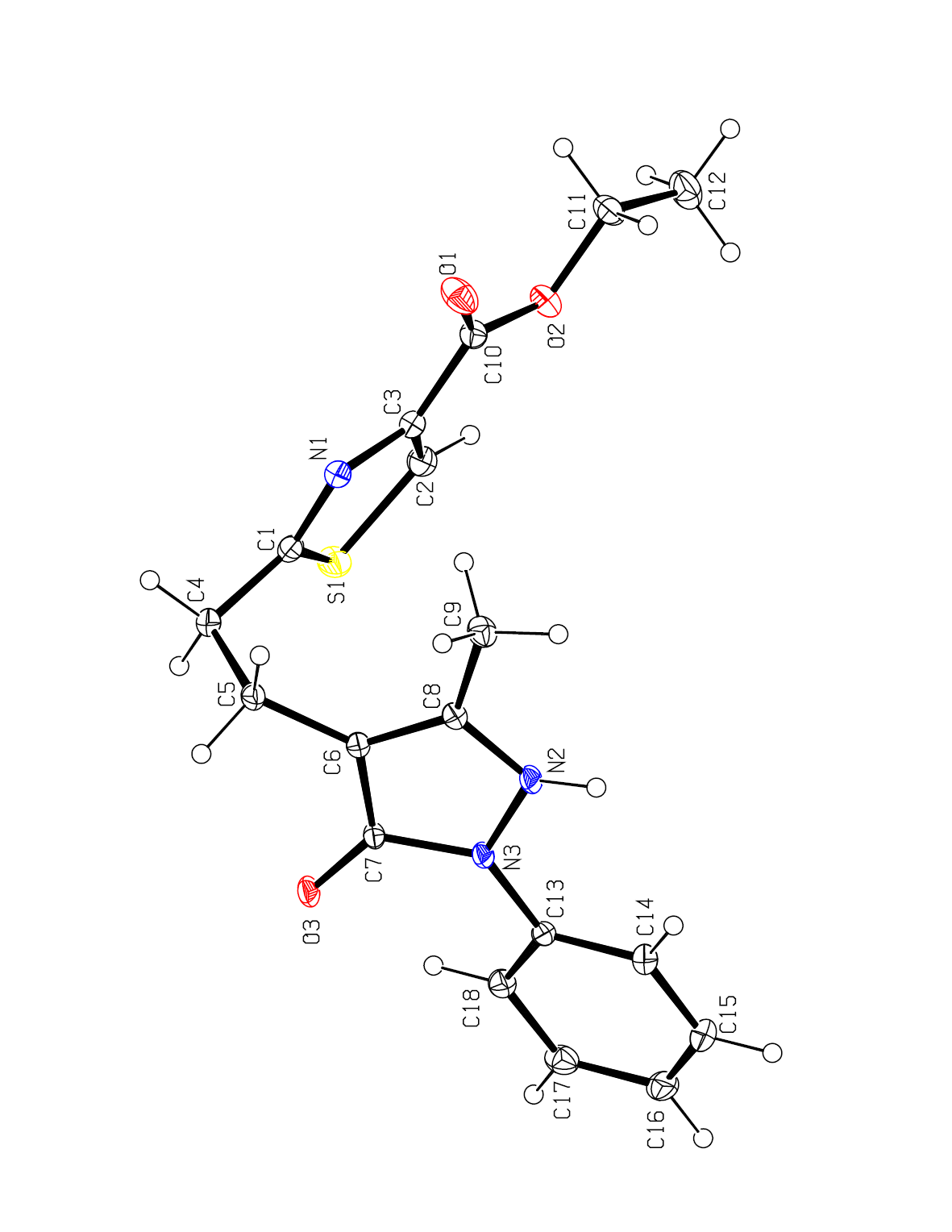
 **ethyl 2-(2-(5-methyl-3-oxo-2-phenyl-2,3-dihydro-1H-pyrazol-4-yl)ethyl)thiazole-4-carboxylate, 51.**

Table 12. Crystal data and structure refinement for **51.**

Identification code d2524a_a

Empirical formula C18 H19 N3 O3 S

Formula weight 357.42

Temperature 150(2) K

Wavelength 1.54178 Å

Crystal system Monoclinic

Space group P2**_1_**/c

Unit cell dimensions a = 24.6479(14) Å a= 90°.

b = 5.8239(3) Å b= 94.731(2)°.

c = 11.8217(6) Å g = 90°.

Volume 1691.19(16) Å3

Z 4

Density (calculated) 1.404 Mg/m3

Absorption coefficient 1.900 mm-1

F(000) 752

Crystal size 0.370 x 0.090 x 0.020 mm3

Theta range for data collection 1.798 to 66.035°.

Index ranges -29<=h<=28, -6<=k<=6, -13<=l<=13

Reflections collected 31352

Independent reflections 2878 [R(int) = 0.0307]

Completeness to theta = 66.035° 98.0 %

Absorption correction Semi-empirical from equivalents

Max. and min. transmission 0.7527 and 0.6557

Refinement method Full-matrix least-squares on F2

Data / restraints / parameters 2878 / 0 / 232

Goodness-of-fit (GOOF) on F2 1.088

Final R indices [I>2sigma(I)] R1 = 0.0301, wR2 = 0.0826

R indices (all data) R1 = 0.0325, wR2 = 0.0838

Extinction coefficient n/a

Largest diff. peak and hole 0.211 and -0.302 e.Å-3

Table 13. Atomic coordinates ( x 104) and equivalent isotropic displacement parameters (Å2x 103)

for **51**. U(eq) is defined as one third of the trace of the orthogonalized Uij tensor.

________________________________________________________________________________

x y z U(eq)

________________________________________________________________________________

S(1) 6808(1) 5035(1) 8150(1) 29(1)

O(1) 5910(1) 10565(2) 5525(1) 36(1)

O(2) 5806(1) 6854(2) 5023(1) 28(1)

O(3) 8562(1) 6213(2) 9145(1) 24(1)

N(1) 6610(1) 9132(2) 7439(1) 22(1)

N(2) 8327(1) 7761(2) 6319(1) 20(1)

N(3) 8582(1) 6418(2) 7169(1) 18(1)

C(1) 6873(1) 7998(3) 8258(1) 22(1)

C(2) 6404(1) 5344(3) 6920(1) 26(1)

C(3) 6343(1) 7621(2) 6681(1) 21(1)

C(4) 7228(1) 9059(3) 9208(1) 25(1)

C(5) 7735(1) 10244(2) 8796(1) 21(1)

C(6) 8024(1) 8911(2) 7946(1) 18(1)

C(7) 8400(1) 7099(2) 8207(1) 18(1)

C(8) 8006(1) 9287(2) 6800(1) 19(1)

C(9) 7710(1) 11082(2) 6095(1) 25(1)

C(10) 6002(1) 8563(3) 5693(1) 23(1)

C(11) 5446(1) 7504(3) 4043(1) 31(1)

C(12) 5292(1) 5327(3) 3411(1) 38(1)

C(13) 8948(1) 4643(2) 6919(1) 19(1)

C(14) 9199(1) 4684(3) 5903(1) 26(1)

C(15) 9549(1) 2912(3) 5671(1) 33(1)

C(16) 9656(1) 1137(3) 6427(1) 32(1)

C(17) 9405(1) 1112(3) 7435(1) 28(1)

C(18) 9049(1) 2850(2) 7687(1) 23(1)

________________________________________________________________________________

Table 14. Bond lengths [Å] and angles [°] for **51**.

__________________________________________________________________________________________________

S(1)-C(2) 1.7027(15)

S(1)-C(1) 1.7364(15)

O(1)-C(10) 1.2017(18)

O(2)-C(10) 1.3374(17)

O(2)-C(11) 1.4507(17)

O(3)-C(7) 1.2580(16)

N(1)-C(1) 1.3006(18)

N(1)-C(3) 1.3830(18)

N(2)-C(8) 1.3470(18)

N(2)-N(3) 1.3834(15)

N(2)-H(2N) 0.898(18)

N(3)-C(7) 1.3986(16)

N(3)-C(13) 1.4182(17)

C(1)-C(4) 1.4995(19)

C(2)-C(3) 1.361(2)

C(2)-H(2) 0.9500

C(3)-C(10) 1.4861(19)

C(4)-C(5) 1.541(2)

C(4)-H(4A) 0.9900

C(4)-H(4B) 0.9900

C(5)-C(6) 1.4951(18)

C(5)-H(5A) 0.9900

C(5)-H(5B) 0.9900

C(6)-C(8) 1.3692(18)

C(6)-C(7) 1.4213(19)

C(8)-C(9) 1.4894(19)

C(9)-H(9A) 0.9800

C(9)-H(9B) 0.9800

C(9)-H(9C) 0.9800

C(11)-C(12) 1.504(2)

C(11)-H(11A) 0.9900

C(11)-H(11B) 0.9900

C(12)-H(12A) 0.9800

C(12)-H(12B) 0.9800

C(12)-H(12C) 0.9800

C(13)-C(18) 1.392(2)

C(13)-C(14) 1.3961(19)

C(14)-C(15) 1.387(2)

C(14)-H(14A) 0.9500

C(15)-C(16) 1.378(2)

C(15)-H(15A) 0.9500

C(16)-C(17) 1.386(2)

C(16)-H(16A) 0.9500

C(17)-C(18) 1.387(2)

C(17)-H(17A) 0.9500

C(18)-H(18A) 0.9500

C(2)-S(1)-C(1) 90.12(7)

C(10)-O(2)-C(11) 116.52(12)

C(1)-N(1)-C(3) 109.93(12)

C(8)-N(2)-N(3) 108.33(10)

C(8)-N(2)-H(2N) 125.8(12)

N(3)-N(2)-H(2N) 122.6(12)

N(2)-N(3)-C(7) 108.45(10)

N(2)-N(3)-C(13) 121.36(10)

C(7)-N(3)-C(13) 130.16(11)

N(1)-C(1)-C(4) 124.96(14)

N(1)-C(1)-S(1) 114.34(11)

C(4)-C(1)-S(1) 120.65(10)

C(3)-C(2)-S(1) 109.08(11)

C(3)-C(2)-H(2) 125.5

S(1)-C(2)-H(2) 125.5

C(2)-C(3)-N(1) 116.54(13)

C(2)-C(3)-C(10) 124.62(13)

N(1)-C(3)-C(10) 118.82(12)

C(1)-C(4)-C(5) 112.70(11)

C(1)-C(4)-H(4A) 109.1

C(5)-C(4)-H(4A) 109.1

C(1)-C(4)-H(4B) 109.1

C(5)-C(4)-H(4B) 109.1

H(4A)-C(4)-H(4B) 107.8

C(6)-C(5)-C(4) 115.07(11)

C(6)-C(5)-H(5A) 108.5

C(4)-C(5)-H(5A) 108.5

C(6)-C(5)-H(5B) 108.5

C(4)-C(5)-H(5B) 108.5

H(5A)-C(5)-H(5B) 107.5

C(8)-C(6)-C(7) 107.49(11)

C(8)-C(6)-C(5) 127.05(12)

C(7)-C(6)-C(5) 125.33(11)

O(3)-C(7)-N(3) 123.75(12)

O(3)-C(7)-C(6) 130.48(11)

N(3)-C(7)-C(6) 105.77(11)

N(2)-C(8)-C(6) 109.88(12)

N(2)-C(8)-C(9) 120.25(11)

C(6)-C(8)-C(9) 129.84(12)

C(8)-C(9)-H(9A) 109.5

C(8)-C(9)-H(9B) 109.5

H(9A)-C(9)-H(9B) 109.5

C(8)-C(9)-H(9C) 109.5

H(9A)-C(9)-H(9C) 109.5

H(9B)-C(9)-H(9C) 109.5

O(1)-C(10)-O(2) 124.74(13)

O(1)-C(10)-C(3) 125.12(13)

O(2)-C(10)-C(3) 110.13(12)

O(2)-C(11)-C(12) 106.79(13)

O(2)-C(11)-H(11A) 110.4

C(12)-C(11)-H(11A) 110.4

O(2)-C(11)-H(11B) 110.4

C(12)-C(11)-H(11B) 110.4

H(11A)-C(11)-H(11B) 108.6

C(11)-C(12)-H(12A) 109.5

C(11)-C(12)-H(12B) 109.5

H(12A)-C(12)-H(12B) 109.5

C(11)-C(12)-H(12C) 109.5

H(12A)-C(12)-H(12C) 109.5

H(12B)-C(12)-H(12C) 109.5

C(18)-C(13)-C(14) 120.34(13)

C(18)-C(13)-N(3) 119.62(11)

C(14)-C(13)-N(3) 120.04(12)

C(15)-C(14)-C(13) 118.99(14)

C(15)-C(14)-H(14A) 120.5

C(13)-C(14)-H(14A) 120.5

C(16)-C(15)-C(14) 121.20(13)

C(16)-C(15)-H(15A) 119.4

C(14)-C(15)-H(15A) 119.4

C(15)-C(16)-C(17) 119.39(14)

C(15)-C(16)-H(16A) 120.3

C(17)-C(16)-H(16A) 120.3

C(16)-C(17)-C(18) 120.72(14)

C(16)-C(17)-H(17A) 119.6

C(18)-C(17)-H(17A) 119.6

C(17)-C(18)-C(13) 119.36(13)

C(17)-C(18)-H(18A) 120.3

C(13)-C(18)-H(18A) 120.3

Table 15. Torsion angles [°] for **51**.

__________________________________________________________________________________________________________

C(8)-N(2)-N(3)-C(7) 1.74(14)

C(8)-N(2)-N(3)-C(13) 179.92(12)

C(3)-N(1)-C(1)-C(4) 177.20(12)

C(3)-N(1)-C(1)-S(1) -0.18(14)

C(2)-S(1)-C(1)-N(1) 0.35(11)

C(2)-S(1)-C(1)-C(4) -177.16(11)

C(1)-S(1)-C(2)-C(3) -0.39(11)

S(1)-C(2)-C(3)-N(1) 0.39(16)

S(1)-C(2)-C(3)-C(10) -177.90(10)

C(1)-N(1)-C(3)-C(2) -0.14(17)

C(1)-N(1)-C(3)-C(10) 178.25(11)

N(1)-C(1)-C(4)-C(5) -65.24(17)

S(1)-C(1)-C(4)-C(5) 111.98(12)

C(1)-C(4)-C(5)-C(6) -44.18(16)

C(4)-C(5)-C(6)-C(8) 103.46(16)

C(4)-C(5)-C(6)-C(7) -81.04(17)

N(2)-N(3)-C(7)-O(3) -179.34(12)

C(13)-N(3)-C(7)-O(3) 2.7(2)

N(2)-N(3)-C(7)-C(6) -0.05(14)

C(13)-N(3)-C(7)-C(6) -178.02(12)

C(8)-C(6)-C(7)-O(3) 177.60(14)

C(5)-C(6)-C(7)-O(3) 1.4(2)

C(8)-C(6)-C(7)-N(3) -1.63(15)

C(5)-C(6)-C(7)-N(3) -177.86(12)

N(3)-N(2)-C(8)-C(6) -2.82(15)

N(3)-N(2)-C(8)-C(9) 175.45(12)

C(7)-C(6)-C(8)-N(2) 2.77(16)

C(5)-C(6)-C(8)-N(2) 178.92(13)

C(7)-C(6)-C(8)-C(9) -175.28(14)

C(5)-C(6)-C(8)-C(9) 0.9(2)

C(11)-O(2)-C(10)-O(1) -1.6(2)

C(11)-O(2)-C(10)-C(3) 177.52(11)

C(2)-C(3)-C(10)-O(1) 173.58(15)

N(1)-C(3)-C(10)-O(1) -4.7(2)

C(2)-C(3)-C(10)-O(2) -5.55(19)

N(1)-C(3)-C(10)-O(2) 176.20(11)

C(10)-O(2)-C(11)-C(12) 178.49(12)

N(2)-N(3)-C(13)-C(18) -157.65(12)

C(7)-N(3)-C(13)-C(18) 20.1(2)

N(2)-N(3)-C(13)-C(14) 21.49(19)

C(7)-N(3)-C(13)-C(14) -160.76(14)

C(18)-C(13)-C(14)-C(15) 0.0(2)

N(3)-C(13)-C(14)-C(15) -179.12(13)

C(13)-C(14)-C(15)-C(16) -0.5(2)

C(14)-C(15)-C(16)-C(17) 0.5(2)

C(15)-C(16)-C(17)-C(18) 0.0(2)

C(16)-C(17)-C(18)-C(13) -0.5(2)

C(14)-C(13)-C(18)-C(17) 0.5(2)

N(3)-C(13)-C(18)-C(17) 179.64(12)

Table 16. Hydrogen bonds for d2524a_a [Å and °].

____________________________________________________________________________

D-H...A d(D-H) d(H...A) d(D...A) <(DHA)

____________________________________________________________________________

N(2)-H(2N)...O(3)#1 0.898(18) 1.872(19) 2.7469(14) 164.2(17)

____________________________________________________________________________

# **5. Stacked LC report of 74 with hexameric peptides**

Acq. Operator : Seq. Line : 4

Acq. Instrument : Analytical Location : Vial 62

Injection Date : 4/7/2025 8:30:33 PM Inj : 1

Inj Volume : 5.0 µl

Different Inj Volume from Sequence ! Actual Inj Volume : 1.0 µl

Acq. Method : C:\CHEM32\1\DATA\APRIL 2025\MACRO DEF SEQ 2025-04-07 20-05-44\DIEGO-LC-25MINS V2.M

Last changed : 11/1/2023 4:54:30 PM

Analysis Method : C:\CHEM32\1\METHODS\JEN-LC-FAST-LOWMW WHEN LOW PRESSURE.M

Last changed : 4/25/2025 2:33:25 PM

(modified after loading)

Method Info : Default LC-MS Method

Go to "Method -> Save Method As" to save this method as your own. Following that, change the parameters to suit your needs.

=====================================================================

Module Type Firmware rev. Serial number

--------------------------------------|------|---------------|---------------

1100/1200 Column Thermostat G1316A A.07.02 [001] DE90381045

1200 Variable Wavelength Detector G1314D B.07.35 [0002] DEABB01009

1100/1200 Thermostatted Autosampler G1329A A.06.54 [003] DE33211279

1100/1200 Quaternary Pump G1311A A.07.01 [001] DEABI02104 Agilent G6130B MSD G6130B 3.02.48 US10245001

Software Revision: Rev. B.04.03 [16] Copyright © Agilent Technologies =====================================================================

Instrument Conditions : At Start At Stop

Column Temp. (left) : 26.7 26.7 °C

Column Temp. (right) : 27.2 27.2 °C

Pressure : 165.4 68.2 bar

Flow : 1.000 1.000 ml/min

Detector Lamp Burn Times: Current On-Time Accumulated On-Time VWD 1, UV Lamp : 0.48 726.7 h

Solvent Description :

PMP1 , Solvent A : ddH2O + 0.1% Formic Acid PMP1 , Solvent B : MeCN + 0.1% Formic Acid

PMP1 , Solvent C :

PMP1 , Solvent D :

=====================================================================

MSD parameters

Tune file name : C:\Chem32\1\6130BTUN\atunes.tun Ionization mode : ES-API

MSD Instrument Conditions : At Start At Stop

Quad Temp : 100 100 C

Gas Temp : 350 350 C

RoughVac : 2 2 Torr

HighVac : 2.0E-009 2.0E-009 Torr

CapCur : 4 5 nA

ChamCur : 8.0E-002 1.0E-001 µA

DryingGas : 10 10 l/min

Neb Pres : 35 35 psig

Turbo1Spd : 100 100 %

Turbo1Pwr : 131 131 W

RF Drive : 1 33 %

Qd TpDrv : 24 24 %

Gas TpDrv : 39 39 %

Neb PrDrv : 46 46 %

Gas FlDrv : 59 59 %

=====================================================================

MSD tuning (calibration) parameters

Ionization polarity : Positive

Skim1 : 35 V

Skim2 : Not Applicable

Ion Energy : 5.0 V

Lens1 : 2.2 V

Lens2 : Not Applicable

Iris : -400 V

HED : 10000 V

Width Gain : 1179

Width Offset : Variable

Mass : Value

--------------------------

118.08 : 0

622.03 : 123

922.01 : 136

1521.97 : 270

2121.93 : 342

--------------------------

Mass Gain : 76.80

Mass Offset : Variable

Mass : Value

--------------------------

118.08 : 0.808

622.03 : 0.858

922.01 : 0.846

1521.97 : 0.804

2121.93 : 0.808

--------------------------

Quad DC : 0.00 V

Octopole Peak : 650 V

Octopole Knee : Not Applicable

Lens2DC : Variable

Mass : Value

--------------------------

50.00 : 0.5 100.00 : 1.0

350.00 : 2.0

1000.00 : 4.0

2000.00 : 6.0

3000.00 : 8.0

--------------------------

L2RFEn : 1

L2RFPh : 180

L2RFAmp : Variable

Mass : Value

--------------------------

118.08 : 75

622.03 : 175

922.01 : 220

2121.93 : 345

--------------------------

Mass Filter : Gaussian

Mass Filter Width : 0.30 Da

Time Filter : Gaussian

Time Filter Width : 0.050 minutes

Ionization polarity : Negative

Skim1 : 35 V

Skim2 : Not Applicable

Ion Energy : 5.0 V

Lens1 : -4.1 V

Lens2 : Not Applicable

Iris : 400 V

HED : 10000 V

Width Gain : 1184

Width Offset : Variable

Mass : Value

--------------------------

112.99 : 26

601.98 : 53

1033.99 : 36

1633.95 : 10

2233.91 : 36

--------------------------

Mass Gain : 76.80

Mass Offset : Variable

Mass : Value

--------------------------

112.99 : 0.836

601.98 : 0.870

1033.99 : 0.858

1633.95 : 0.812

2233.91 : 0.836

--------------------------

Quad DC : 0.00 V

Octopole Peak : 650 V

Octopole Knee : Not Applicable

Lens2DC : Variable

Mass : Value

--------------------------

50.00 : 0.5 100.00 : 1.0

350.00 : 2.0

1000.00 : 4.0

2000.00 : 6.0

3000.00 : 8.0

--------------------------

L2RFEn : 1

L2RFPh : 198

L2RFAmp : Variable

Mass : Value

--------------------------

112.99 : 75

601.98 : 125

1033.99 : 175

2233.91 : 250

--------------------------

Mass Filter : Gaussian

Mass Filter Width : 0.30 Da

Time Filter : Gaussian

Time Filter Width : 0.050 minutes

=====================================================================

Run Logbook

=====================================================================

25 Apr 25 02:34 PM

Logbook File:C:\CHEM32\...5\MACRO DEF SEQ 2025-04-07 20-05-44\062-0401.D\RUN.LOG

Module # Event Message Time Date

-----------------------------------------------------------------------------

Method Method started: line# 4 vial# 62 inj# 1 20:30:10 04/07/25

Method Instrument running sample Vial 62 20:30:14 04/07/25

PUMP Pressure = 165.4 bar 20:31:22 04/07/25

THM Column temperature = 26.7 °C 20:31:22 04/07/25

THM Column temperature = 26.7 °C 20:57:52 04/07/25

PUMP Pressure = 68.2 bar 20:57:52 04/07/25

Method Saving Method DIEGO-LC-25MINS V2.M 20:57:59 04/07/25

CP Macro Analyzing rawdata 062-0401.D 20:58:00 04/07/25


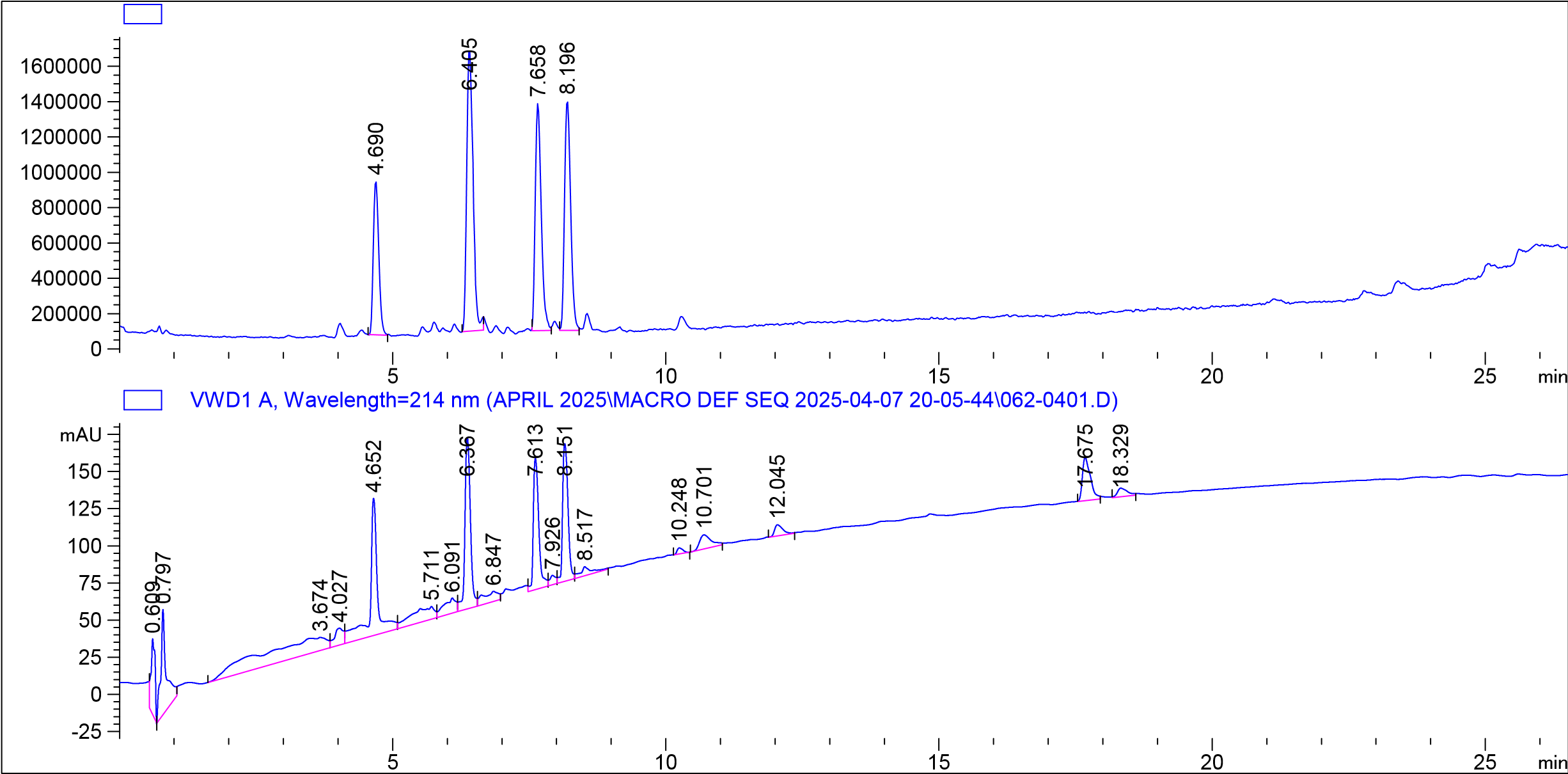
Method Instrument run completed 20:58:52 04/07/25

Method Method completed 20:58:56 04/07/25

=====================================================================

MSD1 TIC, MS File (C:\CHEM32\1\DATA\APRIL 2025\MACRO DEF SEQ 2025-04-07 20-05-44\062-0401.D) ES-API, Pos, Scan, Fra

=====================================================================

Area Percent Report =====================================================================

Sorted By : Signal

Multiplier: : 1.0000

Dilution: : 1.0000

Use Multiplier & Dilution Factor with ISTDs

Signal 1: MSD1 TIC, MS File

Peak RetTime Type Width Area Height Area

# [min] [min] %

----|-------|----|-------|----------|----------|--------|

1. 4.690 BB 0.1133 6.09541e6 8.73363e5 15.6293
2. 6.405 BB 0.1299 1.27671e7 1.60645e6 32.7362
3. 7.658 BB 0.1217 9.92128e6 1.28849e6 25.4393
4. 8.196 BB 0.1234 1.02161e7 1.30255e6 26.1953

Totals : 3.89999e7 5.07085e6

Signal 2: VWD1 A, Wavelength=214 nm

Peak RetTime Type Width Area Height Area

# [min] [min] [mAU*s] [mAU] %

----|-------|----|-------|----------|----------|--------|

1. 0.609 BV 0.0676 258.60617 50.59507 4.1432
2. 0.797 VB 0.0979 507.16718 70.55548 8.1254
3. 3.674 BV 1.3629 1025.89490 8.89913 16.4360
4. 4.027 VV 0.1702 134.40720 11.34451 2.1534
5. 4.652 VB 0.1400 917.28815 92.22477 14.6960
6. 5.711 BV 0.4042 279.56125 8.40135 4.4789
7. 6.091 VV 0.2013 163.62444 10.19128 2.6214
8. 6.367 VV 0.1085 814.56738 115.15361 13.0503
9. 6.847 VV 0.2520 137.75873 6.86804 2.2071
10. 7.613 BV 0.1087 610.77631 88.33727 9.7853
11. 7.926 VV 0.1072 48.40907 6.33163 0.7756
12. 8.151 VV 0.1100 635.66150 92.67440 10.1840
13. 8.517 VB 0.1867 85.59500 5.91941 1.3713
14. 10.248 BB 0.1052 29.94349 4.10060 0.4797
15. 10.701 BB 0.2207 141.55637 9.45203 2.2679
16. 12.045 BB 0.1704 79.47144 7.45587 1.2732
17. 17.675 BB 0.1442 295.99036 29.20669 4.7421
18. 18.329 BB 0.1814 75.47737 5.81472 1.2092

Totals : 6241.75628 623.52584

=====================================================================


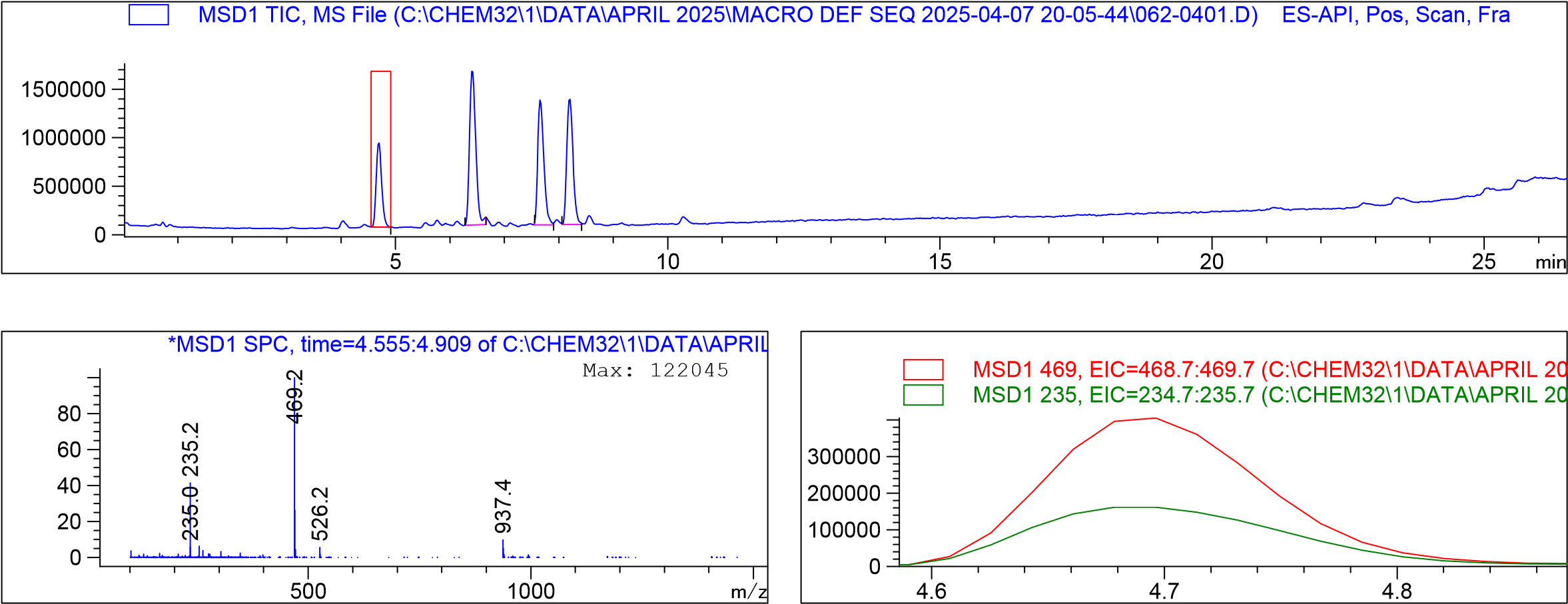


Peak #1 at 4.690 min ( 4.550 to 4.909 min)

-> The analysis found only one component, indicating a pure peak. <

Component 1: Peak at Scan 264.6. Top ions are 469 235 470

______________________________________________________________________________________________


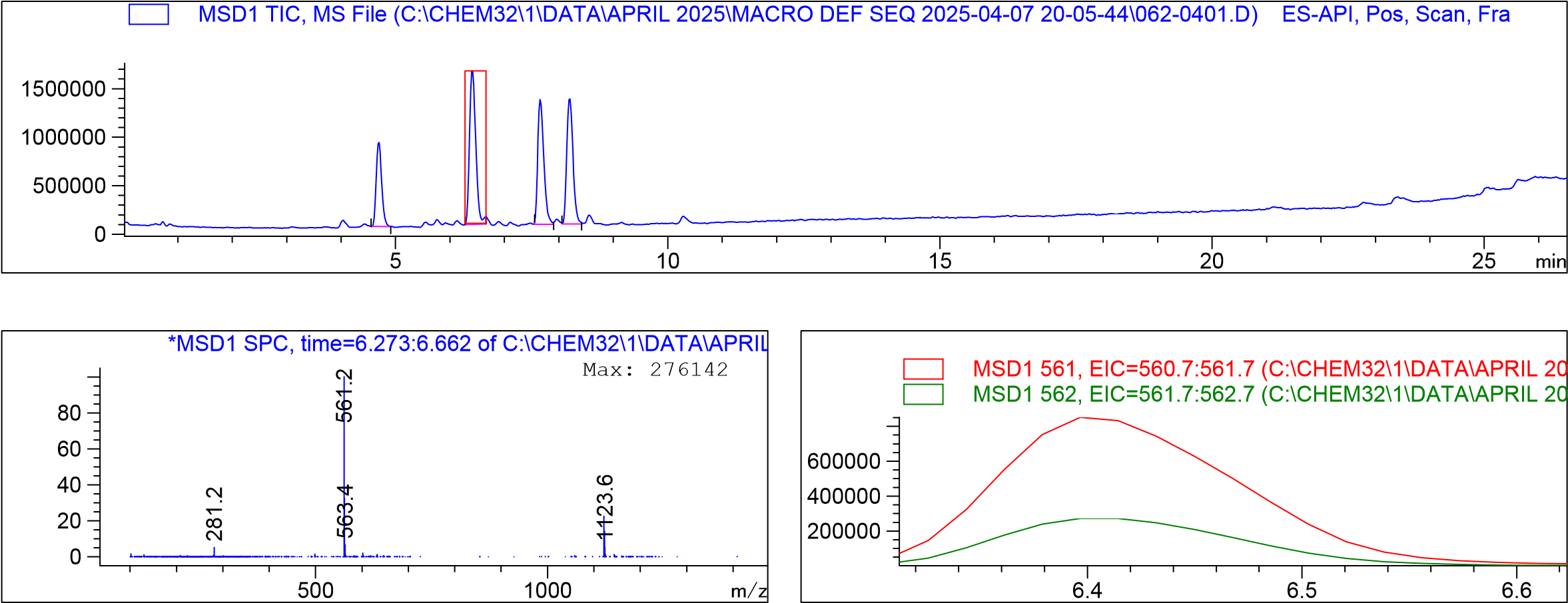


Peak #2 at 6.405 min ( 6.273 to 6.662 min)

-> The analysis found only one component, indicating a pure peak. <

Component 1: Peak at Scan 361.7. Top ions are 561 562 1121

______________________________________________________________________________________________


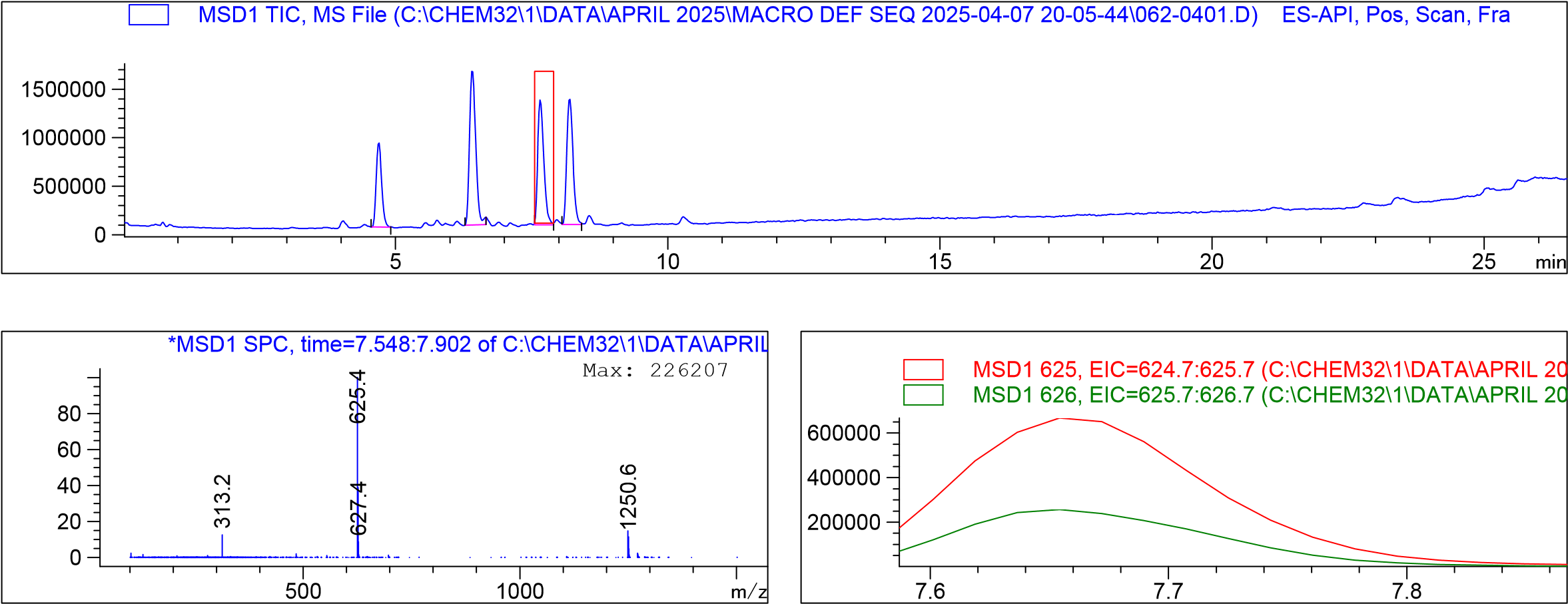


Peak #3 at 7.658 min ( 7.552 to 7.902 min)

-> The analysis found only one component, indicating a pure peak. <

Component 1: Peak at Scan 432.2. Top ions are 625 626 1249

______________________________________________________________________________________________


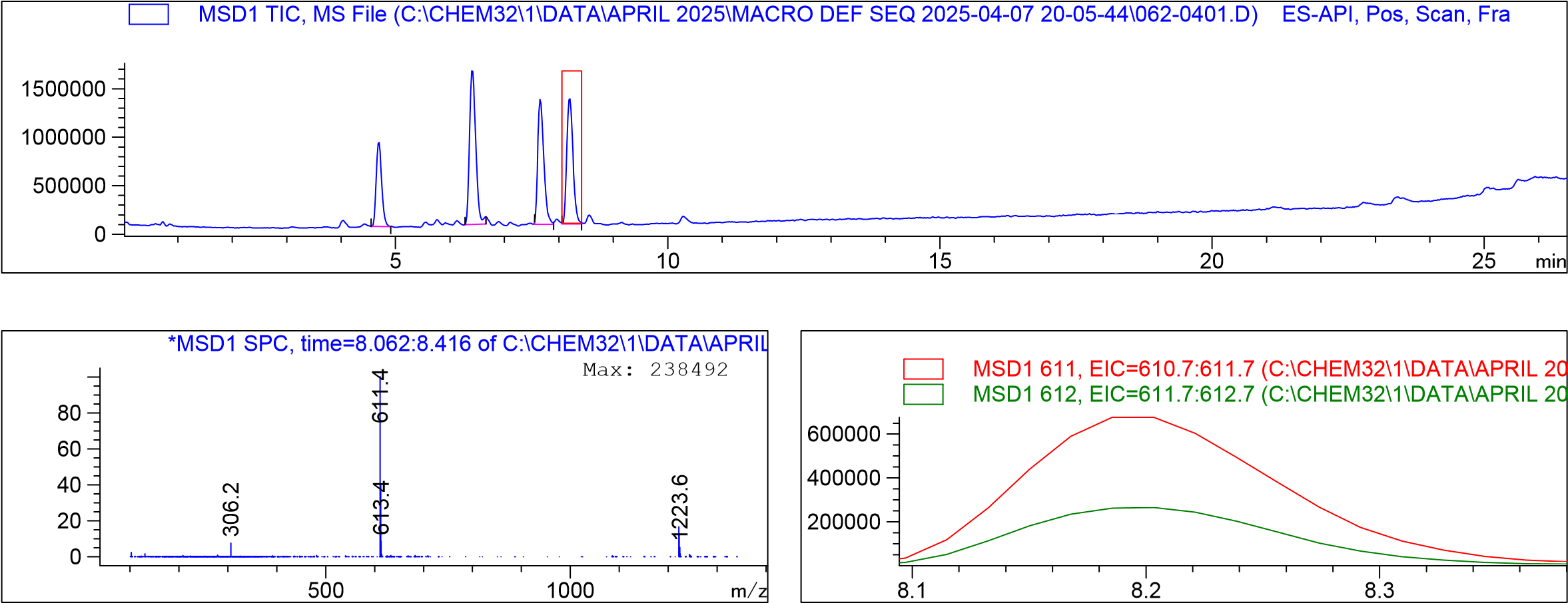


Peak #4 at 8.196 min ( 8.059 to 8.416 min)

-> The analysis found only one component, indicating a pure peak. <

Component 1: Peak at Scan 462.8. Top ions are 611 612 1221

______________________________________________________________________________________________

# **6. References**

[1] I. W. Davies, M. Taylor, J. F. Marcoux, J. Wu, P. G. Dormer, D. Hughes, P. J. Reider, “Preparation and novel reduction reactions of vinamidinium salts” *Journal of Organic Chemistry* **2001**, *66*, 251–255.

[2] R. S. Thombal, Y. R. Lee, “Synergistic Indium and Silver Dual Catalysis: A Regioselective [2 + 2 + 1]-Oxidative N -Annulation Approach for the Diverse and Polyfunctionalized N -Arylpyrazoles” *Org Lett* **2018**, *20*, 4681–4685.

[3] N. Zhang, L. Thomas, B. Wu, “Palladium-Catalyzed Selective Cross-Coupling between 2-Bromopyridines and Aryl Bromides” *J Org Chem* **2001**, *66*, 1500–1502.

[4] A. S. Demir, M. Emrullahoglu, “An effective new synthesis of 2-aminopyrrole-4-carboxylates” *Tetrahedron* **2005**, *61*, 10482–10489.

[5] C. Wang, P. Dam, M. Elghobashy, A. Brückner, J. Rabeah, L. M. Azofra, O. El-Sepelgy, “Biomimetic Dehydroamination of Primary Amines” *ACS Catal* **2023**, *13*, 14205–14212.
